# Interleukin-22 orchestrates a pathological endoplasmic reticulum stress response transcriptional programme in colonic epithelial cells

**DOI:** 10.1136/gutjnl-2019-318483

**Published:** 2019-12-02

**Authors:** Nick Powell, Eirini Pantazi, Polychronis Pavlidis, Anastasia Tsakmaki, Katherine Li, Feifei Yang, Aimee Parker, Carmen Pin, Domenico Cozzetto, Danielle Minns, Emilie Stolarczyk, Svetlana Saveljeva, Rami Mohamed, Paul Lavender, Behdad Afzali, Jonathan Digby-Bell, Tsui Tjir-Li, Arthur Kaser, Joshua Friedman, Thomas T MacDonald, Gavin A Bewick, Graham M Lord

**Affiliations:** 1 School of Immunology and Microbial Sciences, King's College London, London, UK; 2 National Institute for Health Research Biomedical Research Centre at Guy’s and St Thomas’ NHS Foundation Trust and King’s College London, London, UK; 3 Division of Digestive Diseases, Faculty of Medicine, Imperial College London, London, UK; 4 Diabetes Research Group, School of Life Course Sciences, Faculty of Life Sciences and Medicine, Kings College London, London, UK; 5 Janssen Research & Development, Spring House, Pennsylvania, USA; 6 Quadram Institute Bioscience, Norwich, Norfolk, UK; 7 Department of Translational Bioinformatics, National Institute for Health Research Biomedical Research Centre at Guy’s and St Thomas’ NHS Foundation Trust and King’s College London, London, UK; 8 Division of Gastroenterology and Hepatology, Department of Medicine, Addenbrooke’s Hospital, University of Cambridge, Cambridge, UK; 9 Centre for Immunology and Infectious Disease, Bart’s & the London School of Medicine and Dentistry, Blizard Institute of Cell and Molecular Science, London, UK; 10 Faculty of Biology, Medicine and Health, University of Manchester, Manchester, UK

**Keywords:** Interleukin 22, inflammatory bowel disease, ER stress

## Abstract

**Objective:**

The functional role of interleukin-22 (IL22) in chronic inflammation is controversial, and mechanistic insights into how it regulates target tissue are lacking. In this study, we evaluated the functional role of IL22 in chronic colitis and probed mechanisms of IL22-mediated regulation of colonic epithelial cells.

**Design:**

To investigate the functional role of IL22 in chronic colitis and how it regulates colonic epithelial cells, we employed a three-dimentional mini-gut epithelial organoid system, in vivo disease models and transcriptomic datasets in human IBD.

**Results:**

As well as inducing transcriptional modules implicated in antimicrobial responses, IL22 also coordinated an endoplasmic reticulum (ER) stress response transcriptional programme in colonic epithelial cells. In the colon of patients with active colonic Crohn’s disease (CD), there was enrichment of IL22-responsive transcriptional modules and ER stress response modules. Strikingly, in an IL22-dependent model of chronic colitis, targeting IL22 alleviated colonic epithelial ER stress and attenuated colitis. Pharmacological modulation of the ER stress response similarly impacted the severity of colitis. In patients with colonic CD, antibody blockade of IL12p40, which simultaneously blocks IL12 and IL23, the key upstream regulator of IL22 production, alleviated the colonic epithelial ER stress response.

**Conclusions:**

Our data challenge perceptions of IL22 as a predominantly beneficial cytokine in IBD and provide novel insights into the molecular mechanisms of IL22-mediated pathogenicity in chronic colitis. Targeting IL22-regulated pathways and alleviating colonic epithelial ER stress may represent promising therapeutic strategies in patients with colitis.

**Trial registration number:**

NCT02749630.

## Introduction

Inflammatory bowel disease (IBD), comprising ulcerative colitis (UC) and Crohn’s disease (CD), is a paradigmatic immune-mediated inflammatory disease (IMID). IBD is characterised by excessive accumulation of immune cells in the gut and induction of complex inflammatory networks.^1^ Similar to the situation for other IMIDs, the precise cause of IBD remains elusive, and the role of individual cytokines and immune pathways can be difficult to deconvolute. Simultaneous mobilisation of anti-inflammatory and tissue restitution factors adds yet further complexity to the picture. Interleukin-22 (IL22) is a highly controversial cytokine. Currently, the prevailing view is that IL22 promotes gastrointestinal health by supporting LGR5^+^ epithelial stem cell regeneration/proliferation.^2^ This protective role is most obviously observed in animal models of intestinal epithelial injury resulting from acute, self-limiting insults. In acute colonic infections, such as *Citrobacter rodentium*, where rapid repair of colonic epithelial cells is required, IL22 plays a protective role.^3 4^ Likewise, IL22 facilitates epithelial restitution after induction of acute injury after short-term exposure to the detergent DSS, which results in abrupt tissue injury that rapidly resolves shortly after removal of the chemical insult.^5 6^ Administration of the chemotherapy agent methotrexate also induces a self-resolving mucositis characterised by acute small intestinal epithelial damage, in which IL22 plays an important restorative role.^7^ Importantly, in these examples, the primary insult is epithelial disruption, where it seems IL22 performs an important restorative role, driving epithelial proliferation and restitution. Together these data have been interpreted as indicating that IL22 could be clinically useful to promote epithelial repair in IBD and has culminated in a clinical trial evaluating the role of recombinant IL22 therapy in patients with active IBD.

However, an alternative view of IL22 is emerging to challenge this dogma, especially in the context of chronic inflammation, rather than acute, self-limiting mucosal injury. IBD is not an acute inflammatory disease, and in the majority of patients nor is it likely to be caused by a primary epithelial defect. IBD has been a major beneficiary of the genome wide association studies (GWAS) revolution, and while disease risk conferring polymorphisms at epithelial loci are recognised, the majority of them localise at immune genes. Some preclinical models of IBD indicate that IL22 may actually contribute to disease.^8–11^ Moreover, blockade of IL23, the key upstream cytokine responsible for triggering IL22 production, looks to be very promising in early phase clinical studies in IBD.^12 13^ Indeed, high serum levels of IL22 predict response to anti-IL23 treatment.^13^ Consequently, additional insights into the role of IL22 in chronic colitis are urgently needed to inform therapeutic strategy, especially now that clinical trials evaluating the efficacy of recombinant IL22 administration to patients with active IBD are starting to be pursued.

Accordingly, there is a pressing need for new insights into the role of IL22 in chronic inflammation. Importantly, the IL22 receptor is exclusively expressed by epithelial cells in the gut, and in IBD, there is an especially compelling case to probe interactions between IL22 and the colonic epithelium, since the colon is exclusively affected in UC and affects most patients with CD.^1^ In this study, we have exploited colonic epithelial organoids, in vivo disease models and tissue transcriptomics in a large datasets of CD patients with active colitis to probe IL22-colonic epithelial interactions and provide mechanistic insights into this critical dialogue.

## Methods

Experimental methods, including in vivo treatment, cell isolation protocols, organoid cultures, gene expression profiling, immunoblotting, immunohistochemistry, fluorescence activated cell sorting (FACs), details of the UNITI trial programme and statistical methods are shown in online supplementary methods.

10.1136/gutjnl-2019-318483.supp1 Supplementary data

## Results

### IL22 regulates an endoplasmic reticulum (ER) stress response transcriptional module in colonic epithelial cells, which is augmented by IL17A

**Figure 1:**
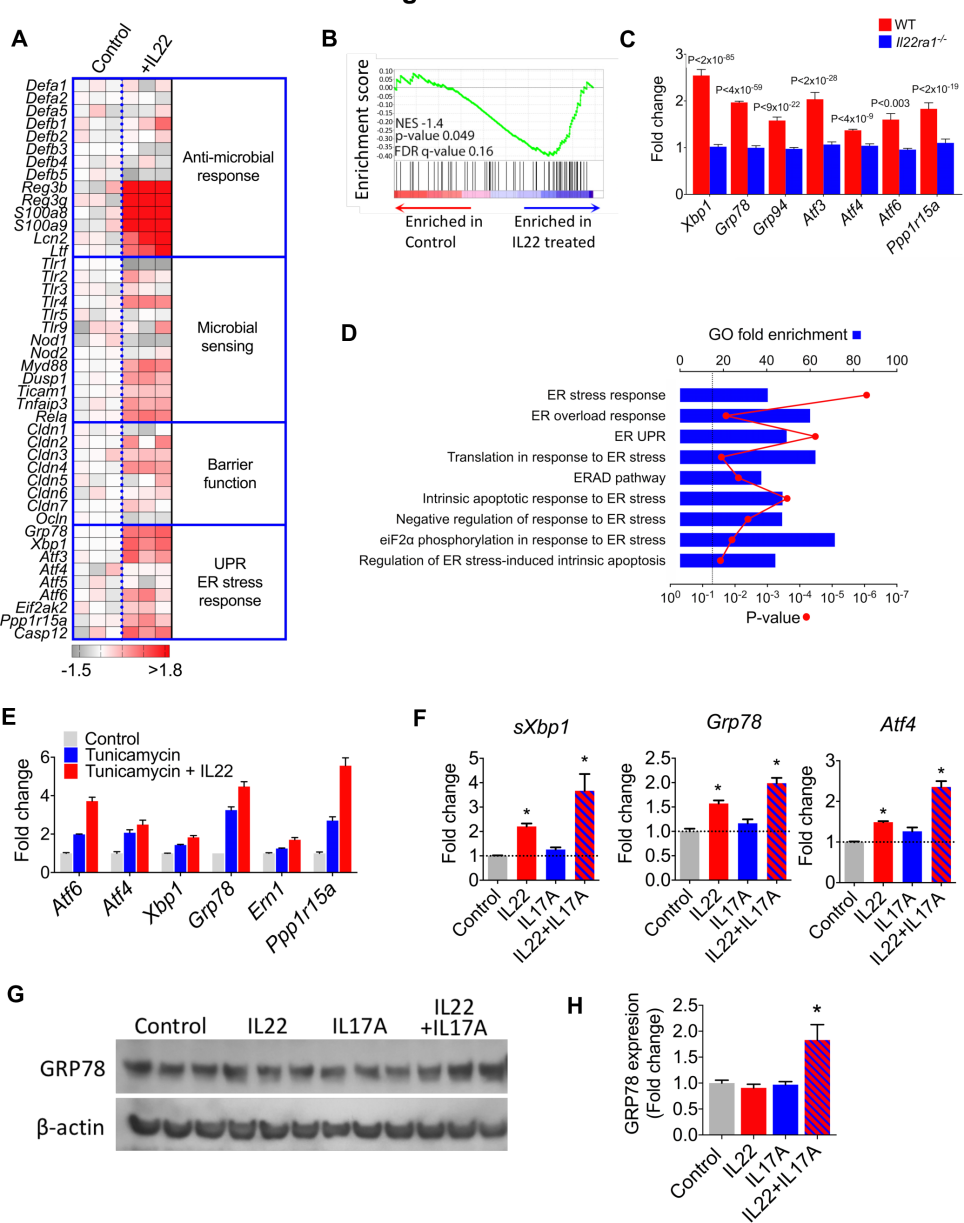
IL22 induces an ER stress/unfolded protein response transcriptional module in colonic epithelial cells. (A) Heat map demonstrating pathway specific transcript expression in murine colonoids treated with (+IL22, n=3) or without (control, n=3) recombinant IL22. Mouse gene 2.0 ST array platform (affymetrix). (B) GSEA evaluating enrichment of ER stress response transcriptional module in IL22 treated colonoids. A core set of colonic epithelial-specific ER stress genes was defined by analysing significantly differentially expressed (p<0.05 and absolute value of the log2 fold change >±2) transcripts in colonoids treated with tunicamycin (n=3) or medium alone (n=3). (C) Expression of ER stress response transcripts in IL22 treated WT and *Il22ra1^−/−^* colonoids (RNA-seq dataset ERR247358-ERR247389, Pham *et al*, 2014).^18^ (D) Enrichment analysis for ER stress-related functional annotation groups (GO biological processes) in IL22-treated colonoids from dataset ERR247358-ERR247389. (E) Microarray analysis of core ER stress response transcripts in colonoids treated with tunicamycin (n=3), tunicamycin+IL22 (n=3) or untreated (control, n=3). (F) Real-time PCR quantification of ER stress transcripts in colonoids treated with IL22 (n=11), IL17A (n=6) and IL22+IL17A (n=6) and unexposed controls. *P<0.01. (G) Immunoblot and densitometry quantification (H) detecting GRP78 protein expression in colonoids treated with different cytokines. *P<0.026, one tailed t test. ER, endoplasmic reticulum; GO, Gene Ontology; GSEA, Gene Set Enrichment Analysis; IL22, interleukin-22.

To probe interactions between IL22 and the colonic epithelium, we exploited a colonic epithelial organoid system. Colonic crypts were harvested from mouse colons and cultured to form three-dimensional organ buds that retained phenotypic and functional characteristics of intact primary colonic epithelial cells^14^ (online supplementary figure 1). Colonic epithelial organoids (‘colonoids’) were treated with recombinant IL22 and transcriptional responses mapped by gene expression microarray analysis. In this system, IL22 modulated expression of 859 genes (online supplementary table 1), including genes encoding antimicrobial peptides, such as recognised IL22-responsive transcripts, *Reg3b* and *Reg3g*, but also other antimicrobial molecules, including the calprotectin subunits *S100a8*/*S100a9*, lipocalin-2 (*Lcn2*) and lactoferrin (*Ltf*). IL22 had no impact on the expression of α or β-defensin family antimicrobial peptides. IL22 induced significant upregulation of transcripts involved in microbial sensing (*Tlr4, Myd88, Tnfaip3*) and epithelial barrier function, including claudin family genes encoding important colonic epithelial tight junction proteins (figure 1A). Recently, IL22 has been reported to induce ER stress in small intestinal epithelial cells in vivo and in vitro, which is associated with heightened susceptibility to experimental small intestinal inflammation, especially in the context of *Atg16l1* deficiency.^11^ Infective, metabolic, toxic or inflammatory cellular insults can overwhelm protein synthesis in the ER, resulting in accumulation of potentially toxic misfolded proteins and ER stress.^15^ The unfolded protein response (UPR) is a highly conserved cellular process that functions to mitigate against the harmful effects of protein misfolding. In our colonoid system, IL22 also significantly upregulated key transcripts responsible for controlling the UPR (figure 1A and online supplementary table 1).

10.1136/gutjnl-2019-318483.supp2 Supplementary data

10.1136/gutjnl-2019-318483.supp3 Supplementary data

To further understand the transcriptional architecture of a pathological ER stress response in the colonic epithelial compartment, we treated colonoids with tunicamycin, a powerful chemical inducer of a pathological ER stress response.^16^ Tunicamycin significantly modulated the expression of 217 genes in colonoids (online supplementary table 2), and enrichment analysis for functional annotation groups demonstrated significant association with cellular processes associated with ER stress, including ‘response to unfolded protein’ and ‘response to ER stress’ (online supplementary figure 2). Gene Set Enrichment Analysis^17^ confirmed significant enrichment of the colonic epithelial compartment-specific ER stress response transcriptional module in IL22 treated colonoids (figure 1B). These data were corroborated by real time PCR, confirming that IL22 induced transcription of core ER stress response transcripts in a time and dose dependent manner (online supplementary figure 3). We validated these findings in an independent, published dataset of genome-wide transcriptional changes in colonic epithelial cells generated using a different gene expression platform (RNA sequencing).^18^ In agreement with our data, IL22 induced an ER stress response transcriptional programme in WT colonoids, but not in *Il22ra1^−/−^* colonoids, additionally confirming that this pathway is dependent on signalling through the conventional IL22 receptor (figure 1C). Comparable findings were observed at pathway level, with significant enrichment of transcripts annotated to Gene Ontology (GO) terms, such as ‘response to ER stress’ and ‘ER stress overload response’ (figure 1D).

We also observed that IL22 synergistically augmented tunicamycin-induced transcription of core ER stress genes in our microarray analysis (figure 1E and online supplementary table 3), which was corroborated by real time PCR (online supplementary figure 4), indicating that IL22 might also potentiate the ER stress response driven by other mediators. We reasoned that this might be especially important in chronic inflammation, where other proinflammatory mediators are present in the local tissue environment. IL22 is often coproduced with IL17A^19 20^; therefore, we considered the possibility that IL22 might synergise with IL17A to drive the ER stress response. By itself IL17A was only a weak inducer of ER stress-associated transcripts; however, in combination with IL22, there was stronger induction of UPR transcripts (figure 1F). Additionally, we evaluated whether IL22 and IL17A induced an ER stress response at protein level in colonoids. Western blotting for GRP78 in cytokine-treated colonoids demonstrated increased immunoreactivity for GRP78 only in colonoids treated with both IL17A and IL22 in combination (figure 1G, H).

Next, we considered whether IL17A/IL22-induced ER stress was directed at the epithelial stem cell niche or non-stem epithelial cells. To address this question, colonoids were generated from Lgr5-GFP reporter mice, permitting distinction between Lgr5^+^ colonic epithelial stem cells and the Lgr5^−^ non-stem cell epithelial compartment (online supplementary figure 5A, B). Following exposure to IL17A and IL22, colonoids were dissociated into single cell suspensions and FACS purified into GFP^+^ stem cells and GFP^−^ epithelial populations. IL22/IL17A significantly induced increased expression of *sXbp1* and *Grp78*; although the magnitude of induction was greater in the Lgr5^+^ epithelial stem cell compartment, there was also induction in the Lgr5^−^ non-stem cell epithelial compartment (online supplementary figure 5C). These results indicate that the ER stress response was directed at both stem cell and non-stem cell epithelial cells.

### IL22 and IL17A promote ER stress and intestinal epithelial apoptosis

**Figure 2:**
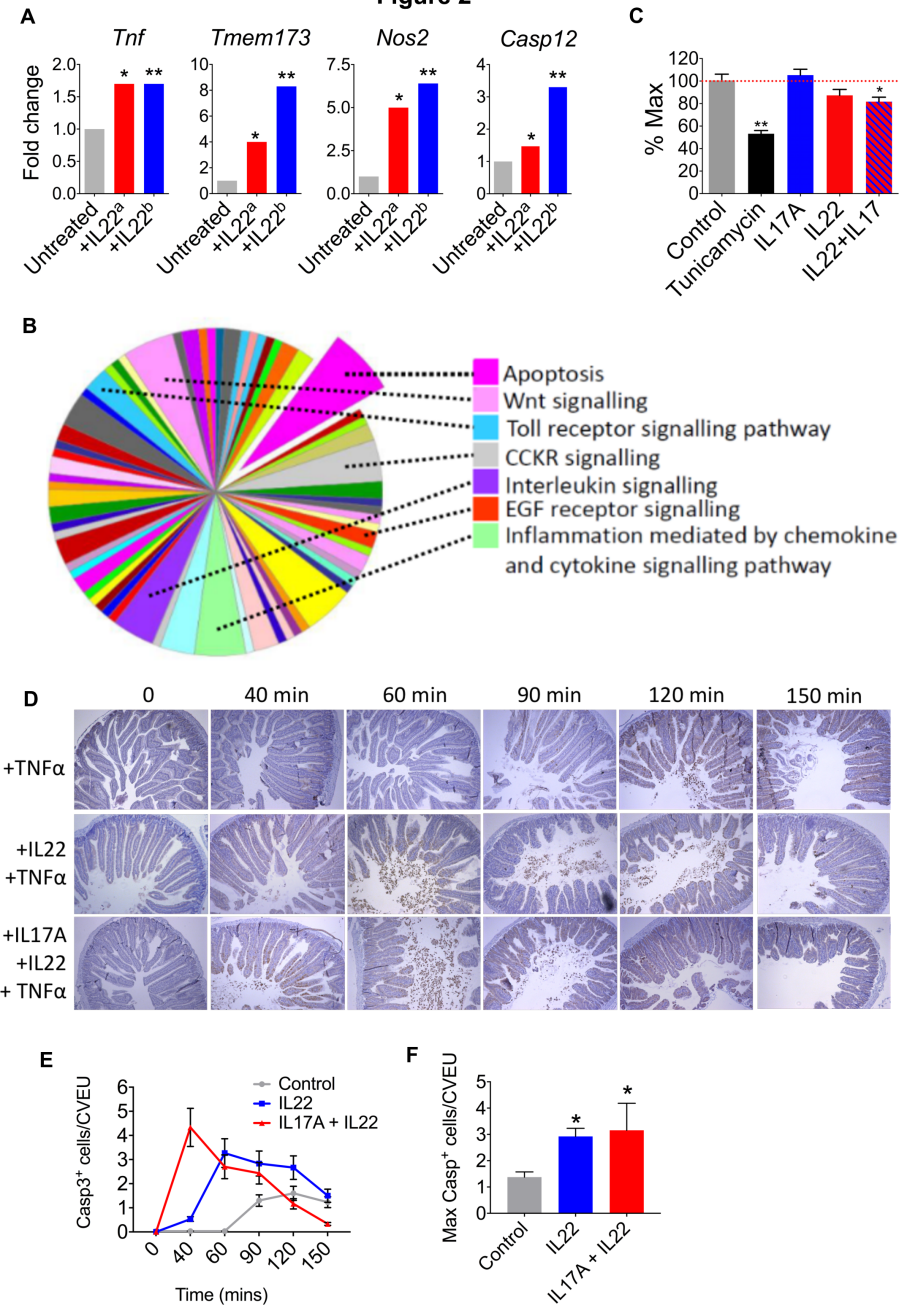
IL22 and IL17A promote ER stress and intestinal epithelial apoptosis. (A) Transcript expression in IL22-treated colonoids from our dataset^a^ and Pham *et al* ERR247358-ERR247389^b^. (B) Panther analysis of pathways activated in IL22-treated colonoids. (C) MTT assay demonstrating colonic epithelial cell viability after treatment with IL22, IL22+IL17A, or tunicamycin, versus untreated colonoids. *P<0.02, **P<0.0001. (D) In vivo model of intestinal epithelial apoptosis showing representative immunohistochemistry (caspase 3 immunoreactivity) and statistical analyses (E and F) of intestinal sections harvested at different time points following pretreatment with either IL22, IL22+IL17A, or PBS, prior to administration of TNFα. *P<0.005. IL22, interleukin-22.

Although the UPR is mobilised to restore cellular homeostasis, unresolved ER stress results in a pathological UPR response, proinflammatory signalling and induction of proapoptotic pathways.^15 21 22^ In addition to upregulation of ER stress response transcripts, we also observed upregulation of transcripts that impact the inflammatory tone of the colonic epithelium, including susceptibility to apoptosis. IL22 induced the expression of *Tnf* (a proinflammatory and proapoptotic cytokine), inducible nitric oxide synthetase (*Nos2*, which has been implicated as a trigger for epithelial inflammation, carcinogenesis and ER stress)^23 24^ and the proinflammatory/proapoptotic intracellular molecule Sting (*Tmem173*, a potent proinflammatory mediator linking intracellular pattern recognition receptors to activation of innate immunity)^25^ (figure 2A and online supplementary table 1). Indeed, Sting has previously been linked to IL22-induced apoptosis.^8–11^ There was also induction of caspase 12, a member of the caspase family that is itself anchored in the ER and is responsible for regulating ER stress-associated apoptosis^15 21 22^ (figure 2A). These findings were replicated in the independent transcriptomic dataset from Pham *et al* 18 (figure 2A).

Pathway level analyses also indicated that IL22 might prime for apoptosis in colonic organoids. Classification of gene function of all IL22-regulated transcripts (315 annotated genes with ≥1.5-fold increased expression), using protein annotation through evolutionary relationship (PANTHER) analysis^26^ demonstrated significant enrichment of expected pathways, such as ‘interleukin signalling’, ‘Inflammation mediated by chemokine and cytokine signalling pathway’ and ‘Toll-like receptor signalling’ (figure 2B). However, the single most enriched pathway was ‘apoptosis’. These findings were replicated in the Pham dataset (RNA sequencing),^18^ and in agreement with our data, pathway mapping of IL22 colonoids using the Kyoto Encyclopedia of Genes and Genomes^27^ system identified activation of comparable pathways. As expected, there was significant enrichment of pathways involved in ‘bacterial invasion of epithelial cells’, ‘tight junctions’, ‘cytokine-cytokine receptor interactions’ and ‘colorectal cancer’; however, we also observed a highly significant association with ‘apoptosis’ (p<10^−7^) (online supplementary figure 6).

Importantly, in our microarray analysis of IL22-induced gene expression changes, we observed upregulation and downregulation of different apoptotic genes; therefore, to further investigate the observed differential gene expression patterns on the activity of the apoptotic process computationally, we used the Qiagen Ingenuity Pathway Analysis. The tool considers the concordance between the directionality of the gene expression changes from an experiment and those known to activate (positive z-score) or inhibit (negative z-score) a given pathway. Predictions for whole pathways are made by assessing statistically such level of agreement. We found that apoptosis pathways were significantly, yet moderately, activated in IL22-treated mouse colonoids for apoptosis pathways, including ‘apoptosis’ (activation z-score=0.511, p=3.17^−15^) and ‘epithelial apoptosis’ (activation z-score=0.695, p=3.28^−14^).

To determine whether IL22 might impact epithelial cell viability, we performed an MTT (reduction of 3-(4,5-dimethylthiazol-2-yl)-2,5-diphenyltetrazonium bromide) viability assay. Tunicamyin induces ER stress, apoptosis and barrier disruption in intestinal epithelial cells,^16^ and as expected, there was significant loss of epithelial cell viability in colonoids treated with tunicamycin (figure 2C). Although IL17A and IL22 by themselves did not significantly impact cell viability, there was significant loss of cell viability in colonoids treated with a combination of IL22 and IL17A, although these effects were less pronounced than observed with tunicamycin (figure 2C). To determine whether IL22 and IL17A could impact on intestinal epithelial apoptosis in an in vivo setting, we assessed the impact of cytokine administration in a model of intestinal epithelial apoptosis. A single dose of tumour necrosis factor-α (TNFα) induces rapid epithelial cell apoptosis, predominantly at the villus tips, accompanied by villus shortening, fluid exudation into the gut lumen and diarrhoea.^28 29^ TNFα delivery induced enterocyte cell death and shedding, predominantly in the villus tips of the intestine (figure 2D). This response was detectable from around 90 min post-TNFα administration and was most pronounced at 120 min, with numerous shed cells visible in the lumen, and high numbers of TUNEL^+^ and caspase-3^+^ epithelial cells on the villi, which started to taper by 150 min (figure 2D–F). Pretreatment with IL22, resulted in earlier detection of intense shedding, from 60 min, while pretreatment with combined IL22/IL17A accelerated the response even further, with increased cell shedding and apoptosis observed just 40 min post-TNFα delivery (figure 2D–F). Quantification of caspase-3^+^ cells showed that the apoptotic response to TNFα was significantly increased by both IL22 and IL22/IL17 pretreatments (figure 2D–F). Taken together these data indicate that IL22, particularly in combination with IL17A, promotes colonic epithelial apoptosis. This property may be especially pronounced in chronic inflammation, when other proapoptotic factors are abundant (eg, TNFα), or indeed following unresolved ER stress response, which also culminates in apoptosis.

### IL22 is a functionally important driver of colonic ER stress in chronic colitis

**Figure 3:**
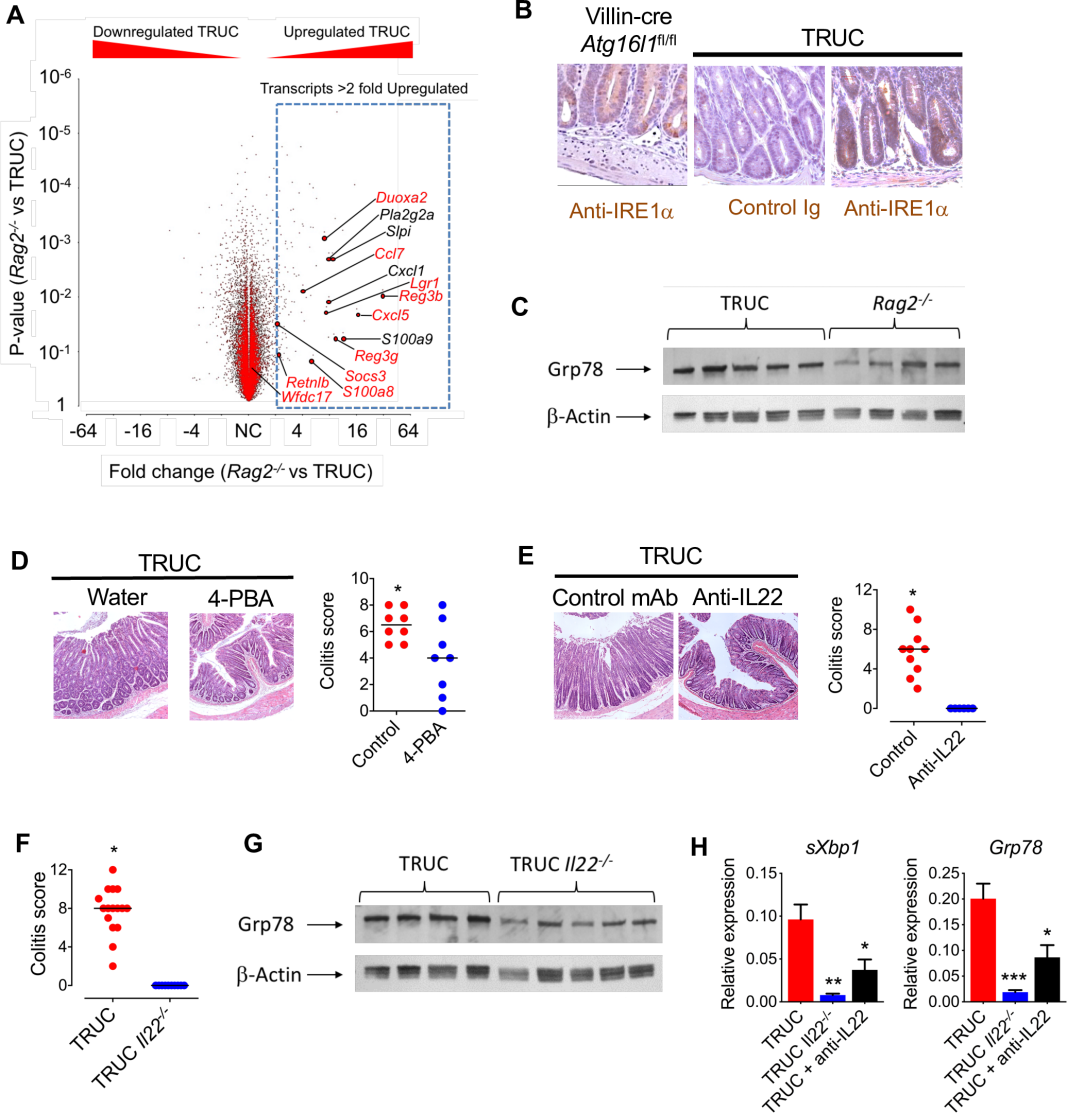
IL22/ER stress axis is functionally important in chronic colitis. (A) Volcano plot (fold change vs p value) showing gene expression in the colon of *Rag2^−/−^* (n=3) and TRUC (n=3) mice, microarray analysis (MouseWG-6 v2.0 expression BeadChip, Illumina). Transcripts annotated in red are among the top 20 most highly upregulated genes in IL22-treated colonoids. (B) Representative immunohistochemistry (IRE1α immunoreactivity) in distal colon of *villin-cre Atg16l1^fl/fl^* mice and TRUC mice. (C) Western blot of distal colon segments from TRUC and *Rag2^−/−^* mice probed with anti-GRP78. Corresponding densitometry plots are shown in online supplementary figure 7. (D) Representative histology (H&E) and histology scores of the distal colon of TRUC mice administered 4-PBA in drinking water (n=8) or water alone (n=8). P<0.025. (E) Colon micrograph (H&E stain), and colitis score of distal colon of TRUC mice treated with anti-IL22 mAb (n=6) or control antibody (n=10). P<0.001. (F) Colitis score of distal colon of TRUC (n=16) and TRUC *Il22^−/−^* (n=10) mice. P<0.0001. (G) Western blot of distal colon segments from TRUC and TRUC *Il22^−/−^* mice probed with anti-GRP78. Corresponding densitometry plots are shown in online supplementary figure 7. (H) Real-time PCR quantifying ER stress transcripts in the distal colon of TRUC mice (n=9), TRUC *Il22^−/−^* mice (n=5) and TRUC mice treated with anti-IL22 (n=6). *P<0.05, **P<0.02, ***P<0.001. 4-PBA, 4-phenylbutryic acid; IL22, interleukin-22.

Next, we asked whether the IL22/ER stress axis was functionally important in chronic colitis. To address this question, we exploited the TRUC model of IBD, which mirrors some aspects of chronic colitis in human IBD. TRUC mice develop chronic microbiota-dependent colitis, mediated by pathogenic group 3 innate lymphoid cells, and disease is alleviated following administration of anti-TNFα or anti-IL23p19 mAbs.^14 19 20^ Profiling the colonic transcriptome of TRUC mice demonstrated significant enrichment of IL22-responsive transcripts in the colon of TRUC mice. Indeed, the majority of the most highly induced transcripts in IL22 treated colonoids were additionally upregulated in the colon of TRUC mice (figure 3A). Consistent with the enrichment of ER stress response transcripts observed in the colon of TRUC mice, we also observed increased immunoreactivity of IRE1α, an endoribonuclease responsible for Xbp1 splicing, in the colonic epithelium of TRUC mice (figure 3B). As a positive control, we also observed increased IRE1α immunoreactivity in the colonic epithelium of *Villin*-cre × *Atg16l1* ^fl/fl^ mice, where impaired autophagy results in a pathological ER stress response (figure 3B). Western blotting confirmed increased expression of Grp78 protein in the colon of TRUC mice in comparison with *Rag2^−/−^* mice (figure 3C and online supplementary figure 7).

Next, we investigated the functional impact of increased epithelial ER stress in TRUC mice by pharmacological alleviation of ER stress by administering 4-phenylbutryic acid (4-PBA), an inhibitor of ER stress. 4-PBA administration reduced ER stress and significantly attenuated colitis in TRUC mice (figure 3D and online supplementary figure 8), consistent with an exaggerated ER stress response contributing to colitis severity. To further probe the functional role of the IL22 in driving epithelial ER stress in vivo, we employed genetic ablation and antibody blockade experiments. In comparison with TRUC mice, germ line genetic deletion (TRUC *Il22^−/−^*) or neutralisation (anti-IL22 mAb) significantly reduced ER stress and completely attenuated colitis (figure 3E–H). Taken together these data indicate that IL22 plays a functionally important role in chronic colitis in TRUC mice and that targeting IL22, or pathological ER stress in the epithelium, alleviates colitis.

### Local induction of ER stress re-instates colitis in otherwise healthy TRUC *Il22^−/−^* mice

**Figure 4:**
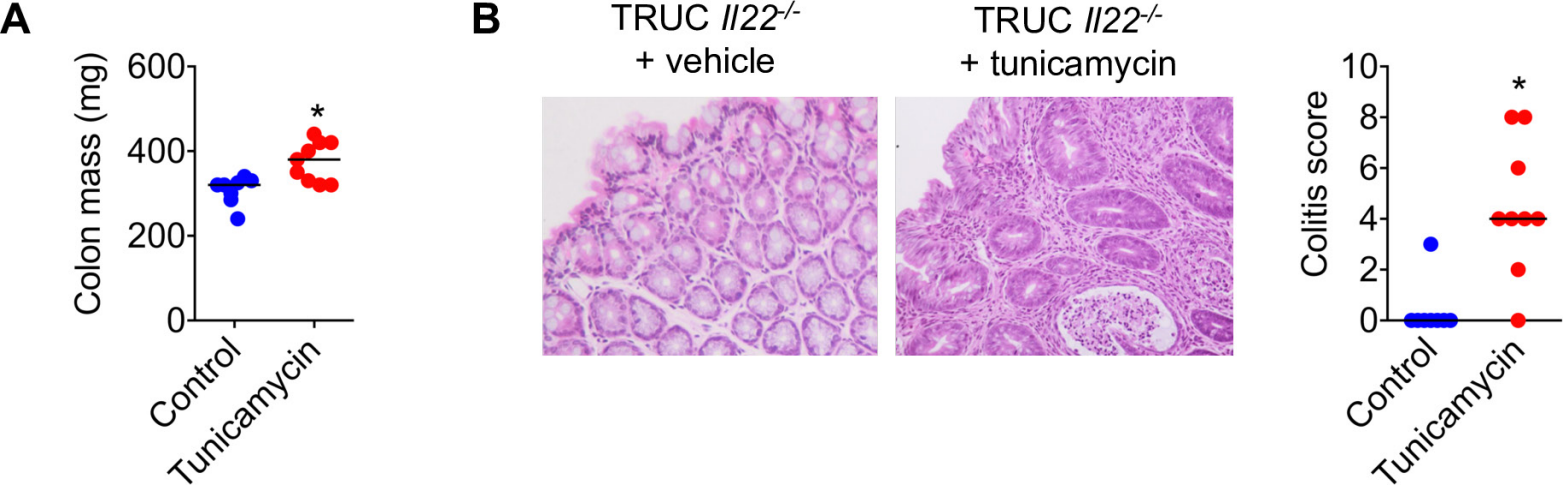
Local induction of ER stress reinstates colitis in TRUC *Il22^−/−^* mice. (A) Histological appearance (H&E stain) and colitis score of the distal colon, and (B) colon mass of TRUC mice treated with intrarectal tunicamycin (n=8) or vehicle control (n=9). *P<0.01. Bar charts depict mean and SEM in graphs showing dots, each dot represents an individual mouse.

Since TRUC *Il22^−/−^* mice do not develop colonic epithelial ER stress and are protected from colitis, we hypothesised that direct induction of colonic epithelial ER stress, even in the absence of IL22, should be sufficient to reinstate disease. To address this question, we administered tunicamycin intrarectally to healthy TRUC *Il22^−/−^* mice. Strikingly, in contrast to vehicle-treated mice, tunicamycin induced increased colonic mass and histological features of colitis in TRUC *Il22*^−/−^** mice (figure 4A, B).

### IL22-responsive transcripts are increased in the colon of patients with IBD and correlate with key biomarkers and the severity of mucosal injury

**Figure 5:**
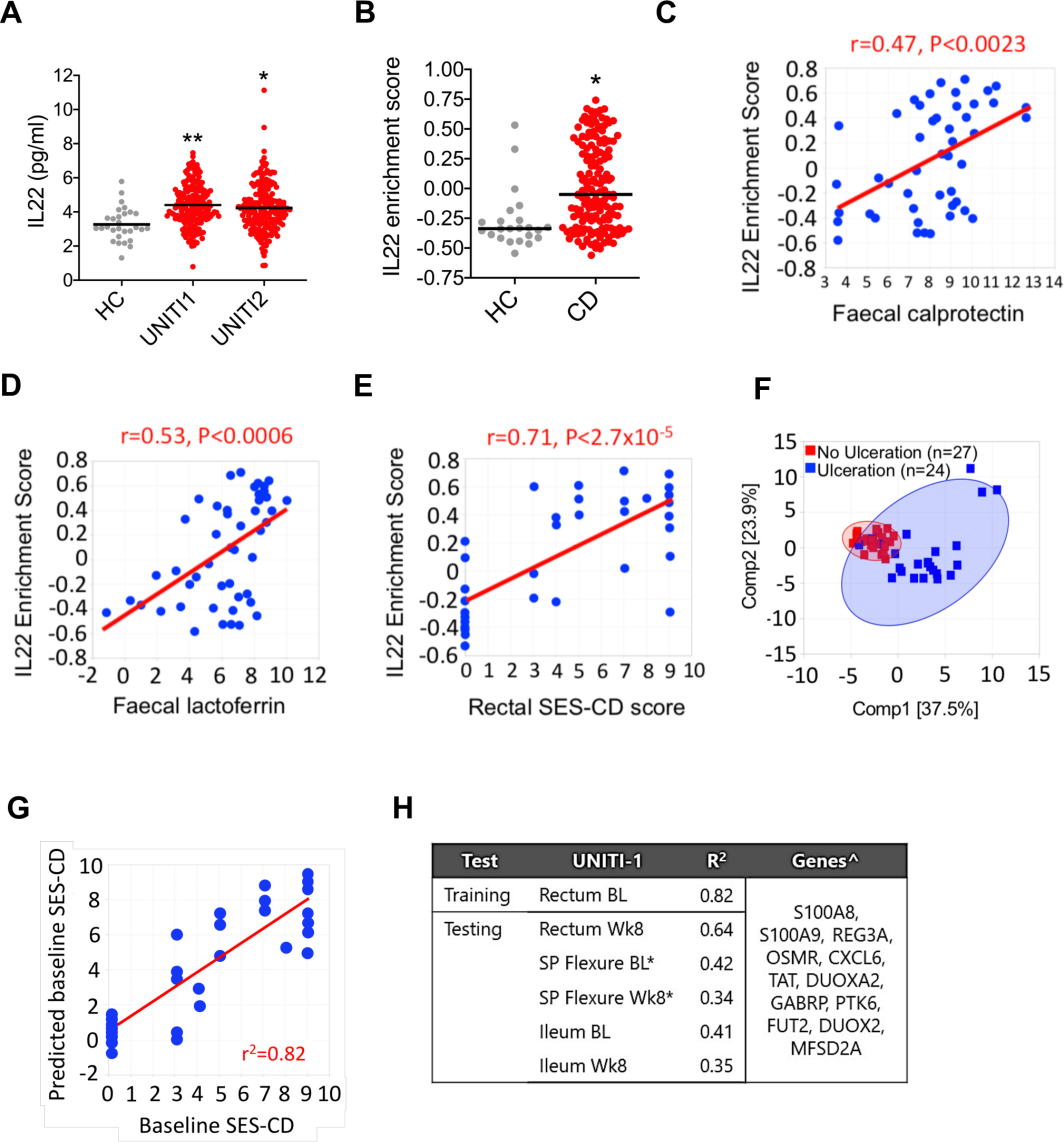
Increased expression of IL22 and IL22-regulated transcriptional modules in active colitis. (A) Serum IL22 concentration in healthy control (HC; n=29) and patients with CD from UNITI1 (n=191) and UNITI2 (n=205) trial programmes. *P<0.0005, **p<0.0001). (B) IL22 responsive transcript GSVA enrichment scores of the 20 mostly highly upregulated IL22 responsive transcripts in patients with CD (n=162) from the UNITI trial programme (at baseline prior to randomisation to placebo or ustekinumab) or HC subjects (n=23). Each dot represents an individual patient. Line depicts median. Gene expression data for the UNITI cohort were quantified using the Affymetrix Hg U133 PM array. *P<0.0003. (C) GSVA showing IL22 responsive transcript enrichment scores in colonic biopsies from patients with active (Mayo endoscopy subscore 2–3) or quiescent (Mayo endoscopy subscore 0–1) UC, CD or HCs (gene expression microarray datasets GSE50971 and GSE16879). (C–E) Correlation between IL22 enrichment score (GSVA) in rectal biopsies of patients with CD, with faecal calprotectin (C), faecal lactoferrin (D) and (E) regional SES-CD (ie, endoscopic severity of mucosal disease at same site mucosal biopsy was sampled from). Displayed is baseline rectum gene expression from UNITI-1 subpopulation with colon involvement. Faecal calprotectin and lactoferrin were log 2 transformed data. (F) PCA of baseline rectum gene expression of IL22 responsive genes in UNITI-1 segregates CD patients with and without baseline ulceration. (G) A multivariant model using IL22-responsive transcripts to predict endoscopic activity by SES-CD in UNITI cohort, with correlation of predicted and actual SES-CD score in the training set using baseline rectal biopsies in UNITI-1. (H) Coefficients of determination of the training and testing datasets in UNITI-1 and genes used in the predictive model. *Predicted SES-CD at left colon; ˆGenes sorted by selection order. bL, baseline; CD, Crohn’s disease; GSVA, Gene Set Variation Analysis; IL22, interleukin-22; R^2^, coefficient of determination; SES-CD, Simple Endoscopic Score – Crohn’s Disease; Wk8, week 8.

Next, we sought to determine whether these observations might be relevant in human colitis. We investigated the expression of IL22-responsive transcriptional networks and the ER stress response in colonic tissue from CD patients with active colitis. To address this question, we interrogated transcriptomic, serological and clinical data from a large cohort of patients with active colonic CD from the UNITI study, a large phase III trial programme evaluating the efficacy of ustekinumab, a human IgG1κ mAb targeting the p40 subunit common to both IL12 and IL23.^30^ Colonic biopsies were sampled at baseline (ie, before administration of the study drug) and longitudinally following initiation of drug. In both UNITI-1 and UNITI-2 cohorts, serum IL22 concentration was significantly increased in comparison with healthy control subjects (figure 5A). We evaluated tissue transcriptomics in rectal biopsies of patients with CD from the UNITI cohort at baseline in comparison with healthy control subjects, who were not part of the UNITI trial programme but whose biopsy gene expression data were generated and analysed in parallel. Using the Human Genome U133 Plus 2.0 Array platform, the probe sets for cytokines, including IL22, IL17A and TNF, generally hybridised at low intensity, such that comparison of cytokine transcripts between the groups was difficult to determine (data not shown). However, we reasoned that if IL22 was biologically active in diseased colonic tissue of patients with colitis, then core IL22-responsive transcripts would be enriched in rectal biopsies of CD patients with active colitis in comparison with non-inflammatory control subjects. We defined a core IL22-responsive transcriptional programme by identifying the human homologues of the 20 most highly upregulated transcripts in IL22-treated colonoids. Strikingly, Gene Set Variation Analysis (GSVA)^31^ demonstrated significant enrichment of the IL22-responsive transcriptional module (figure 5B). These findings were replicated in colonic biopsies sampled from an independent cohort of IBD patients with colonic CD and active UC (GSE16879, online supplementary figure 9).^32^ As well as demonstrating significantly higher expression levels of IL22 responsive transcripts in patients with active colonic IBD, unsupervised hierarchical clustering could fully differentiate CD and UC patients from controls (online supplementary figure 10). In the UNITI cohort extensive phenotypic data were available, including disease activity scoring, endoscopic severity scoring and measurement of inflammatory biomarkers. Crucially, enrichment scores for IL22-responsive transcripts correlated with biomarkers of disease activity and severity, including faecal lactoferrin and calprotectin concentrations (figure 5C, D). Moreover, the IL22 enrichment score in rectal biopsies significantly correlated with the severity of mucosal injury scored during endoscopy, calculated using the Simple Endoscopic Score – Crohn’s Disease (SES-CD), which quantifies endoscopically assessed severity of inflammation and is arguably the most important objective marker of CD severity/activity (figure 5D). Principal component analysis demonstrated that IL22 responsive transcripts could discriminate between the presence or absence of epithelial ulceration in the rectum identified during endoscopy (figure 5E). To further explore the correlation between IL22 responsive transcripts and severity of mucosal injury, we adopted a machine learning approach to evaluate the ability of IL22-responsive transcripts to predict endoscopic activity (SES-CD). Elastic Net regression^33^ was conducted to select genes from the IL22 signature to predict endoscopic disease severity. The rectum baseline gene expression data were used for training and those from rectum at week 8 and splenic flexure and terminal ileum at baseline and week 8 served as testing datasets. A model derived from the UNITI-1 discovery data set was predictive of the baseline SES-CD score with R2 of 0.82 (figure 5G–H). We validated the performance of this model in the testing datasets from the same study (r^2^ range between 0.35 and 0.64), with the best performance in the week 8 rectum (r^2^=0.64, figure 5H), confirming that IL22 responsive transcripts can predict severity of SES-CD in colonic CD. Finally, we asked whether the expression of the IL22-responsive transcriptional module in biopsies sampled at baseline, prior to institution of ustekinumab, could be harnessed to predict response to treatment in the UNITI cohort. Using PCA, the IL22 responsive signature could not differentiate responders and non-responders to ustekinumab in the UNITI cohort (online supplementary figure 11).

### An epithelial cell-specific ER stress-driven transcriptional programme is enriched in active colitis and correlates with the IL22 transcriptional footprint

**Figure 6:**
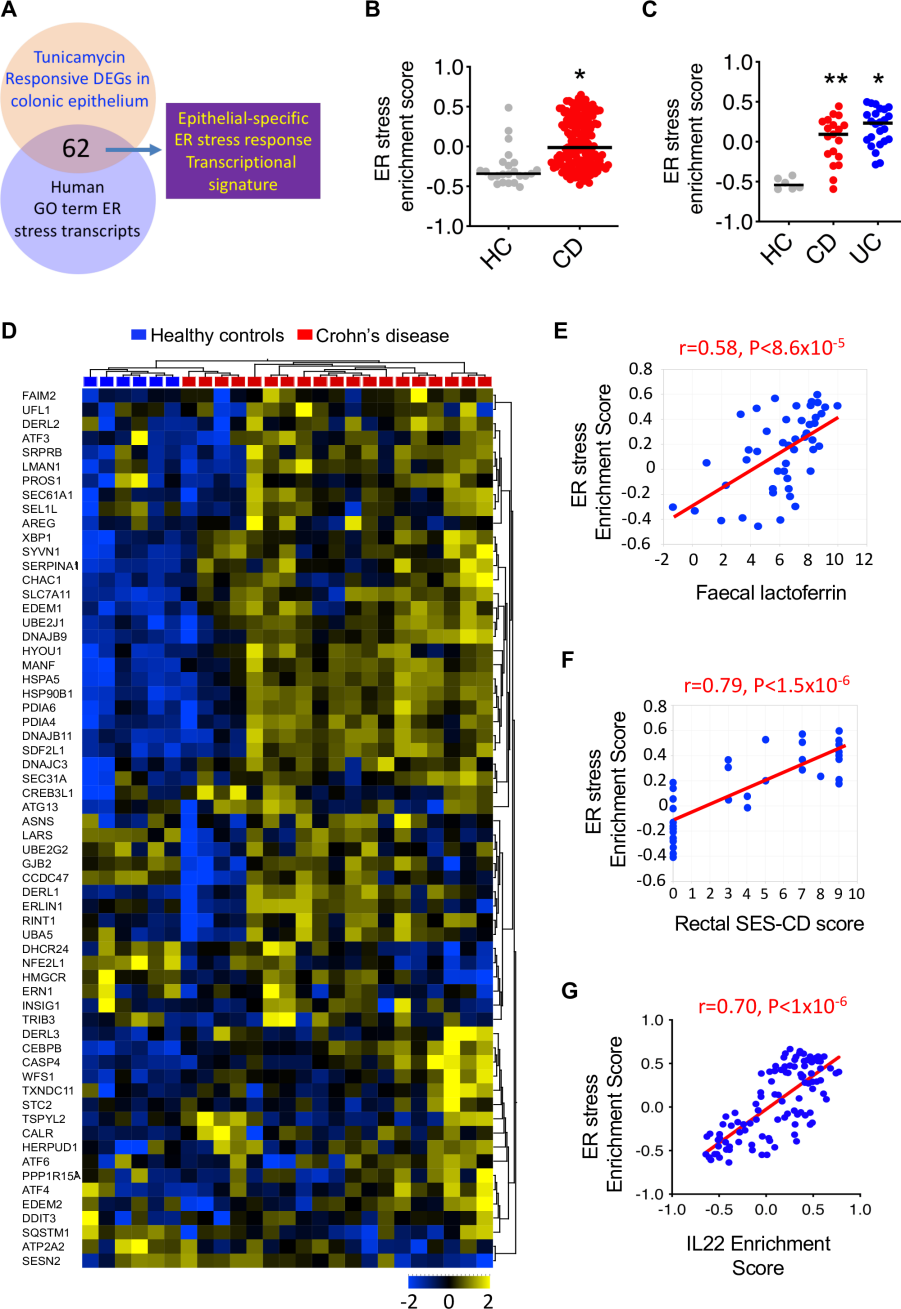
Colonic epithelial cell-specific ER stress response transcriptional module is enriched in IBD patients with active colitis. (A) Diagrammatic representation of how our colonic epithelial-specific ER stress response transcriptional signature was derived. (B) ER stress response GSVA enrichment scores in colonic biopsies from CD patients from the UNITI trial programme (at baseline prior to randomisation to placebo or ustekinumab) or healthy control subjects. Each dot represents an individual patient. line depicts median. *P<0.0001 (CD vs control). (C) ER stress response GSVA enrichment scores in colonic biopsies from UC, CD and healthy control (HC) patients from an independent dataset (GSE16879). *p<0.0005, **p<0.0001. (D) heat MAP depicting transcript level changes using unsupervised hierarchical clustering of the ER stress transcriptional module in patients with colonic CD and healthy, non-inflammatory control subjects (GEO59071). (E) Correlation between ER stress GSVA enrichment score and faecal lactoferrin concentration (log2) transformed data and (F) regional SES-CD. Displayed is baseline rectum gene expression from UNITI-1 subpopulation with colonic involvement. (G) Correlation between IL22 GSVA enrichment score and epithelial cell-specific ER stress GSVA enrichment score. CD, Crohn’s disease; DEGs, differentially expressed genes; ER, endoplasmic reticulum; GO, Gene Ontology; GSVA, Gene Set Variation Analysis; IL22, interleukin-22; SES-CD, Simple Endoscopic Score – Crohn’s Disease.

Next, we investigated whether the ER stress response transcriptional programme might also be enriched in the colon of patients with active colitis. To generate a colon epithelial cell-specific ER stress-associated transcriptional module, we identified the genes induced by tunicamycin in murine colonoids and cross referenced them with their human homologues involved in the ER stress pathway, generating a list of 62 genes (figure 6A). GSVA confirmed that this colonic epithelial cell-specific, ER stress response transcriptional module was significantly enriched in the colon of patients with colonic CD in the UNITI cohort (figure 6B) and replicated in an independent dataset of patients with colonic CD and UC (figure 6C). Unsupervised hierarchical clustering according to expression of these 62 ER stress response transcripts could fully differentiate between patients with colonic CD and non-inflammatory control patients (figure 6D), and significantly correlated with key disease features, including faecal lactoferrin concentration (figure 6E) and endoscopic severity of mucosal injury (figure 6F). In keeping with a role for IL22 in driving an ER stress transcriptional module in human colitis, the magnitude of enrichment of IL22 responsive transcripts in the colon across the population of patients with CD significantly correlated with the magnitude of enrichment of the ER stress transcriptional module (figure 6G).

### In vivo blockade of the IL23/IL22 axis reverses ER stress in Crohn’s colitis

**Figure 7:**
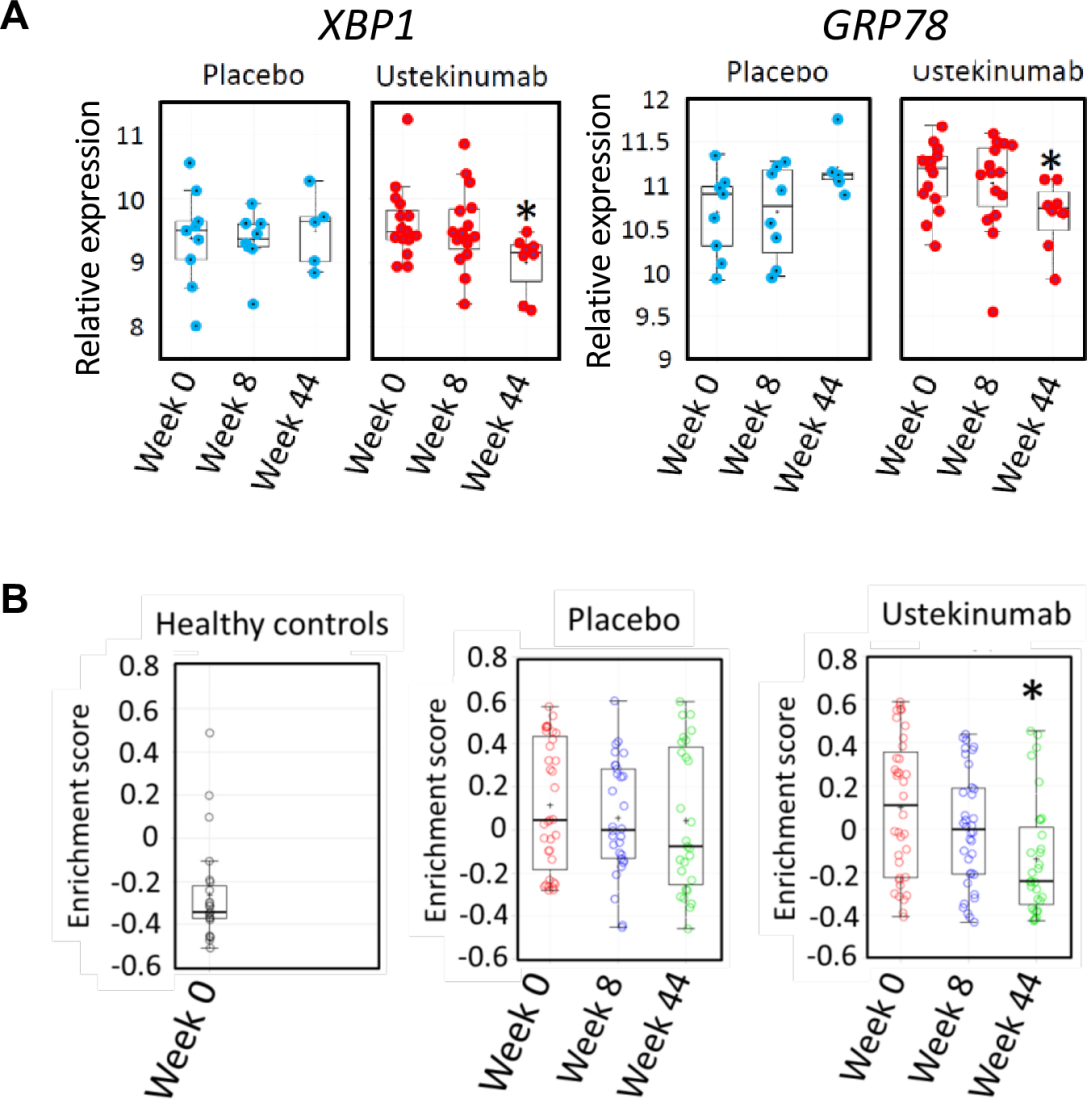
Blockade of the IL12/IL23 axis with ustekinumab alleviates colonic ER stress in CD patients with active colitis. (A) quantification of transcripts encoding *XBP1* and *GRP78* (log2 transformed expression intensity) in colonic biopsies of patients with CD in UNITI-1 randomised maintenance population post-treatment with maintenance placebo or maintenance ustekinumab (pooled data of ustekinumab 90 mg SC every 12 weeks and ustekinumab 90 mg SC every 8 weeks at week 44 comparing to week 0. (B) GSVA enrichment scores of ER stress signatures in the UNITI-2 pooled (randomised and non-randomised) maintenance population who received placebo or ustekinumab 90 mg every 8 weeks maintenance therapy until week 44 comparing with week 0). Gene expression data for the UNITI cohort were quantified using the Affymetrix Hg U133 PM array. CD, Crohn’s disease; ER, endoplasmic reticulum; IL22, interleukin-22; GSVA, Gene Set Variation Analysis.

We reasoned that if there was a causal relationship between IL22 and ER stress in human colonic inflammation, modulating the IL22 pathway might be expected to alleviate the ER stress transcriptional module in the colon. IL23 is the key cytokine responsible for triggering IL22 production; therefore, we tested the hypothesis that IL23 blockade would resolve colonic epithelial ER stress in human colitis. To address this question, we investigated the colonic transcriptome in the subset of patients from the UNITI trial programme treated with ustekinumab or placebo, in whom colonic biopsies had been serially sampled for RNA extraction at baseline, week 8 and week 44. Over time, in placebo-treated patients with CD, there was no significant change in the expression of *XBP1* or *GRP78* expression in the rectum. However, in patients treated with ustekinumab, there was a significant reduction in the expression of these transcripts by week 44 (figure 7A). Similarly, enrichment of the 62-transcript epithelial cell-specific transcriptional module remained unchanged in the colon of placebo-treated patients with CD, whereas it was significantly reduced in patients treated with ustekinumab (figure 7B). Taken together, these data are consistent with the IL23/IL22 axis playing an important role regulating colonic epithelial ER stress in IBD patients with colonic involvement.

## Discussion

This study provides new insights into the dialogue between IL22 and colonic epithelial cells in the context of chronic inflammation. IL22 regulated a transcriptional programme that shaped the inflammatory tone of the colonic epithelium, regulating transcripts including *Tnf*, inducible nitric oxide synthetase (*Nos2*) and pro-apoptotic factors, such as caspase 12 and Sting (*Tmem173*). Furthermore, IL22 also regulated an ER stress response module, which was amplified by IL17A, a cytokine it is commonly coproduced with.^19 20^ UPR induction is usually compensatory, to mitigate the harmful effects of misfolded proteins. However, persistence of triggering ER stressors, including chronic exposure to environmental insults and genetic factors, may result in unresolved ER stress, which in turn culminates in a pathogenic response and induction of apoptosis. These divergent cellular destinies following UPR engagement could potentially explain the contradictory, proinflammatory and anti-inflammatory roles attributed to IL22. For instance, IL22 is required for epithelial recovery in self-limiting colonic infections or following short-term exposure to chemicals that are directly toxic to intestinal epithelial cells.^2 4 6 7 18 34–36^ Induction of an adaptive UPR in this acute setting would likely be beneficial to overcome short-lived protein misfolding occurring during cellular stress to facilitate recovery. Even limited induction of apoptosis could be bioenergetically favourable in acute injury, allowing unsalvageable cells to be abandoned and priming the tissue for newly generated epithelial cells to replenish the barrier. Other functions of IL22, such as induction of epithelial stem cell proliferation to support epithelial repopulation would complement this activity. However, epithelial cell fate in response to IL22 induction of the UPR is likely to be very different in chronic inflammation, where persistent immune activation and excessive production of many different proinflammatory mediators occurs. Indeed, increased ER stress is observed in primary epithelial cells and colonic biopsies from patients with IBD.^37–39^ Crucially, sustained UPR activation triggers apoptosis, including an ER-specific apoptotic response mediated by ER anchored caspase 12.^15 21^ In keeping with a detrimental impact of persistent UPR engagement, chronic models of intestinal inflammation, including the TRUC model of IBD described in this study, blockade or genetic deletion of IL22 alleviates disease,^8–11^ consistent with a non-redundant proinflammatory role for IL22.

In this study, IL22 induced increased expression of caspase 12 in colonic epithelial cells, which is recognised as a crucial step in the transition of a sustained UPR to induction of apoptosis.^21 22^ Similarly, IL22 induced expression of the pro-apoptotic mediator STING, which plays an important role in triggering type I interferon induction and increased small intestinal epithelial apoptosis.^11^ The pathological impact of IL22 on small intestinal epithelium is directly influenced by the inflammatory tone of the epithelium and disruption of autophagy, which promotes proinflammatory activity significantly amplifies pathological ER stress.^11^ The role of IL22 in the regulation of epithelial cell survival is complex, and it is likely that different epithelial cell lineages are differentially impacted by both ER stress induction and susceptibility to apoptosis. Although definitive identification of which lineages are impacted was beyond the remit of the current study, we observed induction of an ER stress response in both LGR5^+^ epithelial cells as well as the LGR5^−^ epithelial compartment. Recently published data using small intestinal enteroids indicates that IL22 induces proliferation of transit-amplifying epithelial cells but simultaneously induces apoptosis of LGR5^+^ stem cells through inhibition of Wnt and notch signalling.^40^ Future work, including single cell sequencing experiments, will focus on defining the differential impact of different cytokines in different colonic epithelial lineages.

For the first time we show that IL22 performs a non-redundant, pathogenic role in the TRUC model of chronic colitis. Genetic ablation or antibody blockade of IL22 reversed the ER stress response and attenuated disease, although disease could be reinstated in TRUC *Il22^−/−^* mice by local pharmacological induction of ER stress in the colon. Pharmacological alleviation of ER stress attenuated TRUC disease. Taken together these data support a pathogenic role for IL22-induced colonic epithelial ER stress in chronic colitis. IL22 significantly amplified ER stress induced by tunicamycin, and just as *Atg16l1* deficiency promotes IL22 induced ER stress in the small intestine, it is likely that genetic and/or environmental variables in TRUC disease may potentiate IL22-induced ER stress and pathology. IL17A, which synergises with IL22 to induce colonic epithelial ER stress, is highly produced in the colon of TRUC mice and is functionally important in disease.^19 20^ TRUC disease is characterised by intestinal dysbiosis, including expansion of proinflammatory bacteria, such as *Helicobacter typhlonius*. Intriguingly, related *Helicobacter* species, including *Helicobacter hepaticus*, have been shown to induce ER stress in intestinal epithelial cells.^41 42^

The molecular mechanisms of IL22 and IL17A engagement with the UPR in colonic epithelial cells has not been formally tested in this study, although a number of possibilities exist. IL22 canonically signals through STAT3,^43^ and in the small intestine, IL22-mediated induction of the UPR is STAT3 dependent,^11^ indicating that ER stress programme could be directly transcriptionally regulated through STAT3 activation. IL22 also signals through MAP kinase pathways, such as MAP3K8,^44^ which might represent a more plausible mechanism of convergence with IL17A signalling. Our transcriptomic data hinted at alternative mechanisms. IL22 induced *Nos2* (inducible nitric oxide synthetase), which as well as mediating DNA damage and colonic epithelial carcinogenesis,^23^ is both a trigger and downstream effector of the ER stress response.^24^ IL22 also upregulated *Tlr4* and its signalling adapter *Myd88* in colonoids, and engagement of this microbial recognition pathway also induces ER stress in colonic epithelial stem cells.^45^ Therefore, it is possible that IL22-induced regulation of TLR4 signalling could sensitise to ER stress induction, which might be especially pertinent in vivo in the setting of disease processes that microbial dependent, such as IBD. Future work will investigate the functional importance of these possible mechanisms of engagement.

A major strength of this study is our analysis of colonic transcriptomic data from large cohorts of patients with active colitis, including colonic CD patients treated with ustekinumab or placebo in the context of a large phase III clinical trial. In different patient cohorts, the IL22-induced transcriptional module was highly enriched in the colon of patients with active colonic CD and UC, correlating with disease biomarkers and endoscopic severity of disease. Moreover, the expression of IL22 responsive transcripts strongly correlated with the ER stress response transcriptional module in the colon of patients with active CD.

In conclusion, data presented in this study advance our understanding of IL22 biology in the colon and cast new light on pathogenic mechanisms of this important cytokine in chronic colitis. The IL22/ER stress axis may be especially important in chronic inflammation, where other proinflammatory/proapoptotic mediators, such as IL17A and TNFα, are also excessively and persistently produced. Therapeutic strategies targeting ER stress or neutralising effector cytokines responsible for driving pathological ER stress responses in the colon are conceptually attractive therapeutic approaches for IBD patients with active colitis. Likewise, blockade of upstream cytokines, such as IL23, may be especially effective in patients with pronounced epithelial ER stress. The role of IL22 in IBD may need reinterpretation and the rationale for exogenous IL22 supplementation in patients with active colitis may need re-evaluation.
