# Supplementary material for: Interleukin-22 orchestrates a pathological endoplasmic reticulum stress response transcriptional programme in colonic epithelial cells
Source: Gut. 2019 Dec 2;69(3):578–90. doi: 10.1136/gutjnl-2019-318483 (PMC7034350; doi:10.1136/gutjnl-2019-318483)
Supplement: Supplementary data [file gutjnl-2019-318483supp001.pdf]

Supplementary methods

Mice

Balb/C *Tbx21*$^{-/-}$ *Rag2*$^{-/-}$ double knockout (TRUC) mice have been described previously[1, 2]. Balb/C *Il22*$^{-/-}$ were provided by Pfizer and backcrossed with Balb/C *Tbx21*$^{-/-}$ *Rag2*$^{-/-}$ and *Rag2*$^{-/-}$ to generate *Tbx21*$^{-/-}$ *Rag2*$^{-/-}$ *Il22*$^{-/-}$ triple knockout mice and *Rag2*$^{-/-}$ *Il22*$^{-/-}$ double knockout mice, respectively. Lgr5 GFP reporter mice were provided by Professor Fiona Watt, King's College London. All animal experiments were conducted in accredited facilities in accordance with the UK Animals (Scientific Procedures) Act 1986 (Home Office license number PPL 70/6792 and 70/7869).

In vivo treatment

Neutralizing anti-IL22 mAb (clone IL22-01) and recombinant IL22 (rIL22) were developed and provided by Pfizer. IL22-01 (200µg per mouse) was administered every 3-4 days. Tunicamycin (1µg per mouse at days 0 and 5) was administered intra-rectally to TRUC *Il22*$^{-/-}$ mice. 4-phenylbutyric acid (4PBA) was administered in drinking water (20mM) *ad libitum* for 6 weeks to TRUC mice. ILC depletion was achieved by administering anti-CD90.2 (clone 30H12, Bio X Cell) once weekly for 4 weeks. *In vivo* treatments were conducted in age matched animals aged 8-12 weeks old. No formal power calculations or exclusions were conducted. End points, including weight loss, colon mass, spleen mass and colitis scores were predefined.

cLPMC isolation

Mice were euthanized by cervical dislocation and colons were removed and placed in ice-cold PBS. Colons were opened longitudinally, cleaned thoroughly with ice-cold PBS and cut into 1-2mm pieces and washed in HBSS (5mM EDTA, 1mM Hepes) in a shaking water bath at 37°C for 20min. Tissue was then vortexed vigorously for 10sec and passed through a 100µM cell strainer and collected in C-tubes (Miltenyi) in complete RPMI (Gibco) containing 10% fetal calf serum, 0.25mg/ml Collagenase D (Roche), 1.5mg/ml Dispase II (Roche) and 0.01ug/ml DNase (Roche) and put in a shaking water bath (300rpm) at 37°C for 40min. Before and after the 40min incubation C-tubes were vigorously shaken for 30sec. Solutions were then passed

through 100μM cell strainers and washed with ice-cold PBS. Cells were resuspended in 10ml of the 40% fraction of a 40:80 Percoll (GE Healthcare) gradient and carefully placed on top of 5ml of the 80% fraction in 15ml tubes. Percoll gradient separation was performed by 20min centrifugation at 2600rpm at room temperature. LP cells were collected from the interphase of the gradient and washed with ice-cold PBS. Cells were resuspended in 1ml PBS, counted and immediately used for further experiments.

Colonoid culture

Mouse colonic crypts were isolated, cultured, and grown into organoids following the methods of Sato *et al.*[3]. Crypts were cultured in growth medium containing advanced Dulbecco's modified Eagle's medium/F12, penicillin/streptomycin (100 units/mL), 10 mM HEPES, 2 mM Glutamax, supplements N2 (1x) and B27 (1x), 50 ng/mL mouse epidermal growth factor (all from Life Technologies), 1 mM N-acetylcysteine (Sigma-Aldrich), 50% v/v Wnt3a conditioned medium, 10% v/v R-spondin-1 conditioned medium, 10% v/v Noggin conditioned medium and 3 μM CHIR99021 (Cambridge Biosciences). Medium was changed every 2 days. Differentiation towards a mature epithelium was achieved by withdrawal of Wnt3a for 3 days. During the last 24h in differentiation medium colonoids were treated with recombinant IL22 (10ng/mL), IL17A (50ng/mL) or TNFα (5-20ng/mL) respectively. For ILC/colonoid co-culture experiments, colonic NCR$^-$ ILC3 *Il22*$^{+/+}$ or NCR$^-$ ILC3 *Il22*$^{-/-}$ were FACS purified (CD45$^+$, IL7R$^+$, NKp46$^-$, KLRG1$^-$) and were cultured for 48 hours with IL2 (20ng/mL), IL7 (50ng/mL), IL23 (10ng/mL) and IL1β (10ng/mL). Activated NCR$^-$ ILC3 were resuspended in differentiation medium and co-cultured with colonoids at an ILC/crypt ratio of 25:1.

Gene expression studies

For the Illumina gene expression arrays (TRUC and *Rag2*$^{-/-}$ colon, and colonic ILCs) RNA was extracted using Trizol reagent (Invitrogen, UK). cDNA was synthetized using Ovation PicoSL WTA System V2 and labelled using Encore BiotinIL module (NuGen, USA). RNA and cDNA quantity and quality were assessed using the Agilent RNA 6000 Nano Kit and Agilent RNA 6000 Pico Kit according to the manufacture's protocol (Agilent Technologies, USA). Labelled

cDNA were hybridised on a MouseWG-6 v2.0 Expression BeadChip (Illumina, USA). For Affymetrix expression arrays (colonoid experiments) SPIA cDNA was generated from 25ng total RNA using the "Ovation Pico WTA System V2" kit (NuGEN), following the manufacturer's instructions. The SPIA cDNA was subjected to a QC check to assess quality (Agilent 2100 Bioanalyzer) and quantity (Nanodrop ND-1000 Spectrophotometer) for the next stage. The SPIA cDNA was fragmented and Biotin-labelled using the "Encore Biotin Module" from NuGEN according to the manufacturer's instructions. The fragmented and Biotin-labelled cDNA was subjected to a further round of QC checks to assess fragmentation size (Agilent 2100 Bioanalyzer). Hybridisation cocktails were prepared from the fragmented labelled-cDNA according to NuGEN's recommendations and hybridised to Mouse Gene 2.0 ST arrays at 45˚C overnight in the 645 Hybridisation oven (Affymetrix). The arrays were washed and stained using wash protocol FS450_0002 in the GeneChip Fluidics station 450. The arrays were scanned using the Affymetrix GeneChip Scanner GCS3000 7G. Gene expression analysis was performed using Partek Genomics Suite software, version 6.6 (St. Louis, MO), Ingenuity Pathway Analysis (Qiagen) and functional-annotation enrichment analysis for Gene Ontology biological processes, using the Database for Annotation, Visualization and Integrated Discover (DAVID)[39,40]. To explore the relevance of IL22 responsive genes (identified by mouse organoid models) in the context of human disease we interrogated one of the largest datasets of mucosal gene expression profiling (Affymetrix GeneChip Human Gene 1.0 ST arrays) in patients with ulcerative colitis (UC) and controls reposited at the Gene Expression Omnibus (GSE59071) [41]. Data was analyzed by Partek Genomics Suite software, version 6.6 (St. Louis, MO). After data importation multiple comparisons in gene expression between groups (controls, active and quiescent UC) were performed using ANOVA. GraphPad Prism version 7 (La Jolla California USA), was used to estimate correlation coefficients between genes of interest and draw graphs. The data shown were normalized to the mean expression of the gene of interest in the control group.

For mRNA sequencing (RNA-seq), distal colon segments from preclinical models of colitis (and controls) were immediately immersed in RNAlater and incubated at 4°C overnight before

storing at -20°C. RNA was extracted (Trizol) with QC check to assess quality (Agilent 2100 Bioanalyzer) and quantity (Nanodrop ND-1000 Spectrophotometer). mRNA was enriched using oligo(dT) beads and then fragmented randomly in fragmentation buffer, followed by cDNA synthesis using random hexamers and reverse transcriptase. After first-strand synthesis, a custom second-strand synthesis buffer (Illumina) is added with dNTPs, RNase H and Escherichia coli polymerase I to generate the second strand by nick-translation. The final cDNA library is ready after a round of purification, terminal repair, A-tailing, ligation of sequencing adapters, size selection and PCR enrichment. Library concentration was first quantified using a Qubit 2.0 fluorometer (Life Technologies), and then diluted to 1ng/µl before checking insert size on an Agilent 2100 and quantifying to greater accuracy by quantitative PCR (Q-PCR) (library activity >2 nM). Sequencing was performed using the Illumina HiSeq platform with paired-end 150 bp sequencing strategy. The quality of the raw library files was inspected with fastQC[4]. Raw reads were trimmed and filtered to remove adaptor contamination and poor-quality bases using trimmomatic[5] with parameters: "LEADING:3 TRAILING:3 SLIDINGWINDOW:4:15 MINLEN:36 ILLUMINACLIP:$ADPT_FA:2:30:10:8:true" - where $ADPT_FA links to the fasta file with the Illumina adaptor sequences. The resulting read files were mapped against the GRCm38 assembly of the mouse genome using Hisat2[6] with default parameters. The number of reads mapping to the genomic features annotated in Ensembl[7] with a MAPQ score higher than or equal to 10 was calculated for all samples using htseq-count with default parameters. Differential expression analysis between the disease and control samples was performed in R with the DESeq2[8] package. P-values were adjusted for multiple testing according to the Benjamini and Hochberg procedure. Gene Set Enrichment Analysis was carried out with the clusterProfiler[9] package.

Quantitative PCR

Total RNA was extracted from cultured organoids using RNeasy mini kit and RNase-free DNase set (both from Qiagen) and reverse transcribed using a high-capacity cDNA reverse transcription kit (Life Technologies). Quantitative PCR was performed using QuantiTect

primers and Quantitect SybrGreen MasterMix (both from Qiagen) on a LightCycler 480 (Roche). Sequences for mouse and human XBP1 splicing primers sequences were obtained from Kaser *et al.*[30]. Samples were analyzed in triplicates and relative expression of mRNAs was determined after normalisation against the housekeeping gene Beta-2-Microglobulin (B2M).

Western blotting

Colonic tissues were homogenized in RIPA buffer prior to western blotting. Protein lysates were fractionated in 4-15% SDS-PAGE and transferred onto nitrocellulose membranes. Grp78 expression was measured using a purified anti-Grp78 (C50B12) rabbit monoclonal antibody (#3177, Cell Signalling Technology at 1:500). β-actin (#4967, Cell Signalling Technology at 1:1000) was used as a loading control. Reactivity was determined using an HRP-conjugated secondary antibody (#NA934V, GE Healthcare UK Limited at 1:10,000) and revealed by enhanced chemiluminescence with standard film cassettes and film developing equipment. Autoradiography film were scanned with a G:BOX Chemi XRQ gel doc (Syngene) using GeneSnap software and densitometry performed using GeneTools software (Syngene). Specificity of immunblotting was confirmed by demonstrating loss of mass specific bands after incubating with Bip blocking peptide (#1084, Cell Signalling Technology).

IRE1α Immunohistochemistry

Formalin-fixed paraffin-embedded sections were deparaffinized in xylol and rehydrated in ethanol. Antigen retrieval was performed in citrate buffer at subboiling temperature, followed by endogenous peroxidase activity quenching. Sections were incubated overnight with IRE1α antibody (Santa Cruz Biotechnology, sc-20790 (H-190)) and HRP/DAB (ABC) Detection IHC kit (Abcam, ab64261) was used for detection. Sections were analyzed using Axio Observer Z.1 microscope and AxioCam MRc5 camera, using Apochromat lenses and Zen Blue 2012 software.

In vivo apoptosis assay

For the *in vivo* apoptosis assay, male C57BL/6 mice, aged 10-12 weeks were housed and maintained in SPF conditions at the University of East Anglia, Norwich, UK. Experimental animals were closely monitored and were sacrificed by rising CO_2 and cervical dislocation, at the time points described in the text. Transient, acute inflammation was induced by single intraperitoneal injection of recombinant murine TNFα (Peprotech, London, UK) at 0.5 mg/kg. Cytokine pre-treatment involved intraperitoneal delivery of IL22 (2.5μg/mouse) or combined IL22 + IL17A (2.5μg/1.5μg respectively) 6 h prior to TNFα delivery. At 40, 60, 90, 120 and 150 minutes post-TNFα administration, mice were euthanized and intestinal tracts were removed, dissected, formalin-fixed and paraffin embedded. Transverse sections of duodenum and ileum were prepared at 5μm and were immunostained for cleaved caspase 3 using anti-CC3 (R&D Systems, Minneapolis, USA) and diaminobenzidine reaction (DAB, Dako, Glostrup, Denmark), sections were counterstained with H&E. Caspase-positive cells were recorded for 30-50 individual hemi crypt-villus units per tissue section per mouse, with counts recorded as binary values. An ANOVA analysis followed by a Tukey test were performed on the logarithmic transformation of the quantities of caspase-positive cells using SAS 9.4.

Murine histology

Colon histology was prepared and scored in a blinded fashion as described previously[2]. In short 1cm segments of colon were fixed in 10% paraformaldehyde and embedded in paraffin blocks. 5μm sections were stained with haematoxylin and eosin (H&E).

UNITI trial program

The UNITI trial program comprised 2 parallel, randomized placebo-controlled phase 3 clinical trials evaluating the efficacy and safety of Ustekinumab (NCT01369342 and NCT01369329) and has already been reported[10]. In this study, for the first time we report transcriptional data

from biopsies, which was correlated to clinical, endoscopic and biomarker data available from the UNITI cohort. Colonic biopsies were sampled at defined time points after institution of treatment in a subset of patients and were immediately transferred to RNALater (Qiagen) and stored at -80°C prior to RNA extraction. Whole genome transcriptomics were performed on the Affymetrix HG U133 PM array. Clinical data was recorded prospectively according to the clinical protocol[10].

Statistics

Two-tailed Mann-Whitney U tests were carried out using Graphpad Prism. Elsewhere mean values are expressed with error bars denoting standard error of the mean (SEM).

Supplementary methods references

1. Powell N, Lo JW, Biancheri P, et al. Interleukin 6 Increases Production of Cytokines by Colonic Innate Lymphoid Cells in Mice and Patients With Chronic Intestinal Inflammation. Gastroenterology 2015;149:456-67.e15.
2. Powell N, Walker AW, Stolarczyk E, et al. The transcription factor T-bet regulates intestinal inflammation mediated by interleukin-7 receptor+ innate lymphoid cells. Immunity 2012;37:674-84.
3. Sato T, Stange DE, Ferrante M, et al. Long-term expansion of epithelial organoids from human colon, adenoma, adenocarcinoma, and Barrett's epithelium. Gastroenterology 2011;141:1762-72.
4. Andrews S. FastQC A Quality Control tool for High Throughput Sequence Data. http://www.bioinformatics.babraham.ac.uk/projects/fastqc/.
5. Bolger AM, Lohse M, Usadel B. Trimmomatic: a flexible trimmer for Illumina sequence data. Bioinformatics 2014;30:2114-20.
6. Kim D, Langmead B, Salzberg SL. HISAT: a fast spliced aligner with low memory requirements. Nat Methods 2015;12:357-60.
7. Zerbino DR, Achuthan P, Akanni W, et al. Ensembl 2018. Nucleic Acids Res 2018;46:D754-D761.
8. Love MI, Huber W, Anders S. Moderated estimation of fold change and dispersion for RNA-seq data with DESeq2. Genome Biol 2014;15:550.
9. Yu G, Wang LG, Han Y, et al. clusterProfiler: an R package for comparing biological themes among gene clusters. OMICS 2012;16:284-7.
10. Feagan BG, Sandborn WJ, Gasink C, et al. Ustekinumab as Induction and Maintenance Therapy for Crohn's Disease. N Engl J Med 2016;375:1946-1960.
