# Supplementary material for: Interleukin-22 orchestrates a pathological endoplasmic reticulum stress response transcriptional programme in colonic epithelial cells
Source: Gut. 2019 Dec 2;69(3):578–90. doi: 10.1136/gutjnl-2019-318483 (PMC7034350; doi:10.1136/gutjnl-2019-318483)
Supplement: Supplementary data [file gutjnl-2019-318483supp002.pdf]

Supplementary Figure 1

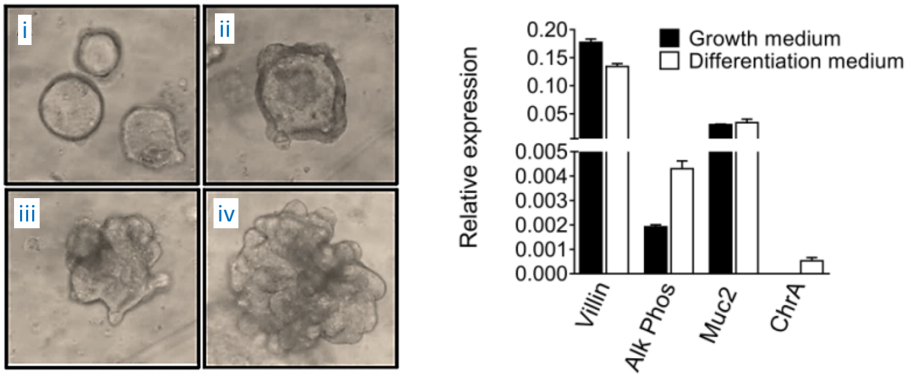

Supplementary Figure 1: Generation of murine colonoids as a tool to interrogate immune-epithelial interactions. *Left panel* shows 3D colonic epithelial "miniguts" were grown from isolated mouse colonic crypt units. Images depict development of organoids over time. **i**,3 days, **ii**, 4 days, **iii**, 5 days **iv**,6 days post isolation/passage. *Right panel* - Expression of epithelial markers Villin (all epithelial cells), Alkaline phosphatase (mature enterocytes), Mucin (goblet cells), and chromogranin A (endocrine cells) in colonoids cultured in growth and differentiation medium.

Supplementary Figure 2

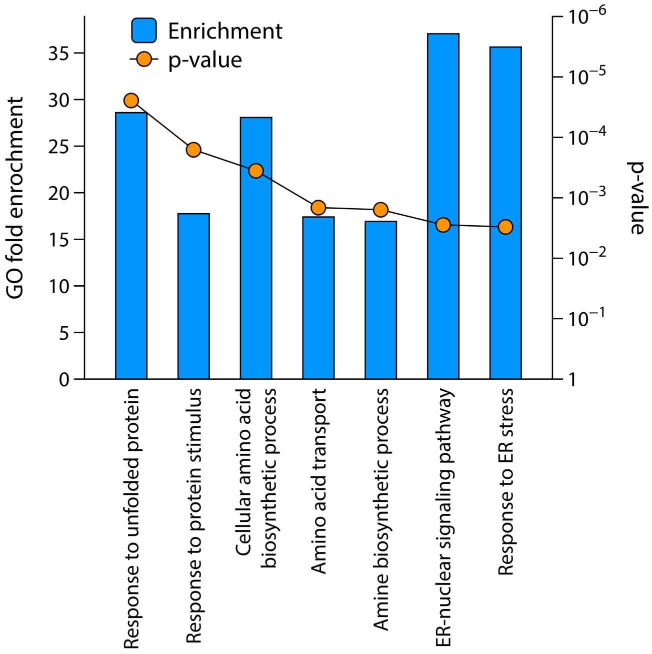

Supplementary Figure 2: Tunicamycin induces an ER stress response transcriptional module in colonic epithelial cells. Enrichment analysis for functional annotation groups (GO biological processes) of 163 genes that were significantly ($P<0.05$) differentially expressed (-/+2 fold change) by colonoids after treatment with tunicamycin (n=3) compared to vehicle treated colonoids (n=3, Mouse Gene 2.0 ST array, Affymetrix.

Supplementary Figure 3

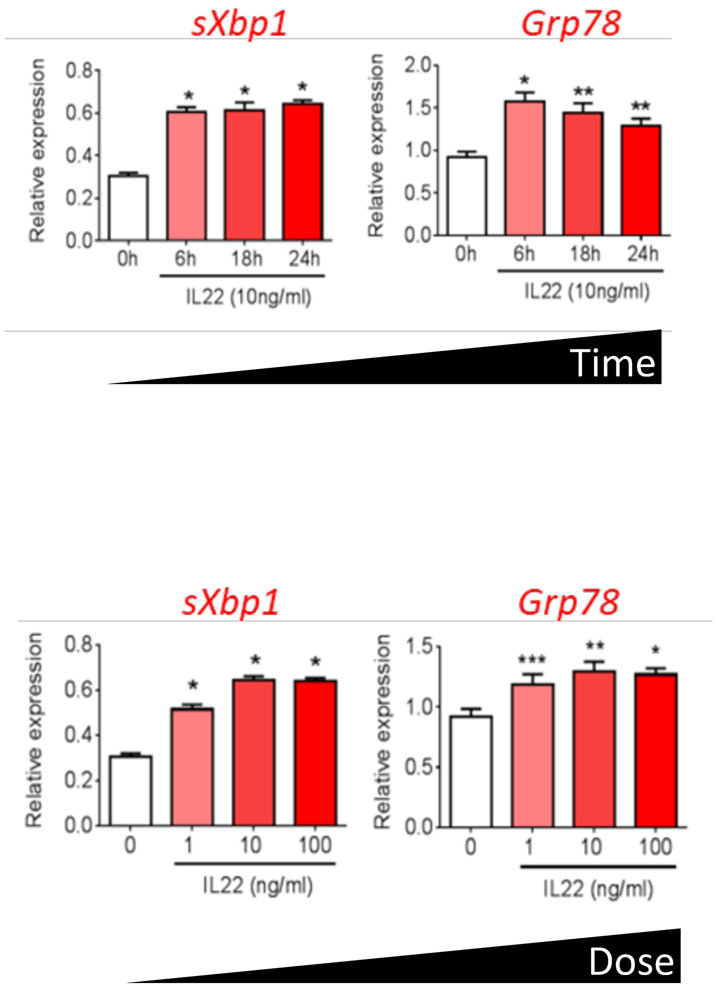

Supplementary Figure 3: IL22 induces an ER stress response transcripts in a time and dose dependent manner. Real time qPCR analysis of ER stress transcript expression in colonoids exposed to IL22 for varying amounts of time, and different doses of IL22 (f, n=6 for each dose or time point). Bars show mean expression and error bars depict SEM. *P<0.005, **P<0.02, ***P<0.05 (Mann Whitney U Test).

Supplementary Figure 4

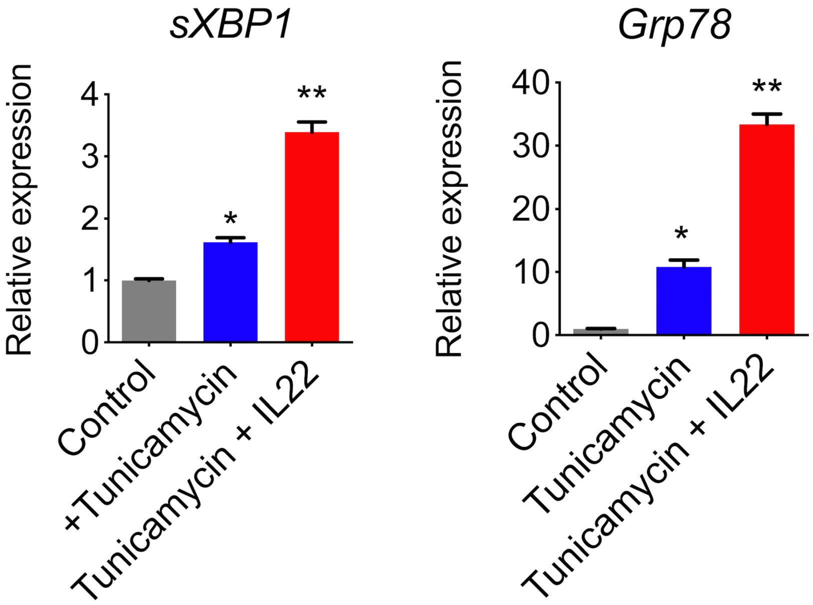

Supplementary Figure 4: IL22 synergistically augments ER stress induced by tunicamycin in colonoids. Real time qPCR analysis of ER stress transcript expression in colonoids exposed to tunicamycin in the presence or absence of IL22 (n=6 for condition). Bars show mean expression and error bars depict SEM. *P<0.0025 (vs control), **P<0.0025 (vs tunicamycin alone). Mann Whitney U Test.

Supplementary Figure 5

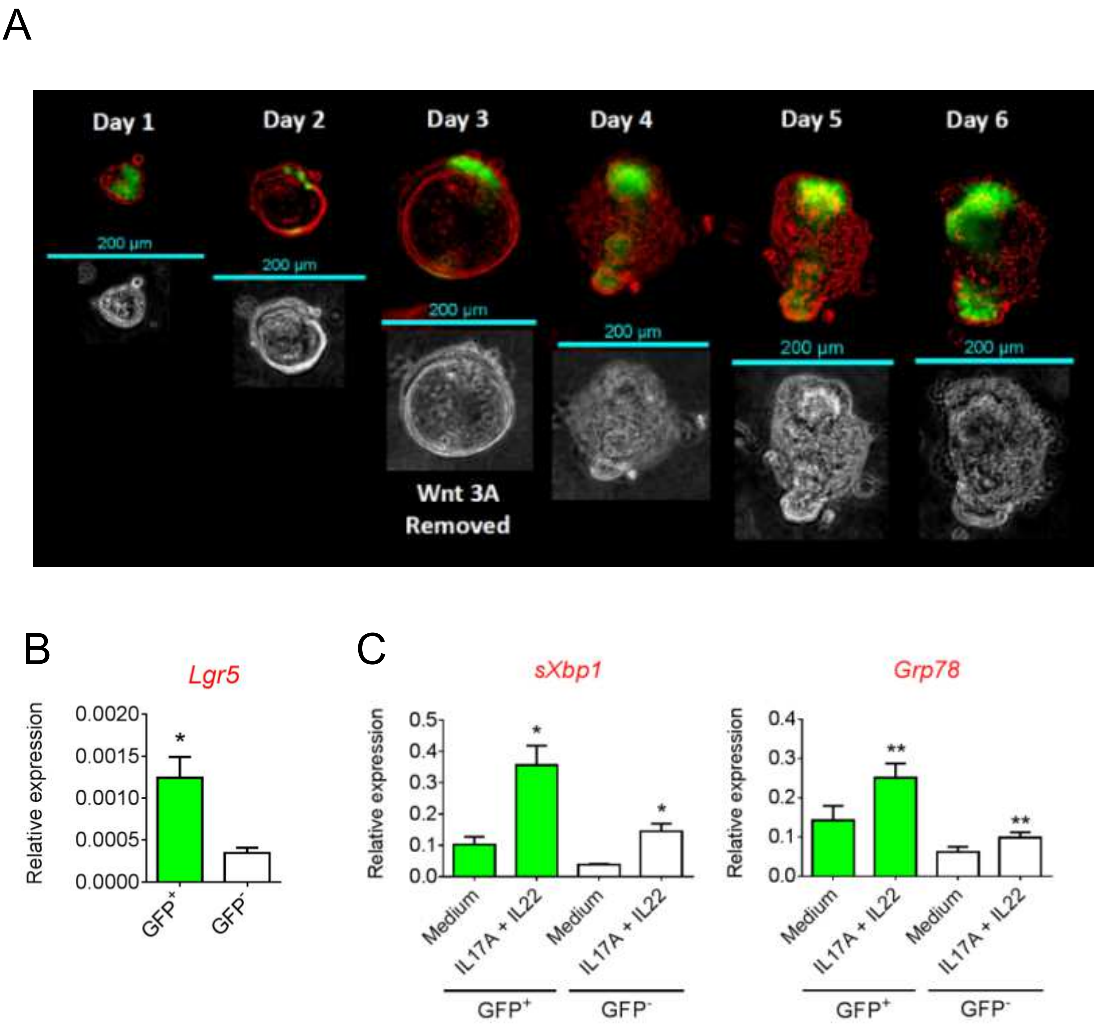

Supplementary Figure 5: Generation of LGR5-eGFP colonoids. (A) Microscopy of colonoids generated from Lgr5 GFP mice at different days of development. (B) Real time PCR analysis of *Lgr5* mRNA expression in FACs sorted GFP^+ and GFP^- $EpCam^+$ epithelial cells from colonoids (n=6) from Lgr5-GFP reporter mice. (C) Real time PCR analysis of *sXbp1* and *Gpr78* mRNA expression in FACs sorted GFP^+ and GFP^- $EpCam^+$ epithelial cells from colonoids (n=6) from Lgr5-GFP reporter mice. *P<0.005, **P<0.05.

Supplementary Figure 6

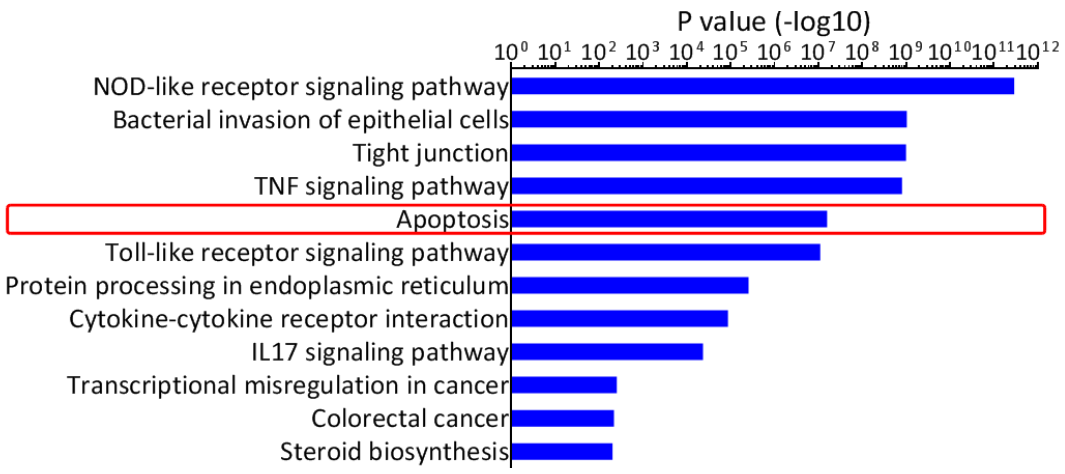

Supplementary Figure 6: KEGG pathway analysis of differentially expressed genes in defined signalling pathways in IL22 treated colonoids from ERR247358-ERR247389.

Supplementary Figure 7

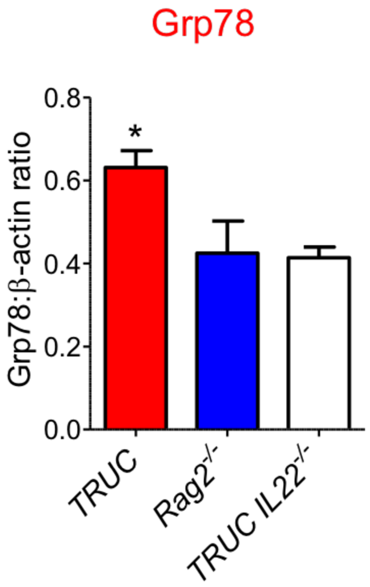

Supplementary Figure 7: Densitometry plot of Grp78 protein expression in Western blot from colon of TRUC mice. Western blots of Grp78 protein expression in the colon of TRUC (n=9) *Rag2*$^{-/-}$ (n=4) and TRUC *Il22*$^{-/-}$ mice (n=5). *P<0.01 (vs *Rag2*$^{-/-}$ and TRUC *Il22*$^{-/-}$ *).*

Supplementary Figure 8

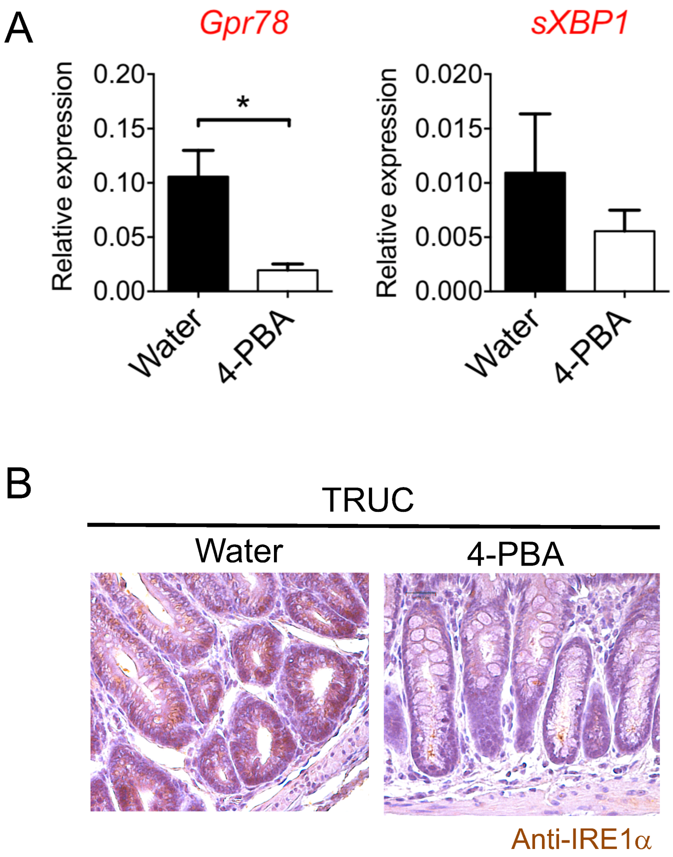

Supplementary figure 8: 4-PBA reduces colonic ER stress in TRUC mice. (A) Real time PCR analyses of ER stress transcript expression in the colon of the distal colon of TRUC mice treated with 4-PBA in drinking water (n=8) or water alone (n=8). *P<0.025, and representative immunohistochemistry (IRE1α immunoreactivity).

Supplementary Figure 9

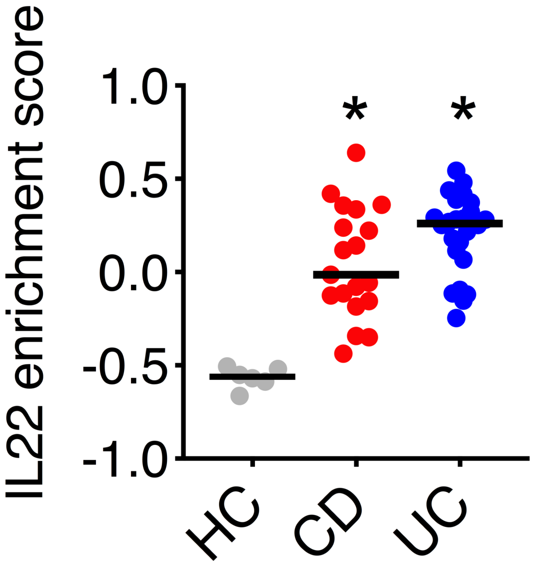

Supplementary figure 9: IL22 responsive transcripts are enriched in colonic biopsies sampled from patients with colonic CD and UC. Enrichment scores of IL22 responsive transcripts (GSVA) in healthy controls (n=6) and patients with colonic CD (n=19) and UC (n=24) from GSE16879. *P<0.0001.

Supplementary Figure 10

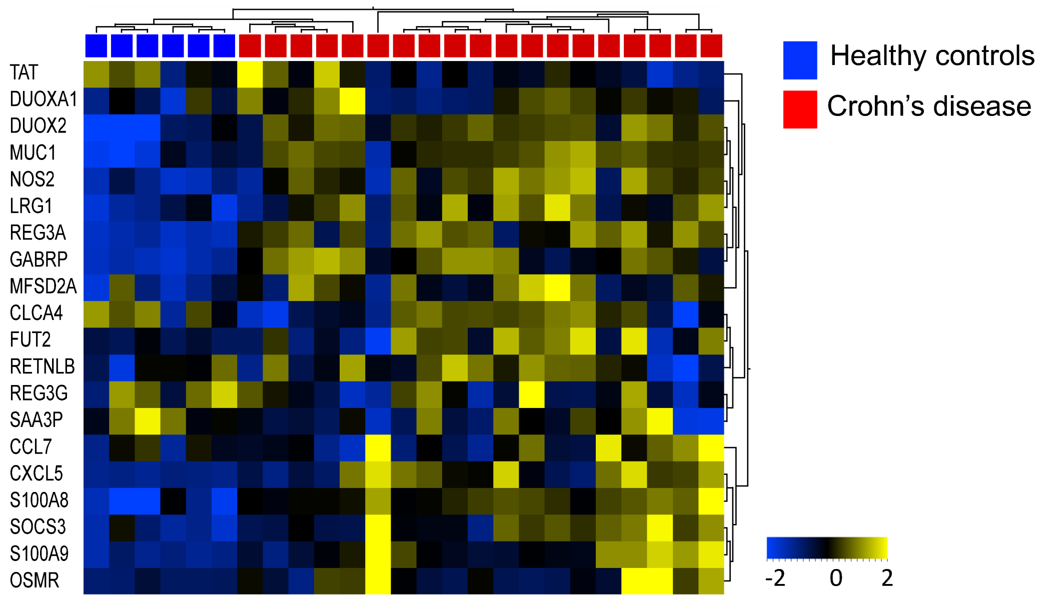

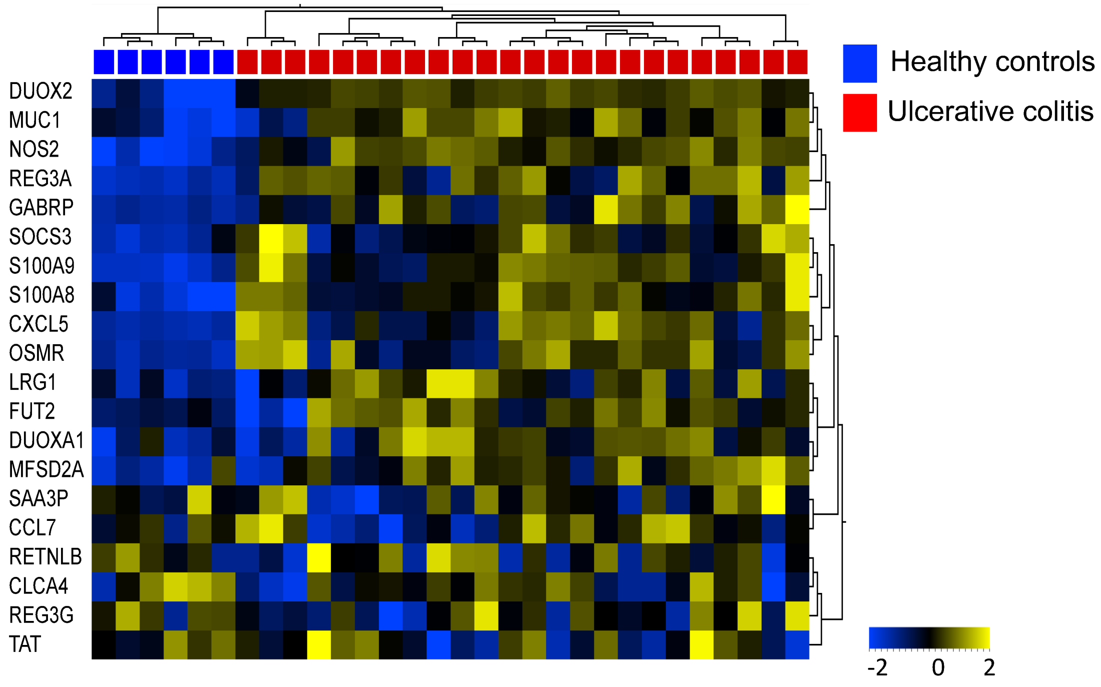

Supplementary Figure 10: Enrichment of IL22 responsive transcripts in the colon of IBD patients. Unsupervised hierarchical clustering of IL22 responsive transcripts in patients with CD (upper panel), UC (lower panel) and non-inflammatory control subjects (GSE16879).

Supplementary Figure 11

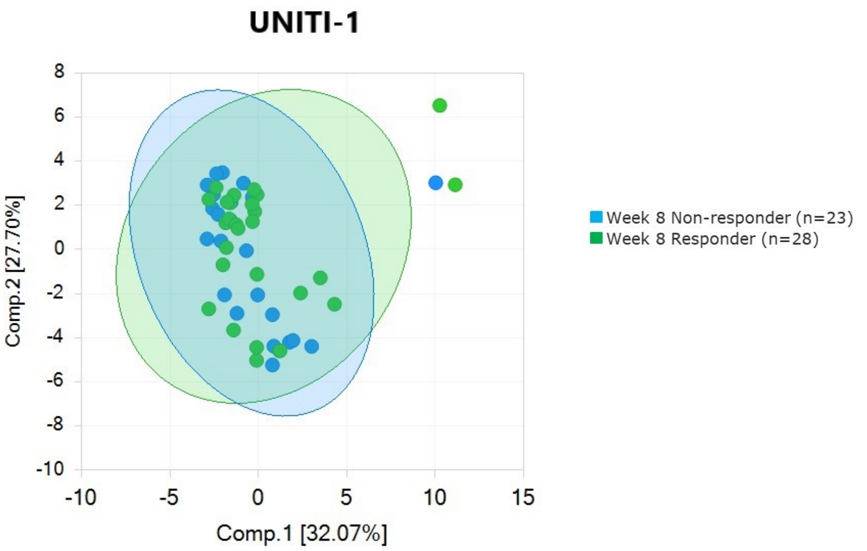

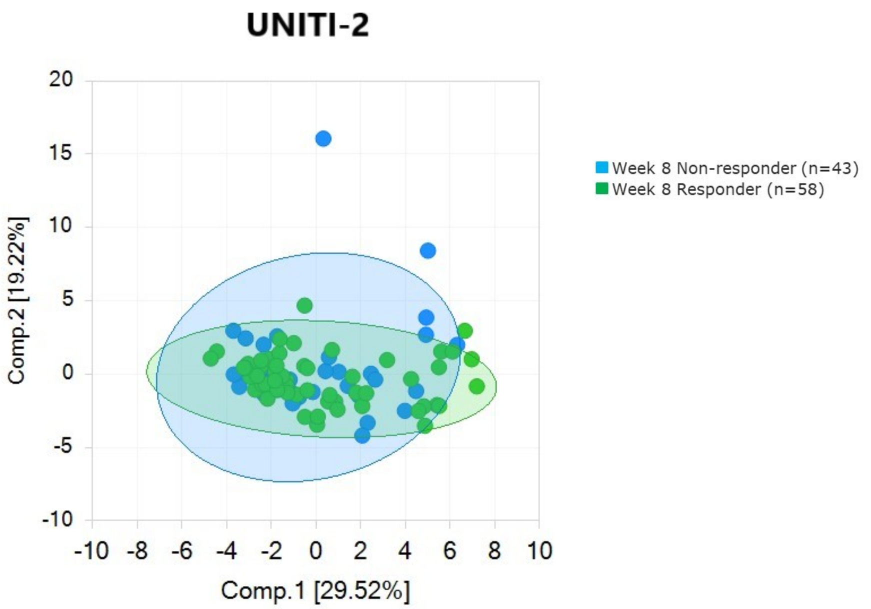

Supplementary Figure 11: IL22 responsive transcriptional signature does not predict response to ustekinumab in the UNITI trial program. Clinical response was measured by CDAI change (≥100 points) at week 8 (relative to baseline).
