# Supplementary material for: Interleukin-22 orchestrates a pathological endoplasmic reticulum stress response transcriptional programme in colonic epithelial cells
Source: Gut. 2019 Dec 2;69(3):578–90. doi: 10.1136/gutjnl-2019-318483 (PMC7034350; doi:10.1136/gutjnl-2019-318483)
Supplement: Supplementary data [file gutjnl-2019-318483supp003.pdf]

Supplementary Table 1

Transcript Cluster id	Gene	Fold change	p-value	q-value
17459744	Reg3b	187.07	7.21E-15	1.22E-10
17467973	Reg3g	90.08	9.77E-13	4.13E-09
17399823	S100a8	32.84	3.18E-13	2.69E-09
17346150	Lrg1	26.64	6.51E-13	3.67E-09
17254047	Ccl7	10.17	4.65E-11	6.55E-08
17438955	Cxcl5	9.18	2.00E-12	5.63E-09
17326062	Retnlb	7.36	4.76E-10	2.78E-07
17254289	Gm11428	7.28	2.48E-09	8.91E-07
17407363	S100a9	7.24	3.15E-10	2.22E-07
17505478	Tat	7.12	3.72E-11	5.72E-08
17315743	Osmr	6.19	2.29E-09	8.43E-07
17490912	Fut2	6.02	1.92E-12	5.63E-09
17491193	Saa3	5.93	7.39E-07	6.95E-05
17318089	Ly6c1	5.49	4.74E-09	1.50E-06
17410905	AI747448	5.41	2.22E-10	1.76E-07
17253707	Nos2	4.96	4.46E-08	8.38E-06
17399374	Muc1	4.79	1.27E-09	5.52E-07
17254166	Slfn2	4.62	5.41E-08	9.64E-06
17261608	Gabrp	4.57	2.41E-10	1.77E-07
17429632	Mfsd2a	4.43	1.13E-11	2.16E-08
17390738	Duox2	4.37	9.35E-10	4.16E-07
17395777	Ptk6	4.29	6.25E-08	1.03E-05
17408885	Chi3l7	4.24	8.20E-07	7.47E-05
17378827	Lbp	4.23	2.17E-09	8.33E-07
17395079	Zbp1	4.14	2.10E-10	1.76E-07
17455954	Tac1	4.12	1.49E-08	3.59E-06
17353663	Tmem173	3.98	9.16E-11	1.04E-07
17548166	LOC100862491	3.94	1.28E-05	6.42E-04
17344765	Trim15	3.77	6.31E-11	8.20E-08
17393789	Tgm2	3.69	1.93E-10	1.72E-07
17438975	Cxcl3	3.49	2.43E-05	1.05E-03
17504074	Cpne2	3.39	1.15E-11	2.16E-08
17399883	Sprr2h	3.34	1.56E-06	1.23E-04
17272619	Socs3	3.33	4.92E-09	1.50E-06
17308099	Adam28	3.28	7.85E-07	7.33E-05
17512868	Chst4	3.25	8.77E-10	4.01E-07
17452054	Oas2	3.06	1.22E-07	1.62E-05
17438963	Ppbp	3.05	1.19E-05	6.09E-04
17545764	Ppef1	2.95	3.63E-06	2.37E-04
17254295	Expi	2.86	7.35E-08	1.14E-05
17353650	Ecscr	2.85	3.37E-10	2.28E-07
17482943	Il4ra	2.84	1.14E-08	2.93E-06

17254176	Slfn4	2.81	1.22E-06	9.79E-05
17401261	Bcl2l15	2.77	3.45E-06	2.31E-04
17269333	Krt36	2.76	9.23E-07	8.09E-05
17344774	Trim40	2.75	9.87E-08	1.42E-05
17437213	Cd38	2.73	4.43E-10	2.78E-07
17514592	Mmp7	2.67	8.64E-07	7.70E-05
17357971	Gna14	2.66	1.13E-07	1.54E-05
17286190	Prl2a1	2.61	5.71E-05	1.95E-03
17394153	Slpi	2.61	5.69E-10	3.11E-07
17497525	Adam8	2.58	9.29E-08	1.38E-05
17526836	Tex12	2.58	6.61E-05	2.19E-03
17499962	Plat	2.56	1.61E-08	3.77E-06
17334192	Prss27	2.54	3.46E-07	3.81E-05
17420539	Pla2g2a	2.51	4.96E-06	3.03E-04
17329195	Thpo	2.50	2.37E-07	2.85E-05
17363470	Gda	2.49	4.65E-10	2.78E-07
17318070	2010109I03Rik	2.48	2.29E-10	1.76E-07
17213666	Klf7	2.47	2.15E-05	9.60E-04
17431820	Pla2g5	2.46	3.54E-07	3.86E-05
17390810	Gatm	2.46	1.21E-05	6.15E-04
17286707	Gcnt2	2.45	6.22E-08	1.03E-05
17319364	D730005E14Rik	2.43	5.84E-08	9.98E-06
17509565	Anxa10	2.42	1.09E-04	3.14E-03
17384457	Stom	2.40	6.48E-10	3.23E-07
17341671	Prss22	2.40	1.59E-08	3.77E-06
17516412	2610203C20Rik	2.39	1.16E-05	6.02E-04
17347163	Xdh	2.38	4.53E-08	8.38E-06
17520624	Rbp1	2.37	5.48E-06	3.31E-04
17428253	Slc5a9	2.35	2.19E-07	2.68E-05
17531701	Gm20317	2.32	3.84E-05	1.48E-03
17491941	Aldh1a3	2.29	1.75E-09	7.21E-07
17242785	Sbno2	2.29	1.17E-08	2.96E-06
17430576	Tinagl1	2.27	1.21E-08	3.02E-06
17463443	Clec2h	2.24	6.02E-10	3.18E-07
17257165	Arf2	2.23	3.04E-08	5.94E-06
17410332	Ccdc109b	2.21	1.20E-06	9.72E-05
17545785	Cdkl5	2.21	4.49E-07	4.54E-05
17533713	Timp1	2.20	6.89E-05	2.25E-03
17438987	Cxcl1	2.19	5.29E-07	5.23E-05
17238172	Rdh16	2.19	4.11E-05	1.54E-03
17269404	Krt16	2.18	1.43E-04	3.83E-03
17267418	A130040M12Rik	2.17	4.73E-04	9.45E-03
17352862	Aqp4	2.17	2.16E-07	2.66E-05
17402519	Tifa	2.16	1.66E-04	4.32E-03
17284839	Itgb8	2.14	3.01E-06	2.08E-04

17410880	Clca4	2.14	3.41E-09	1.20E-06
17543870	Zdhhc15	2.11	2.92E-05	1.20E-03
17377778	Id1	2.11	4.56E-09	1.48E-06
17391554	Il1a	2.09	2.24E-04	5.38E-03
17276934	Ttc9	2.09	2.49E-05	1.07E-03
17318036	Gml	2.08	1.11E-05	5.80E-04
17470060	Rassf4	2.07	6.80E-05	2.23E-03
17469541	Il5ra	2.06	1.06E-07	1.48E-05
17318020	Ly6d	2.03	1.11E-07	1.52E-05
17320583	Cpne8	2.02	1.94E-06	1.45E-04
17468018	Hk2	2.01	2.30E-08	4.87E-06
17264835	Cd68	2.00	4.40E-07	4.48E-05
17226447	Nckap5	2.00	1.15E-04	3.26E-03
17282069	Gpx2	2.00	5.02E-04	9.89E-03
17258887	Pgs1	1.99	8.30E-07	7.51E-05
17515074	Icam1	1.99	9.81E-07	8.46E-05
17401345	Ppm1j	1.99	1.70E-07	2.16E-05
17307425	LOC100862065	1.98	8.89E-05	2.69E-03
17381630	Pfkfb3	1.98	4.52E-07	4.55E-05
17445325	Fzd1	1.97	2.24E-08	4.86E-06
17330080	Dirc2	1.97	5.74E-08	9.98E-06
17313050	Apobec3	1.96	3.22E-07	3.65E-05
17330967	Nfkbiz	1.95	1.09E-06	9.08E-05
17484601	1190003J15Rik	1.94	8.20E-07	7.47E-05
17261107	Rel	1.93	1.77E-07	2.20E-05
17260236	Myl7	1.93	6.65E-05	2.20E-03
17404464	Tmem212	1.92	2.20E-04	5.31E-03
17254041	Ccl2	1.92	3.28E-04	7.15E-03
17344132	Hspa1a	1.90	2.12E-06	1.55E-04
17491163	Tph1	1.90	4.27E-05	1.58E-03
17270583	Nsf	1.89	7.20E-09	2.06E-06
17418271	Fhl3	1.89	6.76E-08	1.09E-05
17356447	Efemp2	1.89	1.61E-06	1.25E-04
17371427	Dhrs9	1.89	6.42E-08	1.04E-05
17489399	Fxyd5	1.88	2.16E-04	5.24E-03
17297125	Ptprg	1.87	4.34E-06	2.74E-04
17439830	Spp1	1.86	4.12E-05	1.54E-03
17282664	Pgf	1.85	6.81E-05	2.23E-03
17230102	Ifi202b	1.85	2.58E-04	5.91E-03
17410204	Alpk1	1.85	6.56E-06	3.80E-04
17410845	Clca1	1.84	1.85E-04	4.70E-03
17388999	Prrg4	1.84	3.79E-07	4.03E-05
17306906	Ripk3	1.83	5.08E-08	9.15E-06
17212947	Spats2l	1.83	6.71E-07	6.45E-05
17238162	Rdh9	1.82	1.03E-05	5.45E-04

17379169	Sgk2	1.81	9.94E-07	8.53E-05
17438113	Cwh43	1.81	8.08E-08	1.24E-05
17327514	B3galt5	1.80	1.56E-05	7.52E-04
17421312	Dhrs3	1.80	1.15E-07	1.55E-05
17239142	Samd5	1.79	7.67E-06	4.28E-04
17353747	Cd14	1.79	2.70E-06	1.91E-04
17246810	Lif	1.79	3.68E-05	1.44E-03
17487457	Bcl3	1.79	6.05E-08	1.01E-05
17230111	Ifi205	1.78	7.04E-06	4.01E-04
17419553	Map3k6	1.77	8.30E-06	4.59E-04
17330870	Gm4827	1.77	2.27E-06	1.65E-04
17348105	Bambi	1.77	4.12E-05	1.54E-03
17513076	Mlkl	1.76	7.08E-07	6.69E-05
17350267	Myot	1.76	1.24E-04	3.42E-03
17521194	Dusp7	1.76	3.49E-06	2.32E-04
17400622	Lix1l	1.76	1.21E-04	3.37E-03
17359113	O3far1	1.75	2.81E-06	1.97E-04
17338959	Tnfsf9	1.73	2.20E-04	5.31E-03
17261575	Ranbp17	1.72	3.29E-04	7.18E-03
17370807	Fmnl2	1.72	6.28E-05	2.11E-03
17383892	Lcn2	1.72	9.28E-05	2.78E-03
17545936	Car5b	1.71	2.34E-05	1.02E-03
17306098	Rnase1	1.71	6.79E-05	2.23E-03
17230195	Cep170	1.71	1.91E-04	4.79E-03
17228624	Rasal2	1.71	2.12E-05	9.53E-04
17283445	Lgmn	1.70	5.16E-05	1.82E-03
17534051	Il13ra1	1.69	5.73E-07	5.63E-05
17419020	Marcksl1	1.69	1.03E-04	3.01E-03
17355366	Smad4	1.69	4.21E-07	4.39E-05
17429689	Macf1	1.69	1.09E-04	3.15E-03
17364126	Gm14446	1.68	2.03E-04	4.98E-03
17313811	Fbln1	1.68	1.23E-05	6.24E-04
17351465	Tubb6	1.68	2.67E-04	6.10E-03
17344126	Hspa1b	1.68	1.59E-04	4.19E-03
17286283	Dusp22	1.68	2.02E-05	9.18E-04
17340584	Tmem181c-ps	1.68	1.21E-04	3.37E-03
17268066	Gm11545	1.68	7.23E-06	4.09E-04
17460831	Aldh1l1	1.68	2.62E-06	1.86E-04
17344309	Tnf	1.68	3.46E-06	2.31E-04
17458682	Creb5	1.67	4.66E-05	1.67E-03
17244913	Zdhhc17	1.67	1.35E-05	6.78E-04
17259026	Card14	1.67	8.38E-06	4.62E-04
17321318	Fkbp11	1.66	1.24E-04	3.42E-03
17480924	Gm19765	1.66	8.06E-05	2.51E-03
17482133	Tmc5	1.66	1.54E-05	7.47E-04

17330099	Parp14	1.66	4.17E-07	4.38E-05
17235077	Cnn2	1.65	5.66E-05	1.95E-03
17506230	6430548M08Rik	1.65	1.06E-05	5.59E-04
17267420	Ypel2	1.65	3.86E-04	8.04E-03
17390778	Duoxa1	1.65	1.14E-04	3.24E-03
17426944	Fam154a	1.64	5.61E-05	1.94E-03
17223985	Ikzf2	1.64	1.11E-05	5.80E-04
17438995	Cxcl2	1.64	2.31E-05	1.02E-03
17332554	Fam3b	1.64	9.45E-07	8.20E-05
17404432	Fndc3b	1.64	2.75E-06	1.94E-04
17337228	Ier3	1.63	6.95E-07	6.61E-05
17292891	Pdlim7	1.63	2.37E-06	1.70E-04
17276328	Hif1a	1.63	3.44E-07	3.81E-05
17437598	0610040J01Rik	1.62	3.08E-05	1.26E-03
17462202	Rasgef1a	1.62	1.35E-05	6.78E-04
17478277	Zdhhc13	1.61	3.10E-07	3.54E-05
17325274	Parp9	1.61	4.66E-06	2.90E-04
17373550	Trp53i11	1.61	2.61E-07	3.06E-05
17285559	Elmo1	1.61	1.04E-04	3.02E-03
17500327	Eif4ebp1	1.61	8.21E-07	7.47E-05
17445089	Fry	1.60	4.05E-04	8.35E-03
17522555	Ltf	1.60	1.55E-04	4.11E-03
17320322	Mapk11	1.60	6.41E-05	2.14E-03
17350281	Dcp2	1.59	3.86E-05	1.48E-03
17362869	AW112010	1.59	4.37E-07	4.48E-05
17270508	Plekhm1	1.59	5.50E-06	3.31E-04
17223903	Acadl	1.59	2.02E-06	1.50E-04
17248270	Hbq1b	1.58	1.77E-04	4.54E-03
17212138	Map4k4	1.58	7.02E-06	4.01E-04
17447803	Fbxl5	1.58	3.08E-05	1.26E-03
17424077	B4galt1	1.58	1.61E-06	1.25E-04
17482095	Nucb2	1.58	4.36E-07	4.48E-05
17402361	Sec24d	1.58	6.38E-05	2.14E-03
17244341	Elk3	1.57	1.01E-05	5.42E-04
17301147	Fam124a	1.57	7.34E-05	2.39E-03
17343278	Hsf2bp	1.57	2.45E-04	5.70E-03
17317015	Ext1	1.57	5.93E-07	5.76E-05
17459196	Tnip3	1.57	2.01E-06	1.50E-04
17211795	Arid5a	1.56	4.97E-05	1.77E-03
17492239	AU020206	1.56	1.55E-05	7.48E-04
17304246	Appl1	1.56	1.53E-06	1.21E-04
17404947	Ankrd50	1.56	4.25E-05	1.57E-03
17215565	Sh3bp4	1.56	2.71E-05	1.14E-03
17349582	1700066B19Rik	1.55	2.95E-04	6.59E-03
17407850	Ecm1	1.55	1.93E-04	4.81E-03

17420016	Id3	1.55	1.17E-04	3.29E-03
17313998	Creld2	1.55	4.27E-06	2.71E-04
17324883	Muc4	1.54	8.75E-05	2.66E-03
17478301	Nav2	1.54	1.75E-04	4.51E-03
17226708	Il24	1.54	1.11E-06	9.11E-05
17468926	LOC100861972	1.53	1.61E-04	4.23E-03
17215629	Mlph	1.53	6.05E-05	2.04E-03
17443166	Stx1a	1.53	1.75E-04	4.51E-03
17248288	Sh3pxd2b	1.53	5.57E-06	3.33E-04
17316690	Klf10	1.53	1.42E-04	3.81E-03
17521621	Amigo3	1.52	1.46E-04	3.91E-03
17346155	Sema6b	1.52	4.21E-05	1.56E-03
17474974	Plaur	1.52	1.10E-06	9.08E-05
17390066	Ehd4	1.52	3.22E-05	1.30E-03
17397426	Ccrn4l	1.52	1.34E-04	3.64E-03
17536364	Msn	1.52	2.01E-05	9.13E-04
17391332	Mal	1.52	1.45E-05	7.15E-04
17538194	Vsig1	1.51	1.57E-04	4.15E-03
17355298	2310002L13Rik	1.51	1.52E-05	7.38E-04
17532472	Cdcp1	1.51	1.74E-05	8.11E-04
17253431	Flot2	1.51	3.63E-05	1.44E-03
17361850	Cdc42ep2	1.51	9.88E-05	2.91E-03
17243617	Ckap4	1.50	7.67E-06	4.28E-04
17336446	Psmb8	1.50	2.36E-04	5.55E-03
17364948	Slc25a28	1.50	1.73E-05	8.07E-04
17439164	Sep-11	1.50	7.63E-05	2.43E-03
17548059	4930578L24Rik	1.50	3.57E-04	7.61E-03
17328124	Litaf	1.50	1.03E-05	5.45E-04
17326166	Cd47	1.49	2.08E-06	1.53E-04
17516194	Vwa5a	1.49	3.09E-04	6.81E-03
17436607	Sh3bp2	1.49	3.56E-05	1.42E-03
17227266	Elf3	1.49	1.01E-05	5.42E-04
17274558	Mboat2	1.49	1.17E-04	3.29E-03
17519967	Tpbg	1.49	4.51E-05	1.64E-03
17291803	1300014I06Rik	1.49	2.70E-05	1.13E-03
17399672	Slc39a1	1.49	6.04E-05	2.04E-03
17326510	Pros1	1.48	1.52E-05	7.38E-04
17283930	Wars	1.48	3.27E-06	2.22E-04
17338258	AI661453	1.48	5.80E-05	1.97E-03
17244140	Apaf1	1.48	1.52E-04	4.05E-03
17361285	Syt12	1.48	2.42E-05	1.05E-03
17370977	Pkp4	1.48	2.38E-05	1.04E-03
17441037	Oasl2	1.48	1.80E-04	4.60E-03
17433251	H6pd	1.47	2.94E-04	6.58E-03
17516564	Hyou1	1.47	4.28E-05	1.58E-03

17439021	Ereg	1.47	2.55E-04	5.85E-03
17406552	Pear1	1.47	9.76E-05	2.89E-03
17501976	Isyna1	1.47	5.35E-05	1.87E-03
17246967	Xbp1	1.47	6.48E-06	3.76E-04
17370103	Grp78 (Hspa5)	1.46	6.98E-06	4.00E-04
17514435	Casp4	1.46	3.19E-06	2.18E-04
17230595	Degs1	1.46	1.18E-05	6.05E-04
17314011	Pim3	1.46	1.33E-04	3.63E-03
17217610	Csrp1	1.46	7.38E-05	2.39E-03
17530457	Atp2c1	1.46	1.40E-04	3.77E-03
17512554	Psmb10	1.45	5.89E-06	3.48E-04
17432674	Tnfrsf1b	1.45	4.38E-04	8.89E-03
17450121	Plac8	1.45	2.24E-05	9.90E-04
17319380	Pdgfb	1.45	3.64E-04	7.72E-03
17528111	Smad3	1.45	9.59E-05	2.84E-03
17496049	Nsmce1	1.45	2.82E-05	1.17E-03
17518142	Tle3	1.44	4.30E-04	8.78E-03
17249593	Irf1	1.44	5.38E-05	1.87E-03
17329433	Bcl6	1.44	7.60E-05	2.43E-03
17269717	Stat3	1.44	4.58E-06	2.86E-04
17453160	Gusb	1.44	1.88E-04	4.75E-03
17228136	Lamc2	1.43	1.27E-04	3.49E-03
17316175	Basp1	1.43	7.99E-05	2.49E-03
17543336	Las1l	1.43	3.99E-05	1.51E-03
17348648	Taf4b	1.43	3.38E-04	7.34E-03
17341681	Kctd5	1.43	5.19E-05	1.83E-03
17509682	BC030870	1.43	4.60E-04	9.25E-03
17343143	Glo1	1.43	1.63E-05	7.77E-04
17315438	Prr13	1.43	4.75E-05	1.70E-03
17282226	Actn1	1.43	6.98E-05	2.27E-03
17358598	Il33	1.43	7.41E-05	2.40E-03
17289889	Il6st	1.43	3.00E-04	6.66E-03
17464718	Asns	1.43	1.71E-05	8.03E-04
17218060	Ptgs2	1.42	8.43E-05	2.58E-03
17244070	Nr1h4	1.42	1.19E-04	3.32E-03
17418916	Rnf19b	1.42	2.68E-04	6.11E-03
17266520	Lgals9	1.42	1.73E-04	4.47E-03
17354595	2010002N04Rik	1.42	4.21E-04	8.64E-03
17456554	Hilpda	1.42	2.07E-04	5.06E-03
17214665	Sgpp2	1.42	2.92E-05	1.20E-03
17427860	Acot11	1.42	1.12E-05	5.80E-04
17516546	Dpagt1	1.42	1.85E-05	8.54E-04
17468298	Exoc6b	1.41	2.30E-05	1.01E-03
17487489	Pvr	1.41	1.05E-04	3.04E-03
17356490	Rela	1.40	1.73E-05	8.07E-04

17281148	Baz1a	1.40	1.19E-04	3.32E-03
17377092	Sec23b	1.40	9.41E-05	2.80E-03
17439622	Agpat9	1.40	5.10E-05	1.81E-03
17495549	Itpripl2	1.40	3.21E-04	7.04E-03
17452552	Rhof	1.40	4.09E-04	8.41E-03
17418540	Stk40	1.40	2.32E-04	5.50E-03
17353699	Hbegf	1.40	2.91E-05	1.20E-03
17356662	Batf2	1.39	4.58E-04	9.22E-03
17367192	Arl5b	1.39	5.70E-05	1.95E-03
17282868	Sptlc2	1.39	3.31E-05	1.32E-03
17548321	LOC100862248	1.39	1.14E-04	3.24E-03
17279919	Rhob	1.39	7.81E-05	2.47E-03
17466060	Parp12	1.39	9.20E-05	2.76E-03
17480018	Ctsc	1.39	2.64E-05	1.12E-03
17525240	St14	1.39	8.83E-06	4.80E-04
17466783	Skap2	1.39	3.75E-04	7.90E-03
17424656	Tpm2	1.38	4.31E-04	8.79E-03
17511453	N4bp1	1.38	1.89E-04	4.76E-03
17321761	Smagp	1.38	1.84E-05	8.54E-04
17539210	Sh3kbp1	1.38	1.60E-04	4.22E-03
17317046	Tnfrsf11b	1.38	3.90E-05	1.49E-03
17414836	Tlr4	1.38	1.97E-04	4.88E-03
17442177	Orai1	1.38	2.28E-04	5.45E-03
17392333	Rrbp1	1.38	4.04E-05	1.53E-03
17377753	H13	1.38	2.67E-05	1.12E-03
17295136	Iqgap2	1.37	8.32E-05	2.56E-03
17255232	Lrrc59	1.37	1.39E-05	6.87E-04
17410410	Sgms2	1.37	2.56E-05	1.10E-03
17274751	Sh3yl1	1.37	3.84E-04	8.02E-03
17325093	Muc13	1.36	1.63E-05	7.77E-04
17314460	Irak4	1.36	3.70E-04	7.82E-03
17346840	Rab12	1.36	2.01E-04	4.96E-03
17385684	Rbms1	1.36	2.60E-05	1.11E-03
17529322	Ibtk	1.36	7.47E-05	2.41E-03
17471062	Tapbpl	1.36	1.92E-04	4.80E-03
17508691	Rbpms	1.36	3.26E-04	7.12E-03
17502378	Tpm4	1.36	3.86E-05	1.48E-03
17314061	Trabd	1.36	8.27E-05	2.55E-03
17338946	Crb3	1.36	4.90E-04	9.74E-03
17248561	Atp10b	1.36	7.79E-05	2.47E-03
17385654	Itgb6	1.36	1.87E-04	4.73E-03
17245302	Rap1b	1.35	3.14E-05	1.27E-03
17513713	Fam38a	1.35	1.15E-04	3.25E-03
17239090	Tab2	1.35	2.51E-04	5.78E-03
17327524	Igsf5	1.35	1.33E-04	3.63E-03

17231859	Ifngr1	1.34	3.79E-05	1.47E-03
17432341	Efhd2	1.34	5.50E-05	1.91E-03
17428142	Osbpl9	1.34	8.33E-05	2.56E-03
17222825	Obfc2a	1.34	7.61E-05	2.43E-03
17224661	Epha4	1.34	1.65E-04	4.31E-03
17271118	Pitpnc1	1.34	2.50E-04	5.77E-03
17318013	Lynx1	1.33	4.43E-05	1.61E-03
17453496	Cldn4	1.33	3.33E-05	1.33E-03
17299288	Samd4	1.33	1.81E-04	4.61E-03
17513641	Slc7a5	1.33	4.35E-04	8.86E-03
17342101	Hn1l	1.33	1.60E-04	4.21E-03
17292722	Cltb	1.33	7.83E-05	2.48E-03
17439029	Areg	1.33	4.01E-05	1.52E-03
17492598	Furin	1.33	4.29E-04	8.76E-03
17377597	Fkbp1a	1.33	3.70E-04	7.82E-03
17445786	Ptpn12	1.32	1.46E-04	3.91E-03
17351414	Pmaip1	1.32	4.74E-04	9.45E-03
17227278	Rnpep	1.32	5.59E-05	1.93E-03
17385967	Galnt3	1.32	6.56E-05	2.19E-03
17483925	Dmbt1	1.32	4.67E-04	9.35E-03
17495262	Rras2	1.32	7.72E-05	2.46E-03
17517105	Il18	1.32	1.55E-04	4.10E-03
17285000	Klf6	1.32	3.02E-04	6.69E-03
17456176	Met	1.32	2.87E-04	6.50E-03
17297305	Ngly1	1.31	1.14E-04	3.24E-03
17361741	Ehbp1l1	1.31	3.59E-04	7.64E-03
17398903	Nes	1.31	6.79E-05	2.23E-03
17335297	Ppard	1.31	2.43E-04	5.67E-03
17358119	Zfand5	1.31	9.33E-05	2.79E-03
17298775	Anxa8	1.31	2.33E-04	5.52E-03
17274249	Pdia6	1.31	4.93E-05	1.76E-03
17530000	Pik3cb	1.31	3.46E-04	7.48E-03
17295125	F2rl1	1.31	8.88E-05	2.69E-03
17286231	Sox4	1.30	2.24E-04	5.38E-03
17496345	Nupr1	1.30	2.05E-04	5.02E-03
17268137	Itga3	1.30	2.06E-04	5.04E-03
17312280	Gsdmd	1.30	1.05E-04	3.04E-03
17526206	C2cd2l	1.30	2.43E-04	5.67E-03
17325619	Tmem39a	1.30	2.91E-04	6.57E-03
17464128	Golt1b	1.30	1.80E-04	4.60E-03
17405737	Lxn	1.30	2.21E-04	5.32E-03
17475597	Sertad1	1.30	3.91E-04	8.10E-03
17363374	Ostf1	1.30	3.62E-04	7.70E-03
17438062	Nipal1	1.30	1.66E-04	4.32E-03
17337467	Trim31	1.30	3.50E-04	7.51E-03

17327995	Carhsp1	1.29	4.52E-04	9.14E-03
17381043	Tpd52l2	1.29	2.88E-04	6.50E-03
17469887	2510049J12Rik	1.29	1.24E-04	3.42E-03
17274415	Asap2	1.29	4.93E-04	9.78E-03
17341589	Paqr4	1.28	1.19E-04	3.32E-03
17530322	Uba5	1.28	3.90E-04	8.10E-03
17418934	Tmem54	1.28	1.81E-04	4.61E-03
17508300	Adam9	1.28	1.23E-04	3.42E-03
17528202	Rab11a	1.28	3.47E-04	7.48E-03
17363407	Anxa1	1.28	1.14E-04	3.24E-03
17290242	Net1	1.28	1.52E-04	4.04E-03
17510422	B3gnt3	1.28	2.41E-04	5.65E-03
17410376	Ostc	1.28	2.02E-04	4.97E-03
17442096	Arpc3	1.28	2.13E-04	5.18E-03
17268493	Npepps	1.27	2.13E-04	5.18E-03
17488458	Zfp36	1.26	4.25E-04	8.71E-03
17531987	Tgfbr2	1.26	3.94E-04	8.15E-03
17336896	Clic1	1.26	3.87E-04	8.05E-03
17350356	Eif1a	1.26	2.32E-04	5.50E-03
17233736	Sar1a	1.26	4.88E-04	9.70E-03
17335842	Pde9a	1.25	3.76E-04	7.91E-03
17225760	Stk25	1.25	3.65E-04	7.72E-03
17336788	Slc44a4	1.25	1.99E-04	4.92E-03
17429613	Cap1	1.24	2.57E-04	5.89E-03
17406760	Sema4a	1.24	3.62E-04	7.70E-03
17384632	Strbp	0.82	4.56E-04	9.20E-03
17282563	Npc2	0.80	4.95E-04	9.80E-03
17273292	Dcxr	0.79	2.31E-04	5.50E-03
17404180	Car1	0.79	2.48E-04	5.74E-03
17506938	Irf2bp2	0.78	2.95E-04	6.59E-03
17311315	Ttc35	0.78	4.99E-04	9.85E-03
17361073	Gstp1	0.78	1.87E-04	4.74E-03
17223138	Pgap1	0.78	3.34E-04	7.26E-03
17235268	Ndufs7	0.78	5.03E-04	9.90E-03
17250734	2410006H16Rik	0.78	3.05E-04	6.74E-03
17469259	Suclg2	0.78	2.34E-04	5.54E-03
17501050	Irf2	0.78	3.48E-04	7.49E-03
17429057	Ptprf	0.77	3.64E-04	7.72E-03
17329723	Acap2	0.77	1.72E-04	4.46E-03
17261865	Ccng1	0.77	1.02E-04	2.98E-03
17475975	Ech1	0.77	2.29E-04	5.46E-03
17406091	Etfdh	0.77	3.35E-04	7.26E-03
17233799	Slc25a16	0.77	3.78E-04	7.93E-03
17300429	Dhrs4	0.77	2.72E-04	6.19E-03
17335493	BC004004	0.77	2.14E-04	5.19E-03

17391608	Idh3b	0.77	2.93E-04	6.57E-03
17415863	Cachd1	0.77	3.50E-04	7.50E-03
17265129	Acadvl	0.77	1.80E-04	4.60E-03
17364932	Got1	0.76	2.40E-04	5.62E-03
17225775	Thap4	0.76	1.02E-04	2.99E-03
17339991	Eml4	0.76	8.96E-05	2.70E-03
17315187	9430023L20Rik	0.76	5.19E-05	1.83E-03
17390823	Slc30a4	0.76	1.97E-04	4.88E-03
17463169	Scnn1a	0.76	8.25E-05	2.55E-03
17295308	Rgnef	0.76	3.02E-04	6.69E-03
17360331	Mxi1	0.76	2.33E-04	5.52E-03
17421828	Clstn1	0.75	7.96E-05	2.49E-03
17526813	Pts	0.75	2.16E-04	5.24E-03
17360084	As3mt	0.75	1.74E-04	4.50E-03
17548296	LOC100503320	0.75	2.48E-04	5.74E-03
17281908	Tmem30b	0.75	1.19E-04	3.32E-03
17255691	Prr15l	0.75	4.66E-05	1.67E-03
17423544	Wwp1	0.75	4.37E-04	8.88E-03
17514424	Casp1	0.75	1.91E-04	4.79E-03
17441949	Aldh2	0.75	8.38E-05	2.57E-03
17504293	Mmp15	0.75	8.64E-05	2.63E-03
17481960	Arntl	0.75	1.17E-04	3.29E-03
17210984	Pcmtd1	0.75	1.40E-04	3.77E-03
17347751	Lrpprc	0.75	1.76E-04	4.52E-03
17393684	Rbl1	0.75	4.62E-04	9.27E-03
17252497	Atp2a3	0.74	4.88E-04	9.70E-03
17343164	Tff3	0.74	7.61E-05	2.43E-03
17336077	Zfp955b	0.74	4.74E-04	9.45E-03
17370924	Galnt5	0.74	1.93E-04	4.82E-03
17487001	Hif3a	0.74	4.83E-04	9.61E-03
17302766	Rap2a	0.74	3.54E-04	7.56E-03
17409736	Slc35a3	0.74	3.86E-04	8.04E-03
17469656	Srgap3	0.74	2.41E-04	5.65E-03
17393592	Ndrg3	0.74	9.87E-05	2.91E-03
17397475	Mgst2	0.74	7.91E-05	2.49E-03
17443286	Por	0.74	7.58E-05	2.43E-03
17470301	Adipor2	0.74	1.19E-04	3.32E-03
17276743	Plekhh1	0.74	1.81E-04	4.61E-03
17328277	Pla2g10	0.74	4.37E-05	1.60E-03
17509976	Slc25a42	0.74	3.14E-04	6.91E-03
17509462	Galnt7	0.74	9.33E-05	2.79E-03
17302305	Tdrd3	0.74	4.09E-05	1.54E-03
17242046	Dip2a	0.73	5.80E-05	1.97E-03
17405142	Setd7	0.73	2.93E-04	6.57E-03
17218149	Ivns1abp	0.73	9.61E-05	2.85E-03

17498786	Lrrc8e	0.73	3.96E-05	1.51E-03
17525263	Aplp2	0.73	8.40E-05	2.57E-03
17426497	Megf9	0.73	3.92E-05	1.50E-03
17544424	Nox1	0.73	1.46E-04	3.91E-03
17213548	Nrp2	0.73	1.15E-04	3.26E-03
17215576	Agap1	0.73	2.86E-04	6.48E-03
17384021	Stxbp1	0.73	2.50E-04	5.77E-03
17411323	Acadm	0.73	4.35E-05	1.60E-03
17499396	Fbxo25	0.73	4.94E-04	9.79E-03
17313087	Mgat3	0.72	2.68E-05	1.13E-03
17356041	Acy3	0.72	1.62E-04	4.24E-03
17320666	Slc2a13	0.72	3.18E-04	7.01E-03
17378359	Acss2	0.72	2.36E-04	5.55E-03
17514732	Sesn3	0.72	4.42E-04	8.97E-03
17505718	Pdpr	0.72	4.66E-04	9.34E-03
17484014	Acadsb	0.72	6.05E-05	2.04E-03
17542149	Ids	0.72	2.36E-04	5.55E-03
17527735	Neo1	0.72	2.01E-04	4.96E-03
17331669	App	0.72	1.27E-05	6.42E-04
17411547	Chchd7	0.72	3.81E-04	7.96E-03
17479875	Stard5	0.72	1.97E-04	4.88E-03
17355790	Tshz1	0.72	4.25E-04	8.71E-03
17532418	Ano10	0.72	2.49E-04	5.76E-03
17517448	Agphd1	0.72	1.36E-04	3.67E-03
17233039	Rtn4ip1	0.71	3.81E-04	7.96E-03
17527123	Acat1	0.71	6.88E-05	2.25E-03
17426981	Plin2	0.71	2.75E-04	6.25E-03
17274813	Slc26a3	0.71	3.45E-04	7.46E-03
17266107	Abr	0.71	3.33E-04	7.26E-03
17319150	Baiap2l2	0.71	3.47E-04	7.48E-03
17233394	Smpdl3a	0.71	5.31E-05	1.86E-03
17345004	LOC100862363	0.71	1.63E-04	4.26E-03
17234066	Ank3	0.71	9.36E-05	2.79E-03
17524156	4931406C07Rik	0.71	1.68E-05	7.95E-04
17400403	Golph3l	0.71	3.77E-05	1.47E-03
17310912	Laptm4b	0.71	2.13E-05	9.57E-04
17231717	Fuca2	0.71	2.49E-05	1.07E-03
17396162	Car2	0.71	1.99E-05	9.11E-04
17400759	Acp6	0.71	2.39E-05	1.04E-03
17343735	H2-Ke6	0.71	2.37E-04	5.56E-03
17530159	Ppp2r3a	0.71	4.03E-04	8.31E-03
17442149	P2rx4	0.71	8.78E-05	2.66E-03
17284222	Ahnak2	0.70	3.06E-04	6.77E-03
17434614	4930420K17Rik	0.70	4.48E-04	9.07E-03
17321790	Galnt6	0.70	2.26E-04	5.41E-03

17467154	Ppm1k	0.70	1.48E-04	3.94E-03
17397957	P2ry1	0.70	2.05E-05	9.27E-04
17435584	Insig1	0.70	4.35E-05	1.60E-03
17331059	Gpr128	0.70	8.74E-06	4.77E-04
17475026	Ethe1	0.70	1.50E-05	7.36E-04
17212286	Slc9a2	0.70	5.06E-05	1.79E-03
17243576	Appl2	0.70	2.65E-05	1.12E-03
17443401	Rasa4	0.70	9.56E-05	2.84E-03
17514799	2200002K05Rik	0.70	7.73E-05	2.46E-03
17532045	Plcd1	0.70	4.26E-04	8.71E-03
17334253	Eci1	0.70	2.90E-05	1.20E-03
17251994	Slc16a11	0.70	8.12E-05	2.53E-03
17279365	Siva1	0.70	7.98E-05	2.49E-03
17480216	Prcp	0.70	3.61E-05	1.43E-03
17430216	Gjb3	0.69	1.27E-04	3.49E-03
17534909	Ddx26b	0.69	4.74E-04	9.45E-03
17475734	Fcgbp	0.69	8.14E-05	2.53E-03
17411751	Trp53inp1	0.69	2.25E-05	9.93E-04
17312032	Ndrg1	0.69	3.58E-05	1.42E-03
17459676	Retsat	0.69	5.31E-05	1.86E-03
17309161	Fbxl3	0.69	2.84E-06	1.98E-04
17477897	Bcat2	0.69	4.42E-06	2.77E-04
17219356	Tstd1	0.69	4.69E-06	2.91E-04
17223490	Tmem237	0.69	3.86E-04	8.04E-03
17228375	Xpr1	0.69	1.51E-05	7.37E-04
17520233	Ctsh	0.69	4.20E-05	1.56E-03
17402283	Gclm	0.69	1.69E-05	7.96E-04
17297227	Slc4a7	0.69	1.04E-04	3.01E-03
17219231	Nr1i3	0.68	1.42E-05	7.01E-04
17268227	Abi3	0.68	3.86E-05	1.48E-03
17339801	Crim1	0.68	3.21E-04	7.04E-03
17232912	Sesn1	0.68	2.50E-04	5.77E-03
17440054	A830010M20Rik	0.68	1.37E-04	3.70E-03
17277195	Ptgr2	0.68	2.32E-04	5.50E-03
17216960	Pigr	0.68	3.27E-05	1.31E-03
17381864	Trdmt1	0.68	3.52E-04	7.52E-03
17445860	Napepld	0.68	1.10E-04	3.15E-03
17336921	Ly6g6c	0.68	2.93E-04	6.57E-03
17477979	Dbp	0.68	3.26E-04	7.12E-03
17297576	Adk	0.68	1.72E-05	8.07E-04
17359769	Sema4g	0.67	1.99E-05	9.11E-04
17309802	Oxct1	0.67	6.18E-06	3.60E-04
17238605	Itga7	0.67	3.79E-05	1.47E-03
17338472	Mocs1	0.67	2.47E-04	5.74E-03
17339313	Epb4.1l3	0.67	1.11E-04	3.17E-03

17367036	Pter	0.67	3.17E-05	1.28E-03
17420777	Aldh4a1	0.67	9.13E-05	2.75E-03
17505455	Phlpp2	0.67	6.58E-05	2.19E-03
17501787	Tm6sf2	0.67	4.00E-04	8.26E-03
17498847	Lass4	0.67	4.63E-05	1.67E-03
17252058	Tm4sf5	0.66	7.90E-05	2.49E-03
17498467	Ano1	0.66	3.84E-05	1.48E-03
17490889	0610005C13Rik	0.66	3.52E-04	7.52E-03
17291355	Acot13	0.66	5.91E-05	2.01E-03
17480788	Fchsd2	0.66	1.30E-04	3.57E-03
17355915	Cpt1a	0.66	1.95E-05	8.98E-04
17495586	Gde1	0.66	4.08E-05	1.54E-03
17455882	Ppp1r9a	0.66	1.47E-04	3.93E-03
17527054	Atm	0.66	2.43E-04	5.67E-03
17469754	Cidec	0.66	9.85E-05	2.91E-03
17229007	Fmo4	0.66	4.09E-04	8.41E-03
17517349	Slc35f2	0.66	4.57E-04	9.21E-03
17438423	Paics	0.66	1.07E-04	3.08E-03
17493980	Lrrc51	0.66	4.53E-05	1.64E-03
17243392	Gna11	0.66	4.87E-06	2.98E-04
17222777	Slc40a1	0.66	6.70E-05	2.22E-03
17363799	5033414D02Rik	0.66	4.58E-05	1.65E-03
17325637	B4galt4	0.66	6.40E-05	2.14E-03
17407764	Prune	0.65	2.53E-05	1.09E-03
17467608	Rmnd5a	0.65	2.00E-05	9.12E-04
17527421	Tspan3	0.65	1.46E-05	7.19E-04
17302889	Pcca	0.65	6.13E-05	2.07E-03
17449033	Ppat	0.65	2.05E-04	5.02E-03
17309688	A2ld1	0.65	5.37E-05	1.87E-03
17388803	Cat	0.65	3.10E-06	2.13E-04
17323838	Klhl24	0.65	7.36E-05	2.39E-03
17493461	Aqp11	0.64	7.75E-06	4.31E-04
17310882	Pgcp	0.64	3.00E-04	6.66E-03
17286487	Bphl	0.64	5.71E-05	1.95E-03
17451171	Hscb	0.64	3.98E-05	1.51E-03
17420064	Tcea3	0.64	4.45E-04	9.00E-03
17284037	Brp44l	0.64	4.99E-04	9.85E-03
17240880	Man1a	0.64	1.04E-05	5.47E-04
17480515	Mtap6	0.64	5.54E-05	1.92E-03
17410390	Hadh	0.64	1.83E-06	1.39E-04
17249702	Fnip1	0.64	2.86E-04	6.48E-03
17265748	Aspa	0.64	1.31E-04	3.58E-03
17363328	Pcsk5	0.63	3.71E-05	1.45E-03
17350090	Sh3rf2	0.63	7.42E-06	4.19E-04
17366511	Phyh	0.63	1.70E-06	1.31E-04

17326964	Hunk	0.63	2.87E-04	6.50E-03
17295503	Mccc2	0.63	3.55E-06	2.34E-04
17218349	Glul	0.63	8.99E-07	7.92E-05
17350364	LOC100861902	0.63	1.56E-05	7.52E-04
17319554	Pmm1	0.63	8.54E-05	2.61E-03
17343505	Cyp4f13	0.63	1.99E-04	4.92E-03
17464400	Rep15	0.63	1.16E-04	3.27E-03
17443486	Cldn15	0.63	8.67E-05	2.64E-03
17489801	Nudt19	0.63	4.22E-05	1.56E-03
17433328	Per3	0.62	1.87E-05	8.62E-04
17397129	Fgf2	0.62	2.95E-06	2.06E-04
17502816	Inpp4b	0.62	6.12E-06	3.59E-04
17533474	Maoa	0.62	2.43E-06	1.74E-04
17291241	Slc17a4	0.62	1.15E-04	3.25E-03
17259810	Inpp5j	0.62	3.71E-06	2.41E-04
17410939	Clca3	0.62	3.57E-06	2.34E-04
17320907	Slc38a1	0.62	1.77E-06	1.35E-04
17388389	Cry2	0.62	2.58E-04	5.91E-03
17423701	Fut9	0.62	1.28E-06	1.02E-04
17288269	Gm10767	0.62	1.89E-04	4.75E-03
17435934	Khk	0.62	2.97E-04	6.62E-03
17235320	Reep6	0.62	3.44E-06	2.31E-04
17259907	Tcn2	0.61	1.12E-06	9.11E-05
17516960	Cadm1	0.61	2.89E-05	1.20E-03
17295588	Naip5	0.61	8.25E-05	2.55E-03
17436407	Slc5a1	0.61	1.10E-04	3.17E-03
17530141	Pccb	0.61	2.23E-05	9.90E-04
17409343	5330417C22Rik	0.61	2.77E-05	1.16E-03
17255466	Gngt2	0.61	2.19E-04	5.30E-03
17303412	Acox2	0.61	5.72E-06	3.39E-04
17359636	Abcc2	0.61	3.77E-05	1.47E-03
17220794	D730003I15Rik	0.61	3.95E-04	8.18E-03
17310837	Sema5a	0.60	4.14E-06	2.64E-04
17311533	Deptor	0.60	3.98E-06	2.55E-04
17427401	Cyp2j9	0.60	3.39E-04	7.35E-03
17214368	Cyp27a1	0.60	4.43E-05	1.61E-03
17307134	Cryl1	0.59	3.89E-06	2.51E-04
17508938	Fgl1	0.59	2.20E-04	5.31E-03
17258220	Slc16a5	0.59	4.91E-05	1.75E-03
17246113	Slc39a5	0.59	3.80E-04	7.96E-03
17339554	Lbh	0.59	1.51E-07	1.98E-05
17351811	Acaa2	0.59	4.27E-07	4.44E-05
17448308	Ugdh	0.59	2.58E-07	3.05E-05
17237208	Csrp2	0.59	1.11E-07	1.52E-05
17243183	Creb3l3	0.59	2.96E-04	6.59E-03

17544691	Bex2	0.59	1.95E-04	4.86E-03
17413674	Aldh1b1	0.59	3.30E-06	2.24E-04
17318983	Tst	0.59	7.91E-07	7.36E-05
17449313	Ugt2b34	0.59	2.90E-07	3.36E-05
17471222	Ccnd2	0.58	3.74E-07	4.00E-05
17240303	G630090E17Rik	0.58	3.21E-04	7.04E-03
17429954	1700029G01Rik	0.58	5.24E-06	3.18E-04
17232135	1110021L09Rik	0.58	1.21E-06	9.72E-05
17339910	Galm	0.58	1.66E-05	7.89E-04
17366670	Itih5	0.58	4.72E-07	4.70E-05
17253648	Slc46a1	0.58	3.49E-06	2.32E-04
17310637	Fam134b	0.58	6.78E-06	3.91E-04
17280310	Id2	0.58	1.02E-06	8.65E-05
17442780	Glt1d1	0.58	7.57E-05	2.43E-03
17306147	Ndrg2	0.58	3.17E-05	1.28E-03
17329298	Etv5	0.58	3.64E-05	1.44E-03
17520905	Slco2a1	0.58	1.02E-05	5.45E-04
17251441	Slc25a35	0.58	5.62E-05	1.94E-03
17250055	1810065E05Rik	0.58	6.15E-06	3.60E-04
17449989	Rasgef1b	0.57	7.63E-06	4.28E-04
17329220	Ehhadh	0.57	6.46E-05	2.16E-03
17277134	Acot2	0.57	7.18E-06	4.07E-04
17310673	Ank	0.57	2.04E-06	1.51E-04
17362717	Dak	0.57	2.08E-05	9.38E-04
17484670	Nlrp6	0.57	4.56E-05	1.65E-03
17310816	Cmbl	0.57	3.78E-06	2.45E-04
17477391	Klk1	0.57	1.03E-06	8.67E-05
17337844	Enpp5	0.56	1.58E-07	2.06E-05
17317284	Anxa13	0.56	8.48E-06	4.64E-04
17459696	Gm15401	0.56	8.47E-06	4.64E-04
17327255	Cbr1	0.56	1.66E-07	2.14E-05
17495958	Ern2	0.56	1.12E-06	9.14E-05
17528934	Tinag	0.56	3.43E-07	3.81E-05
17449725	Cxcl11	0.56	1.37E-05	6.86E-04
17437459	Slc34a2	0.56	7.90E-05	2.49E-03
17302161	1300010F03Rik	0.56	8.21E-07	7.47E-05
17282498	Entpd5	0.56	1.71E-07	2.16E-05
17411420	Cth	0.56	1.67E-05	7.91E-04
17272468	St6galnac2	0.56	4.10E-07	4.33E-05
17466581	Zfp467	0.55	2.92E-04	6.57E-03
17292071	Nedd9	0.55	1.87E-06	1.40E-04
17363559	Fam189a2	0.55	1.72E-04	4.46E-03
17433672	2810405K02Rik	0.55	2.30E-07	2.78E-05
17467209	Fam13a	0.55	1.24E-05	6.30E-04
17489214	Upk1a	0.55	1.71E-04	4.44E-03

17506030	Hsd17b2	0.55	6.02E-08	1.01E-05
17268010	Acsf2	0.55	1.10E-06	9.08E-05
17464210	Lrmp	0.55	1.03E-04	3.00E-03
17284963	Marcksl1-ps4	0.55	1.93E-04	4.82E-03
17404122	Slc10a5	0.54	1.03E-04	3.01E-03
17388332	Creb3l1	0.54	9.91E-08	1.42E-05
17361546	Cst6	0.54	1.17E-05	6.02E-04
17470095	Fxyd4	0.54	3.77E-04	7.92E-03
17443185	Mlxipl	0.54	8.60E-07	7.70E-05
17373283	Lrp4	0.53	4.75E-06	2.93E-04
17220591	5033404E19Rik	0.53	1.64E-04	4.29E-03
17230484	Ephx1	0.53	8.64E-07	7.70E-05
17376124	Slc20a1	0.53	4.95E-08	9.01E-06
17354730	Slc26a2	0.53	3.62E-07	3.93E-05
17260644	Ddc	0.53	1.76E-06	1.35E-04
17517289	Exph5	0.53	3.48E-04	7.49E-03
17449406	Sult1d1	0.53	2.58E-07	3.05E-05
17456736	Ahcyl2	0.53	6.93E-07	6.61E-05
17490589	Fcgrt	0.53	4.39E-06	2.76E-04
17400048	Rorc	0.53	4.23E-06	2.69E-04
17283556	Ifi27l2b	0.53	6.93E-06	3.98E-04
17496354	Sult1a1	0.53	1.92E-04	4.80E-03
17520631	Rbp2	0.52	1.00E-06	8.54E-05
17273864	Apob	0.52	2.87E-05	1.19E-03
17449710	Cxcl9	0.52	3.73E-04	7.86E-03
17534960	Fhl1	0.52	4.73E-07	4.70E-05
17464539	Hepacam2	0.52	2.15E-05	9.60E-04
17408323	Hsd3b2	0.52	7.99E-05	2.49E-03
17319091	Lgals2	0.52	2.91E-04	6.57E-03
17504829	Hsd11b2	0.52	3.93E-06	2.53E-04
17512732	Nqo1	0.52	5.53E-06	3.32E-04
17516098	Siae	0.51	1.68E-05	7.95E-04
17256196	Rapgefl1	0.51	1.03E-05	5.46E-04
17321780	Cela1	0.51	1.24E-06	9.89E-05
17289103	Serinc5	0.51	3.67E-07	3.95E-05
17511693	Ces1d	0.51	4.49E-05	1.63E-03
17226655	C4bp	0.51	2.59E-05	1.11E-03
17321078	Vdr	0.50	9.33E-09	2.54E-06
17284948	Akr1c19	0.50	3.65E-05	1.44E-03
17267066	Dhrs11	0.50	3.51E-06	2.32E-04
17375997	Mertk	0.49	1.25E-04	3.45E-03
17294934	Thbs4	0.49	9.00E-08	1.35E-05
17509031	Gm6329	0.49	2.24E-04	5.38E-03
17257906	Map2k6	0.48	3.23E-05	1.30E-03
17307463	Ebpl	0.48	5.59E-06	3.33E-04

17235730	2210404O07Rik	0.48	3.45E-07	3.81E-05
17488057	Cyp2s1	0.48	3.00E-06	2.08E-04
17482752	2010110P09Rik	0.48	7.35E-08	1.14E-05
17280817	Scin	0.48	7.21E-08	1.14E-05
17268120	Pdk2	0.48	3.66E-09	1.24E-06
17237811	Lrig3	0.48	1.17E-07	1.57E-05
17340135	Slc3a1	0.47	1.04E-07	1.47E-05
17359251	Cyp2c65	0.47	3.11E-06	2.13E-04
17342038	Fahd1	0.47	1.74E-07	2.18E-05
17496584	Zg16	0.47	5.10E-06	3.10E-04
17507605	Gas6	0.46	3.05E-08	5.94E-06
17343488	Cyp4f14	0.46	3.01E-07	3.46E-05
17224015	Abca12	0.46	4.85E-06	2.98E-04
17277267	Aldh6a1	0.45	1.17E-05	6.02E-04
17435643	Rnf32	0.45	1.33E-04	3.62E-03
17247948	Efemp1	0.45	8.87E-06	4.81E-04
17403070	Adh1	0.45	2.88E-08	5.74E-06
17346401	Acer1	0.45	1.57E-06	1.23E-04
17511731	Ces1f	0.44	1.87E-04	4.73E-03
17348860	Mep1b	0.44	4.56E-08	8.38E-06
17539494	Ace2	0.44	1.84E-06	1.39E-04
17363429	Aldh1a7	0.43	1.35E-04	3.67E-03
17409154	Gstm1	0.43	1.01E-04	2.96E-03
17246381	Mettl7b	0.43	2.24E-08	4.86E-06
17476288	Cox7a1	0.43	1.81E-08	4.13E-06
17409142	Gstm2	0.43	1.01E-05	5.42E-04
17327264	Cbr3	0.43	7.99E-09	2.25E-06
17407378	Lor	0.42	5.89E-07	5.76E-05
17434524	Abcb1a	0.42	2.29E-08	4.87E-06
17448001	Ppargc1a	0.42	1.67E-07	2.14E-05
17417946	Guca2a	0.41	2.38E-08	4.90E-06
17253181	Tmigd1	0.41	5.47E-08	9.64E-06
17467269	Hpgds	0.41	2.58E-05	1.10E-03
17321683	Slc11a2	0.41	1.33E-07	1.76E-05
17534528	Xpnpep2	0.41	2.40E-08	4.90E-06
17362861	Ms4a18	0.41	2.93E-05	1.20E-03
17466322	Trpv6	0.41	4.96E-09	1.50E-06
17360312	Add3	0.41	2.44E-08	4.91E-06
17405908	Bche	0.41	7.25E-08	1.14E-05
17456161	Cav1	0.41	1.07E-06	8.93E-05
17295278	Hexb	0.40	3.19E-08	6.13E-06
17309785	Sepp1	0.40	1.02E-08	2.69E-06
17362839	Ms4a10	0.40	7.11E-09	2.06E-06
17394063	Ada	0.39	3.86E-09	1.28E-06
17450354	Hsd17b13	0.39	6.22E-07	6.01E-05

17236393	Slc5a8	0.39	5.79E-08	9.98E-06
17337089	H2-Q1	0.38	9.27E-07	8.09E-05
17312567	Gpt	0.38	1.10E-05	5.74E-04
17404091	Fabp4	0.38	2.67E-04	6.10E-03
17458407	Abp1	0.38	2.29E-09	8.43E-07
17236182	Timp3	0.38	2.64E-07	3.08E-05
17345066	Mep1a	0.37	3.66E-09	1.24E-06
17484477	Prap1	0.37	6.08E-06	3.58E-04
17290163	Ccl28	0.37	2.05E-08	4.57E-06
17295386	Tmem171	0.37	2.21E-07	2.68E-05
17545899	S100g	0.37	1.04E-07	1.47E-05
17364440	Cyp2c69	0.37	3.13E-05	1.27E-03
17450838	Rnf212	0.37	8.96E-07	7.92E-05
17480044	Prss23	0.36	1.34E-08	3.29E-06
17330799	Gm5485	0.36	8.61E-10	4.01E-07
17400773	Fmo5	0.36	9.50E-08	1.40E-05
17425609	D630039A03Rik	0.36	1.77E-06	1.35E-04
17482897	Aqp8	0.36	9.90E-08	1.42E-05
17211405	Gsta3	0.36	2.33E-06	1.68E-04
17225210	Alpi	0.35	8.61E-08	1.30E-05
17277760	5430427M07Rik	0.35	1.03E-06	8.68E-05
17504572	Ces2g	0.34	2.11E-06	1.54E-04
17540378	Maob	0.34	9.45E-06	5.11E-04
17503926	Mt4	0.32	2.65E-05	1.12E-03
17475870	Sycn	0.32	2.40E-08	4.90E-06
17504477	Ces2a	0.31	1.38E-05	6.86E-04
17319707	Cyp2d26	0.31	2.03E-08	4.57E-06
17506532	Dpep1	0.31	5.52E-09	1.64E-06
17345989	Sult1c2	0.30	8.34E-08	1.27E-05
17322750	Abat	0.30	3.23E-08	6.14E-06
17409130	Gstm3	0.29	9.79E-09	2.63E-06
17376191	Tgm3	0.29	7.10E-10	3.43E-07
17504541	Ces2e	0.29	1.06E-10	1.12E-07
17504512	Ces2c	0.29	8.66E-09	2.40E-06
17329847	Osta	0.28	1.70E-09	7.20E-07
17516978	Fam55d	0.26	1.64E-08	3.81E-06
17526663	Fam55b	0.26	2.34E-06	1.69E-04
17252183	Eno3	0.26	9.24E-11	1.04E-07
17317442	Gsdmc2	0.25	7.91E-06	4.38E-04
17501250	Hpgd	0.25	4.17E-12	1.01E-08
17472114	Plbd1	0.24	5.61E-10	3.11E-07
17348833	Ttr	0.24	6.49E-10	3.23E-07
17402350	Fabp2	0.24	1.49E-10	1.40E-07
17507829	Defb37	0.24	2.19E-05	9.73E-04
17408336	Hsd3b3	0.22	1.04E-08	2.70E-06

17400862	Hmgcs2	0.22	4.82E-09	1.50E-06
17397750	Tm4sf4	0.20	4.46E-10	2.78E-07
17322075	Krt4	0.20	3.47E-07	3.81E-05
17408360	Hao2	0.17	1.30E-10	1.29E-07
17425233	Aldob	0.16	1.98E-09	7.77E-07
17359239	Cyp2c55	0.15	1.73E-11	2.93E-08
17360785	Pnliprp2	0.15	1.96E-09	7.77E-07

Supplementary Table 1: IL22 responsive genes in mouse colonoids. 859 genes are differentially expressed (q-value <= 0.01) after treatment with IL22. The data are sorted by decreasing values of the estimated expression fold change relative to control samples.

Supplementary Table 2

Transcript Cluster id	Gene	Fold change	p-value	q-value
17237978	Ddit3	9.01	8.95E-13	1.51E-08
17374807	Chac1	8.01	7.61E-11	1.52E-07
17392925	Trib3	7.47	1.57E-07	6.31E-05
17234423	Derl3	5.74	4.98E-09	4.38E-06
17504005	Herpud1	5.63	2.90E-12	2.45E-08
17397421	Slc7a11	4.81	1.08E-08	8.69E-06
17468143	Mthfd2	4.58	8.09E-11	1.52E-07
17313998	Creld2	4.07	1.20E-11	6.77E-08
17513641	Slc7a5	3.96	3.90E-11	9.42E-08
17277396	Jdp2	3.95	3.88E-09	4.11E-06
17303412	Acox2	3.69	1.68E-10	2.58E-07
17328625	Sdf2l1	3.32	9.04E-11	1.53E-07
17445440	Steap1	3.28	1.81E-08	1.02E-05
17370103	Grp78 (Hspa5)	3.27	3.06E-11	9.42E-08
17471484	Clec2e	3.25	1.32E-09	1.87E-06
17362453	Slc3a2	3.15	3.66E-11	9.42E-08
17356583	Syvn1	3.15	2.83E-11	9.42E-08
17503446	Gpt2	2.89	7.05E-05	6.44E-03
17314115	Adm2	2.88	2.10E-08	1.11E-05
17453124	Psph	2.78	1.40E-08	9.49E-06
17515335	Gm6484	2.77	7.24E-07	2.11E-04
17455401	Slc7a1	2.76	1.33E-08	9.45E-06
17280893	Dnajb9	2.76	4.27E-09	4.25E-06
17516564	Hyou1	2.75	1.80E-09	2.19E-06
17529549	Snhg5	2.70	1.34E-08	9.45E-06
17490878	Ppp1r15a	2.69	5.18E-09	4.38E-06
17407934	Gm129	2.62	1.05E-07	4.56E-05
17292634	Nfil3	2.44	1.12E-06	2.97E-04
17364521	Aldh18a1	2.41	1.59E-08	9.81E-06
17365014	Erlin1	2.36	1.46E-08	9.53E-06
17464718	Asns	2.33	1.81E-09	2.19E-06
17530782	Manf	2.26	1.68E-08	9.81E-06
17389093	2700007P21Rik	2.26	2.41E-06	5.22E-04
17545440	Tspyl2	2.26	1.21E-05	1.70E-03
17283056	Sel1l	2.23	2.05E-08	1.11E-05
17271861	C630004H02Rik	2.22	1.63E-08	9.81E-06
17231033	Atf3	2.22	3.38E-08	1.68E-05
17325619	Tmem39a	2.19	4.84E-09	4.38E-06
17315312	Soat2	2.18	7.66E-06	1.26E-03
17419389	Snhg12	2.15	2.75E-06	5.66E-04
17450091	5430416N02Rik	2.15	3.19E-08	1.63E-05
17300504	Pck2	2.15	2.78E-06	5.66E-04

17229556	Atf6	2.10	1.34E-07	5.54E-05
17402361	Sec24d	2.07	6.10E-07	1.84E-04
17388261	Atg13	2.07	1.28E-06	3.23E-04
17214857	Ccl20	2.04	6.29E-05	5.95E-03
17452552	Rhof	2.04	3.07E-07	1.08E-04
17433221	Slc25a33	2.02	1.47E-06	3.51E-04
17430861	Sesn2	2.00	8.96E-08	4.09E-05
17274249	Pdia6	1.96	3.50E-09	3.95E-06
17321318	Fkbp11	1.94	1.04E-05	1.59E-03
17490391	Atf5	1.93	1.05E-05	1.59E-03
17359008	March5	1.92	1.33E-06	3.31E-04
17235320	Reep6	1.88	2.22E-07	8.53E-05
17498271	Cars	1.88	1.47E-06	3.51E-04
17320928	Slc38a2	1.87	1.15E-08	8.88E-06
17448645	Nfxl1	1.86	8.11E-07	2.32E-04
17313495	Ccdc134	1.86	6.43E-06	1.10E-03
17407190	Hax1	1.85	2.04E-07	8.03E-05
17245902	Shmt2	1.84	9.42E-07	2.61E-04
17341132	Phf10	1.84	3.56E-08	1.72E-05
17313100	Atf4	1.84	1.27E-05	1.73E-03
17493571	Tsku	1.83	2.22E-06	4.94E-04
17302724	Dnajc3	1.83	4.50E-08	2.12E-05
17245511	Xpot	1.83	2.17E-06	4.89E-04
17277134	Acot2	1.83	3.46E-06	6.89E-04
17219963	Pppde1	1.82	1.05E-05	1.59E-03
17408273	Phgdh	1.78	1.11E-05	1.65E-03
17444481	Bhlha15	1.78	1.08E-04	8.88E-03
17489707	Cebpg	1.76	2.27E-06	4.98E-04
17379938	Cebpb	1.76	1.18E-05	1.68E-03
17478958	1810008I18Rik	1.75	4.31E-06	8.01E-04
17510114	Gdf15	1.74	2.31E-05	2.75E-03
17261425	Stc2	1.72	1.28E-05	1.73E-03
17219546	Slamf9	1.71	1.77E-05	2.20E-03
17500327	Eif4ebp1	1.70	2.46E-07	9.04E-05
17421550	Mthfr	1.70	1.25E-04	9.79E-03
17311602	Mtbp	1.70	8.06E-06	1.31E-03
17410809	Lmo4	1.69	2.75E-05	3.06E-03
17501250	Hpgd	1.69	2.28E-07	8.59E-05
17492114	Chd2	1.68	1.03E-06	2.80E-04
17530233	Srprb	1.68	5.60E-07	1.76E-04
17287206	Iars	1.68	3.52E-07	1.22E-04
17500172	Ppapdc1b	1.67	9.88E-08	4.40E-05
17482095	Nucb2	1.67	1.20E-07	5.08E-05
17483058	Gm20367	1.66	9.21E-05	7.91E-03
17260869	Slc1a4	1.65	1.32E-05	1.75E-03

17479875	Stard5	1.65	4.28E-06	8.01E-04
17440748	Ficd	1.65	8.05E-05	7.13E-03
17354036	Lars	1.64	4.48E-07	1.43E-04
17272745	Usp36	1.64	1.09E-05	1.63E-03
17324305	Dnajb11	1.63	1.04E-06	2.80E-04
17458771	Gars	1.63	4.16E-07	1.38E-04
17316021	Tars	1.63	4.08E-06	7.75E-04
17217440	Cyb5r1	1.62	1.92E-05	2.34E-03
17330967	Nfkbiz	1.62	2.66E-05	3.04E-03
17409328	Sars	1.62	3.06E-07	1.08E-04
17331533	Hspa13	1.62	1.23E-04	9.69E-03
17337984	Gtpbp2	1.61	1.64E-05	2.08E-03
17357168	Stx5a	1.60	1.16E-06	3.02E-04
17478998	H47	1.59	4.36E-07	1.42E-04
17350790	Prrc1	1.58	3.57E-06	6.95E-04
17261730	Rars	1.57	5.75E-07	1.77E-04
17529449	4922501C03Rik	1.57	7.99E-05	7.11E-03
17250734	2410006H16Rik	1.57	1.22E-06	3.13E-04
17521621	Amigo3	1.56	8.74E-05	7.58E-03
17346210	Ticam1	1.56	5.84E-06	1.03E-03
17418952	Yars	1.55	1.38E-05	1.79E-03
17496345	Nupr1	1.55	1.88E-06	4.31E-04
17220548	Eprs	1.55	1.36E-06	3.34E-04
17509282	Cdkn2aip	1.54	2.52E-06	5.39E-04
17262300	Sqstm1	1.54	1.18E-05	1.68E-03
17317203	Derl1	1.54	1.73E-06	4.07E-04
17357126	Snhg1	1.54	8.30E-05	7.31E-03
17471062	Tapbpl	1.54	8.34E-06	1.34E-03
17508722	Leprotl1	1.54	3.12E-06	6.29E-04
17280842	Ifrd1	1.53	6.52E-06	1.10E-03
17314061	Trabd	1.53	3.52E-06	6.92E-04
17229948	Dusp23	1.53	3.74E-05	3.83E-03
17326428	Cpox	1.53	4.50E-05	4.45E-03
17445426	Steap2	1.52	7.08E-05	6.44E-03
17310673	Ank	1.52	3.74E-05	3.83E-03
17361090	BC021614	1.52	2.69E-05	3.04E-03
17411751	Trp53inp1	1.52	6.92E-06	1.16E-03
17328132	Txndc11	1.52	2.46E-05	2.90E-03
17535298	Mtm1	1.51	6.55E-05	6.06E-03
17283930	Wars	1.51	1.84E-06	4.27E-04
17255387	Slc35b1	1.51	9.42E-07	2.61E-04
17348435	Cables1	1.51	1.80E-05	2.22E-03
17330654	BC016579	1.50	2.52E-05	2.96E-03
17311649	Gm20184	1.50	3.90E-06	7.49E-04
17245850	Mars	1.49	6.53E-06	1.10E-03

17423674	1810074P20Rik	1.49	5.66E-06	1.02E-03
17274123	Nbas	1.49	5.78E-05	5.56E-03
17356099	Cdk2ap2	1.49	1.31E-05	1.75E-03
17477897	Bcat2	1.48	2.61E-06	5.51E-04
17343744	Slc39a7	1.47	4.67E-06	8.49E-04
17268356	Nfe2l1	1.47	1.15E-05	1.68E-03
17431394	Clic4	1.47	1.05E-05	1.59E-03
17243738	Hsp90b1	1.45	1.26E-05	1.73E-03
17505851	Nudt7	1.45	1.19E-04	9.48E-03
17464803	Ica1	1.45	1.68E-05	2.11E-03
17447544	Wfs1	1.45	1.00E-04	8.35E-03
17295482	Mtap1b	1.44	1.26E-04	9.84E-03
17439064	Uso1	1.44	3.49E-05	3.69E-03
17241137	Unc5b	1.43	1.02E-04	8.47E-03
17246967	Xbp1	1.43	1.33E-05	1.75E-03
17377092	Sec23b	1.42	6.48E-05	6.02E-03
17435867	Tmem214	1.42	9.02E-06	1.43E-03
17397968	Arhgef26	1.41	9.58E-05	8.16E-03
17439741	Aff1	1.40	1.33E-05	1.75E-03
17530322	Uba5	1.39	3.40E-05	3.64E-03
17297100	Kctd6	1.39	2.67E-05	3.04E-03
17353420	Fam13b	1.38	2.73E-05	3.06E-03
17511183	Calr	1.38	1.27E-05	1.73E-03
17376649	Crls1	1.38	9.75E-05	8.16E-03
17350356	Eif1a	1.37	1.24E-05	1.73E-03
17355009	Nars	1.37	3.42E-05	3.64E-03
17344114	1110038B12Rik	1.37	5.30E-05	5.19E-03
17492051	Arrdc4	1.36	3.29E-05	3.57E-03
17221186	Snhg6	1.36	4.23E-05	4.21E-03
17305167	Ghitm	1.35	3.52E-05	3.70E-03
17331044	Tfg	1.35	2.60E-05	3.03E-03
17439029	Areg	1.34	2.67E-05	3.04E-03
17379864	1500012F01Rik	1.34	2.80E-05	3.09E-03
17334365	Zfp598	1.34	1.15E-04	9.22E-03
17431496	Pnrc2	1.34	9.65E-05	8.16E-03
17383188	Surf4	1.33	7.49E-05	6.74E-03
17358119	Zfand5	1.32	6.75E-05	6.20E-03
17410376	Ostc	1.32	7.16E-05	6.48E-03
17400599	Txnip	1.31	2.95E-05	3.24E-03
17272287	Srp68	1.31	1.21E-04	9.54E-03
17452405	Atp2a2	1.31	1.10E-04	8.96E-03
17514435	Casp4	1.30	9.75E-05	8.16E-03
17225472	Zfp330	1.28	1.07E-04	8.84E-03
17367095	Tmem236	0.75	5.78E-05	5.56E-03
17230550	Lbr	0.74	4.02E-05	4.02E-03

17367916	Lrrc26	0.73	1.11E-04	8.96E-03
17313087	Mgat3	0.73	3.57E-05	3.70E-03
17295233	Hmgcr	0.73	1.15E-05	1.68E-03
17248476	Pank3	0.73	4.95E-05	4.87E-03
17440257	Pcgf3	0.72	2.70E-05	3.04E-03
17274889	Gdap10	0.72	1.10E-04	8.96E-03
17429632	Mfsd2a	0.72	9.17E-05	7.91E-03
17409463	Fam102b	0.72	1.16E-04	9.28E-03
17515315	Ldlr	0.71	2.30E-05	2.75E-03
17311807	Sqle	0.71	8.61E-05	7.51E-03
17236882	Dusp6	0.71	1.69E-05	2.11E-03
17229466	Hsd17b7	0.71	6.42E-05	6.02E-03
17513311	2310061C15Rik	0.71	3.97E-05	4.02E-03
17350740	Lmnb1	0.70	3.82E-05	3.89E-03
17356314	Mrpl11	0.70	1.50E-05	1.92E-03
17447831	Fgfbp1	0.69	6.07E-06	1.06E-03
17290173	Hmgcs1	0.69	4.54E-06	8.34E-04
17269521	Acly	0.69	6.30E-05	5.95E-03
17460933	Lsm3	0.68	7.72E-05	6.91E-03
17340943	Mrpl18	0.68	1.18E-05	1.68E-03
17525894	Sorl1	0.68	5.92E-05	5.65E-03
17251607	Trappc1	0.68	5.40E-05	5.25E-03
17512868	Chst4	0.68	8.43E-05	7.39E-03
17323828	B3gnt5	0.67	3.57E-05	3.70E-03
17416325	Dhcr24	0.67	2.64E-06	5.52E-04
17429057	Ptprf	0.67	5.78E-06	1.03E-03
17481102	Rrm1	0.67	1.14E-04	9.21E-03
17510707	Lsm6	0.66	3.03E-05	3.30E-03
17511377	Neto2	0.66	1.84E-05	2.25E-03
17514799	2200002K05Rik	0.66	1.96E-05	2.37E-03
17326510	Pros1	0.66	8.69E-06	1.39E-03
17221633	Mcm3	0.64	6.48E-05	6.02E-03
17328432	Mcm4	0.63	9.37E-06	1.47E-03
17442719	Aacs	0.63	3.42E-05	3.64E-03
17338701	Chaf1a	0.60	1.45E-05	1.88E-03
17402558	Elovl6	0.59	4.01E-05	4.02E-03
17539979	Glod5	0.57	7.16E-06	1.19E-03
17273348	Fasn	0.57	6.73E-07	2.00E-04
17394694	B4galt5	0.56	3.91E-07	1.32E-04
17357815	Fam111a	0.50	9.63E-05	8.16E-03

Supplementary Table 2: Tunicamycin responsive genes in mouse colonoids. 217 genes are differentially expressed (q-value <= 0.01) after treatment with tunicamycin. The data are sorted by decreasing values of the estimated expression fold change relative to control samples.

Supplementary Table 3

Transcript cluster id	Gene	Fold change	p-value	q-value
17459744	Reg3b	155.14	1.10E-14	1.86E-10
17467973	Reg3g	51.76	4.41E-12	5.74E-09
17491193	Saa3	30.07	6.28E-10	1.52E-07
17399823	S100a8	17.33	3.25E-12	5.00E-09
17234423	Derl3	13.61	5.43E-11	2.81E-08
17392925	Trib3	13.21	1.04E-08	1.01E-06
17374807	Chac1	12.86	7.35E-12	8.88E-09
17346150	Lrg1	12.36	1.38E-11	1.30E-08
17237978	Ddit3	11.65	2.51E-13	7.08E-10
17505478	Tat	10.67	4.37E-12	5.74E-09
17315743	Osmr	9.63	1.99E-10	7.66E-08
17504005	Herpud1	8.80	2.06E-13	7.08E-10
17253707	Nos2	8.40	1.93E-09	3.13E-07
17397421	Slc7a11	7.40	7.25E-10	1.63E-07
17313998	Creld2	7.24	2.31E-13	7.08E-10
17314115	Adm2	7.08	2.06E-11	1.83E-08
17326062	Retnlb	6.90	6.90E-10	1.58E-07
17401261	Bcl2l15	6.09	7.85E-09	8.05E-07
17468143	Mthfd2	6.07	1.16E-11	1.22E-08
17214857	Ccl20	5.86	5.67E-09	6.47E-07
17356583	Syvn1	5.67	2.46E-13	7.08E-10
17513641	Slc7a5	5.64	2.84E-12	4.80E-09
17490878	Ppp1r15a	5.53	1.06E-11	1.20E-08
17407363	S100a9	5.50	1.69E-09	2.91E-07
17328625	Sdf2l1	5.50	1.63E-12	3.45E-09
17321318	Fkbp11	5.33	5.30E-10	1.42E-07
17438955	Cxcl5	5.31	5.15E-11	2.81E-08
17277396	Jdp2	5.10	5.77E-10	1.48E-07
17254289	Gm11428	5.00	2.52E-08	1.85E-06
17503446	Gpt2	4.91	1.37E-06	3.99E-05
17402361	Sec24d	4.89	1.18E-10	5.13E-08
17292634	Nfil3	4.78	2.53E-09	3.70E-07
17385374	Nr4a2	4.75	5.58E-09	6.42E-07
17516564	Hyou1	4.74	1.38E-11	1.30E-08
17515335	Gm6484	4.71	7.98E-09	8.09E-07
17362453	Slc3a2	4.71	1.19E-12	2.86E-09
17471484	Clec2e	4.68	6.25E-11	3.07E-08
17280893	Dnajb9	4.67	3.84E-11	2.40E-08
17410905	AI747448	4.66	6.24E-10	1.52E-07
17231033	Atf3	4.53	2.61E-11	2.11E-08
17370103	Grp78 (Hspa5)	4.51	1.99E-12	3.73E-09
17313495	Ccdc134	4.46	5.25E-10	1.42E-07

17455401	Slc7a1	4.36	2.08E-10	7.83E-08
17445440	Steap1	4.29	1.92E-09	3.13E-07
17271861	C630004H02Rik	4.20	2.21E-11	1.87E-08
17286190	Prl2a1	4.07	1.38E-06	4.01E-05
17283056	Sel1l	3.96	4.75E-11	2.77E-08
17229556	Atf6	3.93	1.51E-10	6.06E-08
17433221	Slc25a33	3.87	1.27E-09	2.47E-07
17444481	Bhlha15	3.84	2.00E-08	1.59E-06
17521621	Amigo3	3.83	9.63E-10	1.98E-07
17383892	Lcn2	3.81	9.53E-09	9.43E-07
17344765	Trim15	3.77	6.36E-11	3.07E-08
17452552	Rhof	3.66	4.26E-10	1.26E-07
17303412	Acox2	3.65	1.82E-10	7.17E-08
17318089	Ly6c1	3.62	1.02E-07	5.45E-06
17455954	Tac1	3.55	5.03E-08	3.17E-06
17448645	Nfxl1	3.54	3.52E-10	1.10E-07
17453124	Psph	3.49	1.50E-09	2.74E-07
17395079	Zbp1	3.48	9.02E-10	1.91E-07
17388261	Atg13	3.47	3.92E-09	5.14E-07
17364521	Aldh18a1	3.44	3.48E-10	1.10E-07
17529549	Snhg5	3.32	1.66E-09	2.89E-07
17464718	Asns	3.30	3.59E-11	2.40E-08
17306098	Rnase1	3.25	2.35E-08	1.77E-06
17365014	Erlin1	3.23	4.39E-10	1.26E-07
17325619	Tmem39a	3.21	5.49E-11	2.81E-08
17378827	Lbp	3.21	2.29E-08	1.76E-06
17254166	Slfn2	3.16	1.09E-06	3.32E-05
17390738	Duox2	3.16	1.47E-08	1.29E-06
17530233	Srprb	3.12	1.02E-10	4.54E-08
17401345	Ppm1j	3.11	6.90E-10	1.58E-07
17408885	Chi3l7	3.08	1.02E-05	1.88E-04
17269404	Krt16	3.06	4.81E-06	1.06E-04
17416212	1700024P16Rik	3.05	1.87E-08	1.51E-06
17450091	5430416N02Rik	3.01	5.79E-10	1.48E-07
17526836	Tex12	3.00	1.69E-05	2.84E-04
17407934	Gm129	2.92	3.29E-08	2.35E-06
17482095	Nucb2	2.92	3.28E-11	2.40E-08
17331533	Hspa13	2.92	4.08E-08	2.77E-06
17530782	Manf	2.91	8.46E-10	1.81E-07
17420539	Pla2g2a	2.86	1.31E-06	3.83E-05
17430861	Sesn2	2.86	9.21E-10	1.92E-07
17315312	Soat2	2.86	3.60E-07	1.46E-05
17288898	Lysmd3	2.84	7.92E-08	4.44E-06
17226447	Nckap5	2.84	2.35E-06	5.97E-05
17545440	Tspyl2	2.81	1.15E-06	3.43E-05

17238172	Rdh16	2.79	3.06E-06	7.37E-05
17471062	Tapbpl	2.79	8.22E-10	1.78E-07
17302724	Dnajc3	2.78	1.28E-10	5.40E-08
17313000	Kdelr3	2.76	3.77E-09	4.98E-07
17254047	Ccl7	2.75	4.47E-07	1.71E-05
17399374	Muc1	2.75	1.58E-07	7.68E-06
17274249	Pdia6	2.74	3.75E-11	2.40E-08
17330967	Nfkbiz	2.74	1.29E-08	1.18E-06
17464480	3010003L21Rik	2.72	2.07E-05	3.33E-04
17464580	Bet1	2.70	3.88E-07	1.56E-05
17308099	Adam28	2.69	5.00E-06	1.09E-04
17245511	Xpot	2.69	1.09E-08	1.04E-06
17261608	Gabrp	2.69	2.88E-08	2.09E-06
17393789	Tgm2	2.69	4.37E-09	5.60E-07
17448050	Ccdc149	2.68	1.66E-07	8.01E-06
17274123	Nbas	2.67	4.82E-09	5.92E-07
17350790	Prrc1	2.66	9.72E-10	1.98E-07
17254176	Slfn4	2.65	2.22E-06	5.73E-05
17333344	Snora20	2.64	3.47E-05	4.96E-04
17419389	Snhg12	2.64	2.41E-07	1.07E-05
17320928	Slc38a2	2.61	9.71E-11	4.44E-08
17237851	Avil	2.61	4.82E-08	3.08E-06
17497525	Adam8	2.60	8.53E-08	4.67E-06
17330654	BC016579	2.60	3.40E-09	4.70E-07
17396035	Zbtb10	2.59	2.80E-08	2.04E-06
17404432	Fndc3b	2.59	2.38E-09	3.59E-07
17500327	Eif4ebp1	2.58	4.33E-10	1.26E-07
17438987	Cxcl1	2.56	7.86E-08	4.44E-06
17438975	Cxcl3	2.56	3.07E-04	2.72E-03
17300504	Pck2	2.54	3.57E-07	1.45E-05
17495821	Cdr2	2.53	4.53E-07	1.73E-05
17530322	Uba5	2.53	6.40E-10	1.52E-07
17257165	Arf2	2.52	6.53E-09	7.13E-07
17502573	Hmox1	2.50	4.36E-09	5.60E-07
17514592	Mmp7	2.50	1.75E-06	4.82E-05
17357168	Stx5a	2.50	8.19E-10	1.78E-07
17344774	Trim40	2.49	2.97E-07	1.27E-05
17439164	Sep-11	2.48	2.01E-08	1.59E-06
17239546	D10Bwg1379e	2.48	4.13E-08	2.77E-06
17355619	Adnp2	2.48	3.12E-06	7.46E-05
17407190	Hax1	2.45	3.28E-09	4.58E-07
17359008	Mar-05	2.45	4.58E-08	2.98E-06
17389093	2700007P21Rik	2.45	9.31E-07	2.93E-05
17324305	Dnajb11	2.44	1.50E-09	2.74E-07
17498271	Cars	2.44	3.76E-08	2.61E-06

17535298	Mtm1	2.43	2.91E-08	2.10E-06
17283930	Wars	2.42	5.07E-10	1.42E-07
17487489	Pvr	2.42	6.18E-09	6.90E-07
17438995	Cxcl2	2.42	5.90E-08	3.58E-06
17353650	Ecscr	2.41	2.32E-09	3.59E-07
17280561	Dus4l	2.40	5.89E-04	4.51E-03
17316021	Tars	2.40	8.05E-09	8.11E-07
17262300	Sqstm1	2.39	7.97E-09	8.09E-07
17490912	Fut2	2.38	7.18E-09	7.58E-07
17410332	Ccdc109b	2.38	4.82E-07	1.80E-05
17241137	Unc5b	2.36	1.34E-08	1.21E-06
17545764	Ppef1	2.36	3.41E-05	4.90E-04
17218186	Fam129a	2.36	4.49E-08	2.93E-06
17354463	Mar-03	2.35	7.70E-09	7.94E-07
17539548	Piga	2.35	6.86E-06	1.36E-04
17394153	Slpi	2.35	2.09E-09	3.33E-07
17506230	6430548M08Rik	2.34	4.25E-08	2.81E-06
17459425	Eif2ak3	2.34	4.78E-08	3.08E-06
17314061	Trabd	2.33	2.21E-09	3.50E-07
17478998	H47	2.33	6.33E-10	1.52E-07
17482943	Il4ra	2.32	1.21E-07	6.32E-06
17269333	Krt36	2.31	6.32E-06	1.30E-04
17328132	Txndc11	2.31	1.75E-08	1.47E-06
17435221	Rint1	2.31	1.23E-08	1.14E-06
17439131	Stbd1	2.30	1.32E-05	2.32E-04
17452054	Oas2	2.30	2.64E-06	6.52E-05
17397426	Ccrn4l	2.30	1.59E-07	7.72E-06
17211795	Arid5a	2.29	9.75E-08	5.23E-06
17529322	Ibtk	2.29	2.76E-09	3.92E-07
17548166	LOC100862491	2.29	1.02E-03	7.02E-03
17522219	Nme6	2.28	1.91E-07	8.96E-06
17485309	Tssc4	2.28	1.31E-06	3.83E-05
17330742	Plcxd2	2.27	2.16E-06	5.63E-05
17355984	Chka	2.27	5.76E-08	3.52E-06
17353663	Tmem173	2.27	3.14E-08	2.25E-06
17500172	Ppapdc1b	2.26	6.02E-10	1.52E-07
17348435	Cables1	2.26	1.43E-08	1.26E-06
17377092	Sec23b	2.26	1.25E-08	1.15E-06
17440748	Ficd	2.25	6.96E-07	2.38E-05
17530802	Rbm15b	2.24	1.22E-06	3.62E-05
17509565	Anxa10	2.24	2.37E-04	2.23E-03
17341132	Phf10	2.24	1.60E-09	2.86E-07
17311649	Gm20184	2.24	2.31E-09	3.59E-07
17523332	Nktr	2.24	1.91E-07	8.95E-06
17410809	Lmo4	2.22	4.14E-07	1.63E-05

17313100	Atf4	2.21	8.76E-07	2.81E-05
17236196	1810014B01Rik	2.21	1.57E-05	2.69E-04
17236091	AI597468	2.21	4.18E-06	9.42E-05
17228871	AI848100	2.19	4.68E-09	5.87E-07
17439021	Ereg	2.19	2.65E-07	1.17E-05
17345315	Mad2l1bp	2.19	5.32E-08	3.29E-06
17280842	Ifrd1	2.18	1.12E-08	1.06E-06
17467768	Sh2d6	2.18	3.08E-04	2.72E-03
17338637	Zfp959	2.18	1.30E-04	1.40E-03
17435867	Tmem214	2.18	1.71E-09	2.91E-07
17458771	Gars	2.18	2.60E-09	3.73E-07
17269391	Krt14	2.18	2.36E-06	5.98E-05
17402519	Tifa	2.18	1.49E-04	1.56E-03
17260668	Grb10	2.18	1.70E-06	4.73E-05
17428253	Slc5a9	2.17	6.02E-07	2.13E-05
17287206	Iars	2.17	4.61E-09	5.82E-07
17439064	Uso1	2.16	1.49E-08	1.30E-06
17213666	Klf7	2.16	9.19E-05	1.07E-03
17431234	Cnksr1	2.16	8.49E-09	8.45E-07
17364948	Slc25a28	2.16	2.19E-08	1.70E-06
17458592	Hoxa1	2.16	1.76E-06	4.84E-05
17337984	Gtpbp2	2.16	1.30E-07	6.61E-06
17400023	Them4	2.16	1.75E-05	2.91E-04
17219963	Pppde1	2.15	8.83E-07	2.82E-05
17290069	Pelo	2.15	5.41E-07	1.98E-05
17515371	2310047B19Rik	2.15	4.42E-05	5.97E-04
17431394	Clic4	2.15	7.13E-09	7.58E-07
17343744	Slc39a7	2.14	3.47E-09	4.73E-07
17245902	Shmt2	2.14	9.05E-08	4.89E-06
17267504	Lpo	2.14	1.27E-06	3.74E-05
17492114	Chd2	2.14	1.85E-08	1.50E-06
17328153	Zc3h7a	2.14	2.98E-06	7.22E-05
17515074	Icam1	2.14	3.58E-07	1.45E-05
17347903	Mcfd2	2.14	8.30E-08	4.59E-06
17342674	Mir3083	2.13	9.78E-04	6.78E-03
17245850	Mars	2.13	8.35E-09	8.35E-07
17317203	Derl1	2.13	4.55E-09	5.79E-07
17272745	Usp36	2.13	1.41E-07	6.94E-06
17376649	Crls1	2.12	1.62E-08	1.39E-06
17473810	Zfp324	2.12	9.14E-05	1.06E-03
17478958	1810008I18Rik	2.12	2.12E-07	9.73E-06
17483058	Gm20367	2.11	2.34E-06	5.95E-05
17533713	Timp1	2.11	1.16E-04	1.28E-03
17357584	Vps37c	2.10	7.20E-07	2.44E-05
17525492	A630095E13Rik	2.10	2.45E-05	3.79E-04

17397462	Rab33b	2.10	1.09E-07	5.80E-06
17391554	Il1a	2.10	2.13E-04	2.06E-03
17490391	Atf5	2.10	3.21E-06	7.62E-05
17493571	Tsku	2.09	2.78E-07	1.20E-05
17409633	Slc30a7	2.09	2.46E-06	6.18E-05
17235037	Arid3a	2.09	4.15E-05	5.69E-04
17465906	Creb3l2	2.09	6.53E-07	2.28E-05
17408273	Phgdh	2.08	9.57E-07	3.00E-05
17492051	Arrdc4	2.08	3.53E-09	4.77E-07
17354036	Lars	2.08	6.80E-09	7.28E-07
17268356	Nfe2l1	2.07	1.69E-08	1.43E-06
17356099	Cdk2ap2	2.06	2.42E-08	1.80E-06
17311602	Mtbp	2.06	3.35E-07	1.39E-05
17421550	Mthfr	2.06	7.07E-06	1.39E-04
17447544	Wfs1	2.06	1.28E-07	6.61E-06
17297100	Kctd6	2.06	7.62E-09	7.91E-07
17514435	Casp4	2.06	3.42E-09	4.70E-07
17277134	Acot2	2.06	5.45E-07	1.99E-05
17423674	1810074P20Rik	2.05	1.14E-08	1.07E-06
17489707	Cebpg	2.05	1.84E-07	8.65E-06
17383188	Surf4	2.05	5.51E-09	6.39E-07
17363177	Tle4	2.05	4.00E-07	1.60E-05
17272619	Socs3	2.05	1.30E-06	3.81E-05
17479317	Isg20	2.04	2.94E-07	1.26E-05
17319806	Arfgap3	2.04	7.63E-08	4.39E-06
17317266	Fbxo32	2.04	3.09E-06	7.42E-05
17217440	Cyb5r1	2.04	4.02E-07	1.60E-05
17286707	Gcnt2	2.03	7.25E-07	2.45E-05
17463443	Clec2h	2.03	2.58E-09	3.73E-07
17360097	Cnnm2	2.03	1.12E-05	2.04E-04
17357771	Osbp	2.03	4.98E-08	3.15E-06
17526195	Hinfp	2.03	1.53E-06	4.35E-05
17379938	Cebpb	2.02	1.26E-06	3.72E-05
17464803	Ica1	2.02	2.17E-08	1.69E-06
17545785	Cdkl5	2.02	1.62E-06	4.54E-05
17261730	Rars	2.02	5.03E-09	6.04E-07
17214759	Rhbdd1	2.02	5.48E-07	1.99E-05
17418952	Yars	2.01	1.17E-07	6.13E-06
17508722	Leprotl1	2.01	1.85E-08	1.50E-06
17344132	Hspa1a	2.01	9.57E-07	3.00E-05
17243544	Aldh1l2	2.01	7.74E-06	1.49E-04
17439741	Aff1	2.01	6.66E-09	7.18E-07
17348791	Dsg3	2.00	4.74E-08	3.06E-06
17264835	Cd68	2.00	4.55E-07	1.73E-05
17359113	O3far1	2.00	3.02E-07	1.29E-05

17512895	Il34	1.99	1.83E-05	3.03E-04
17319565	Pppde2	1.99	7.17E-08	4.19E-06
17410376	Ostc	1.99	5.70E-09	6.47E-07
17469754	Cidec	1.99	7.30E-07	2.45E-05
17521194	Dusp7	1.98	5.04E-07	1.88E-05
17482133	Tmc5	1.97	7.67E-07	2.53E-05
17361959	Zfpl1	1.97	6.41E-07	2.26E-05
17297125	Ptprg	1.97	1.97E-06	5.22E-05
17518526	Clpx	1.97	5.17E-08	3.22E-06
17461433	Edem1	1.97	1.37E-08	1.22E-06
17470105	Csgalnact2	1.97	5.78E-05	7.30E-04
17358119	Zfand5	1.97	7.54E-09	7.87E-07
17520073	Nt5e	1.97	2.36E-08	1.77E-06
17255387	Slc35b1	1.96	4.87E-09	5.92E-07
17437803	N4bp2	1.96	8.07E-08	4.50E-06
17491941	Aldh1a3	1.96	1.68E-08	1.43E-06
17356490	Rela	1.96	1.34E-08	1.21E-06
17483048	Apobr	1.96	4.68E-05	6.22E-04
17320189	Alg12	1.96	1.71E-06	4.74E-05
17251527	Per1	1.96	1.73E-07	8.24E-06
17326428	Cpox	1.96	4.60E-07	1.75E-05
17266911	Mmp28	1.96	3.47E-05	4.96E-04
17474974	Plaur	1.96	7.22E-09	7.58E-07
17356356	Rin1	1.95	1.02E-06	3.16E-05
17255232	Lrrc59	1.95	5.33E-09	6.26E-07
17538171	Mid2	1.95	2.77E-06	6.80E-05
17267393	Dhx40	1.95	8.19E-07	2.65E-05
17426944	Fam154a	1.95	3.21E-06	7.62E-05
17326527	Cggbp1	1.95	2.67E-07	1.17E-05
17377753	H13	1.94	1.37E-08	1.22E-06
17220548	Eprs	1.94	1.51E-08	1.30E-06
17327830	Fam100a	1.93	1.55E-04	1.61E-03
17358214	Klf9	1.93	1.25E-07	6.50E-06
17384457	Stom	1.92	1.70E-08	1.43E-06
17411751	Trp53inp1	1.92	6.45E-08	3.84E-06
17437213	Cd38	1.92	5.32E-08	3.29E-06
17285559	Elmo1	1.91	5.46E-06	1.17E-04
17482041	Calcb	1.91	3.35E-04	2.90E-03
17531701	Gm20317	1.91	3.84E-04	3.22E-03
17464128	Golt1b	1.91	2.07E-08	1.63E-06
17355092	Lman1	1.91	4.91E-08	3.12E-06
17496345	Nupr1	1.90	3.05E-08	2.19E-06
17268066	Gm11545	1.90	8.09E-07	2.63E-05
17285204	Ero1lb	1.90	6.49E-06	1.32E-04
17292891	Pdlim7	1.90	1.39E-07	6.94E-06

17351414	Pmaip1	1.90	1.69E-07	8.08E-06
17230102	Ifi202b	1.90	1.83E-04	1.82E-03
17318070	2010109I03Rik	1.89	1.21E-08	1.13E-06
17260869	Slc1a4	1.89	1.25E-06	3.69E-05
17497687	Bet1l	1.89	4.33E-07	1.69E-05
17228624	Rasal2	1.89	3.98E-06	9.01E-05
17512088	Slc38a7	1.88	3.63E-05	5.11E-04
17410204	Alpk1	1.88	4.86E-06	1.06E-04
17458682	Creb5	1.88	6.70E-06	1.35E-04
17275351	Scfd1	1.88	2.40E-06	6.06E-05
17475523	BC024978	1.88	1.12E-05	2.04E-04
17476199	Zfp790	1.88	1.46E-05	2.52E-04
17504074	Cpne2	1.88	1.92E-08	1.54E-06
17528405	Rab8b	1.88	6.88E-05	8.35E-04
17521603	Gmppb	1.88	3.32E-07	1.38E-05
17354004	Yipf5	1.88	5.14E-08	3.21E-06
17242785	Sbno2	1.87	2.31E-07	1.04E-05
17433265	Car6	1.87	3.59E-05	5.07E-04
17350356	Eif1a	1.87	9.99E-09	9.82E-07
17519875	Mei4	1.87	4.57E-05	6.12E-04
17410617	Dapp1	1.87	4.38E-05	5.94E-04
17399883	Sprr2h	1.87	6.43E-04	4.85E-03
17391997	Gpcpd1	1.87	8.13E-08	4.52E-06
17500523	Tmem66	1.87	1.41E-07	6.94E-06
17397663	Spg20	1.86	1.35E-05	2.37E-04
17243617	Ckap4	1.86	9.86E-08	5.28E-06
17265748	Aspa	1.86	7.06E-06	1.39E-04
17507321	Efnb2	1.86	5.15E-08	3.21E-06
17298312	Glt8d1	1.86	6.24E-06	1.29E-04
17334365	Zfp598	1.86	6.53E-08	3.87E-06
17499962	Plat	1.86	1.38E-06	4.01E-05
17300396	Ngdn	1.86	1.27E-06	3.74E-05
17248196	Asb3	1.86	3.61E-05	5.10E-04
17345613	Ubr2	1.85	1.82E-06	4.94E-05
17511183	Calr	1.85	1.51E-08	1.30E-06
17306906	Ripk3	1.85	4.44E-08	2.92E-06
17278073	Golga5	1.85	4.15E-06	9.39E-05
17277270	Lin52	1.85	6.78E-04	5.03E-03
17246163	Esyt1	1.85	9.39E-06	1.75E-04
17409328	Sars	1.85	2.24E-08	1.72E-06
17383013	Sec16a	1.85	1.58E-07	7.68E-06
17365863	A630007B06Rik	1.85	2.97E-07	1.27E-05
17410880	Clca4	1.84	3.87E-08	2.67E-06
17480018	Ctsc	1.84	4.48E-08	2.93E-06
17241032	Ddit4	1.84	2.36E-05	3.69E-04

17399386	Krtcap2	1.84	3.38E-07	1.40E-05
17505762	Zfp1	1.84	5.63E-05	7.14E-04
17447803	Fbxl5	1.84	1.90E-06	5.12E-05
17346210	Ticam1	1.83	2.28E-07	1.03E-05
17261425	Stc2	1.83	4.53E-06	1.01E-04
17306396	Jub	1.83	1.48E-05	2.55E-04
17428032	Zyg11b	1.83	4.44E-07	1.71E-05
17439622	Agpat9	1.82	1.55E-07	7.59E-06
17246967	Xbp1	1.82	6.03E-08	3.64E-06
17511112	Ccdc130	1.82	2.81E-05	4.22E-04
17259026	Card14	1.82	1.76E-06	4.83E-05
17229948	Dusp23	1.81	1.41E-06	4.06E-05
17325109	Itgb5	1.81	2.60E-06	6.44E-05
17347163	Xdh	1.81	2.34E-06	5.95E-05
17330058	Pdia5	1.81	1.86E-05	3.06E-04
17397968	Arhgef26	1.81	4.42E-07	1.70E-05
17461677	Jagn1	1.81	1.67E-05	2.81E-04
17425151	Alg2	1.81	3.22E-07	1.35E-05
17383216	Slc2a6	1.81	8.44E-04	6.01E-03
17446775	Preb	1.80	1.79E-05	2.97E-04
17483733	Bag3	1.80	2.45E-08	1.81E-06
17477897	Bcat2	1.80	3.51E-08	2.47E-06
17254295	Expi	1.80	2.77E-05	4.18E-04
17509282	Cdkn2aip	1.80	1.12E-07	5.90E-06
17221792	Fam135a	1.80	3.11E-07	1.31E-05
17471324	Itfg2	1.79	9.69E-06	1.80E-04
17384753	Golga1	1.79	3.77E-07	1.52E-05
17516546	Dpagt1	1.79	9.28E-08	5.00E-06
17282664	Pgf	1.79	1.09E-04	1.21E-03
17395777	Ptk6	1.79	4.53E-04	3.68E-03
17250744	Snord65	1.79	1.51E-04	1.58E-03
17358797	Fas	1.79	1.78E-04	1.78E-03
17510114	Gdf15	1.79	1.50E-05	2.58E-04
17384578	Pdcl	1.79	3.27E-06	7.71E-05
17390810	Gatm	1.79	5.75E-04	4.43E-03
17370596	Gm19922	1.79	4.86E-05	6.38E-04
17351141	1500015A07Rik	1.79	6.86E-06	1.36E-04
17468730	Sec61a1	1.79	7.79E-08	4.44E-06
17250734	2410006H16Rik	1.78	8.40E-08	4.63E-06
17230111	Ifi205	1.78	7.04E-06	1.38E-04
17270567	Gosr2	1.78	1.04E-06	3.19E-05
17351681	Mex3c	1.78	2.01E-07	9.33E-06
17461554	Mtmr14	1.78	7.20E-07	2.44E-05
17525075	Herpud2	1.78	5.08E-07	1.88E-05
17414416	Dnajc25	1.78	3.49E-05	4.97E-04

17300591	Irf9	1.78	2.37E-05	3.70E-04
17523852	Birc2	1.78	3.95E-06	8.97E-05
17427312	Jun	1.78	3.17E-05	4.63E-04
17212138	Map4k4	1.77	7.13E-07	2.43E-05
17400966	Gdap2	1.77	2.50E-06	6.25E-05
17329433	Bcl6	1.77	9.20E-07	2.91E-05
17229166	Blzf1	1.77	3.68E-06	8.47E-05
17515886	Srpr	1.77	2.26E-07	1.03E-05
17523095	Exog	1.77	9.95E-05	1.13E-03
17336725	Dom3z	1.77	7.59E-07	2.53E-05
17275882	Mgat2	1.77	3.75E-06	8.59E-05
17357126	Snhg1	1.77	6.36E-06	1.30E-04
17317046	Tnfrsf11b	1.76	1.29E-07	6.61E-06
17268380	Cdk5rap3	1.76	2.24E-05	3.53E-04
17449585	Btc	1.76	7.01E-06	1.38E-04
17359520	Pi4k2a	1.76	1.01E-04	1.15E-03
17410845	Clca1	1.76	3.57E-04	3.05E-03
17430576	Tinagl1	1.76	6.57E-07	2.29E-05
17273240	Pycr1	1.76	2.08E-05	3.33E-04
17305813	Atg14	1.76	9.64E-06	1.80E-04
17418540	Stk40	1.75	1.74E-06	4.80E-05
17418357	Yrdc	1.75	6.93E-05	8.41E-04
17426348	Tnfsf15	1.75	6.24E-04	4.72E-03
17473765	Zfp110	1.75	4.33E-05	5.89E-04
17400799	Sec22b	1.75	6.32E-06	1.30E-04
17244476	Cradd	1.75	8.78E-06	1.65E-04
17445426	Steap2	1.75	4.87E-06	1.06E-04
17406031	Rapgef2	1.75	7.36E-06	1.43E-04
17405414	Serp1	1.75	5.78E-07	2.06E-05
17405667	Ssr3	1.74	2.49E-06	6.24E-05
17439029	Areg	1.74	4.03E-08	2.76E-06
17349582	1700066B19Rik	1.74	3.86E-05	5.38E-04
17339889	Fam82a1	1.74	3.25E-04	2.84E-03
17247389	Egfr	1.74	2.37E-07	1.06E-05
17212947	Spats2l	1.74	1.60E-06	4.52E-05
17261575	Ranbp17	1.74	2.81E-04	2.55E-03
17353420	Fam13b	1.74	1.20E-07	6.30E-06
17316690	Klf10	1.74	1.20E-05	2.15E-04
17447218	Mxd4	1.74	1.93E-04	1.90E-03
17533498	Kdm6a	1.74	5.87E-06	1.22E-04
17243738	Hsp90b1	1.74	2.45E-07	1.08E-05
17439830	Spp1	1.74	1.23E-04	1.33E-03
17413649	Dcaf10	1.74	5.66E-06	1.20E-04
17431152	Dhdds	1.74	3.13E-06	7.47E-05
17308686	Cog3	1.73	5.66E-07	2.03E-05

17434023	Isg15	1.73	4.98E-04	3.95E-03
17292450	Fgd3	1.73	1.08E-04	1.20E-03
17469446	Eif4e3	1.73	2.02E-04	1.98E-03
17491276	Spty2d1	1.73	1.27E-05	2.25E-04
17529449	4922501C03Rik	1.73	1.32E-05	2.32E-04
17250178	Hist3h2a	1.73	6.50E-04	4.88E-03
17252341	Xaf1	1.73	2.98E-05	4.42E-04
17480030	Rab38	1.73	8.31E-04	5.93E-03
17371201	Gca	1.73	2.76E-04	2.52E-03
17370691	Lypd6b	1.72	4.84E-05	6.37E-04
17429632	Mfsd2a	1.72	7.83E-07	2.57E-05
17531238	Arih2	1.72	5.07E-07	1.88E-05
17460451	1600020E01Rik	1.72	3.75E-04	3.17E-03
17235320	Reep6	1.72	1.11E-06	3.35E-05
17260349	Tmed4	1.71	3.73E-06	8.55E-05
17314260	Lrrk2	1.71	1.63E-05	2.77E-04
17525803	Gramd1b	1.71	2.34E-05	3.67E-04
17243644	Cry1	1.71	5.02E-06	1.09E-04
17443015	0610007L01Rik	1.71	2.03E-07	9.40E-06
17231003	Mfsd7b	1.71	1.38E-05	2.40E-04
17307550	Ints6	1.71	5.40E-07	1.98E-05
17281354	Sec23a	1.71	2.28E-07	1.03E-05
17370641	Epc2	1.71	4.36E-07	1.69E-05
17326747	1700066C05Rik	1.71	1.41E-03	9.05E-03
17492289	Det1	1.71	3.15E-05	4.60E-04
17337497	Zfp57	1.71	6.63E-05	8.11E-04
17259794	Rnf185	1.70	6.80E-06	1.36E-04
17508691	Rbpms	1.70	1.94E-06	5.16E-05
17270888	Ern1	1.70	2.41E-07	1.07E-05
17349393	Srp19	1.70	9.08E-07	2.89E-05
17419020	Marcksl1	1.70	9.40E-05	1.08E-03
17337481	Rnf39	1.70	1.97E-05	3.19E-04
17395053	LOC100862165	1.70	1.22E-04	1.33E-03
17496664	Zfp747	1.70	9.59E-05	1.10E-03
17292839	Lman2	1.70	3.19E-07	1.34E-05
17272287	Srp68	1.70	1.71E-07	8.17E-06
17393336	Edem2	1.70	3.00E-07	1.28E-05
17307709	Hmbox1	1.70	1.92E-06	5.16E-05
17441540	Med13l	1.70	9.70E-07	3.03E-05
17421694	Srm	1.70	3.48E-06	8.10E-05
17391888	Rnf24	1.69	3.85E-05	5.38E-04
17223880	Kansl1l	1.69	1.67E-04	1.70E-03
17246091	Il23a	1.69	1.01E-03	6.98E-03
17217610	Csrp1	1.68	3.27E-06	7.71E-05
17214543	Gmppa	1.68	3.48E-05	4.96E-04

17439045	Thap6	1.68	8.53E-05	1.00E-03
17470060	Rassf4	1.68	1.08E-03	7.37E-03
17385654	Itgb6	1.68	1.12E-06	3.36E-05
17291803	1300014I06Rik	1.68	1.85E-06	5.03E-05
17265451	Derl2	1.68	1.15E-06	3.43E-05
17420016	Id3	1.68	2.48E-05	3.81E-04
17443166	Stx1a	1.68	2.99E-05	4.43E-04
17319364	D730005E14Rik	1.68	1.49E-05	2.56E-04
17219546	Slamf9	1.68	2.43E-05	3.76E-04
17289889	Il6st	1.68	9.86E-06	1.82E-04
17287707	Tmed9	1.68	4.36E-06	9.77E-05
17476247	Zfp568	1.68	4.70E-06	1.04E-04
17479875	Stard5	1.68	3.13E-06	7.47E-05
17453347	Gtf2ird1	1.68	1.76E-05	2.93E-04
17283445	Lgmn	1.68	6.78E-05	8.25E-04
17464367	Ppfibp1	1.68	3.43E-06	8.02E-05
17237876	Mettl1	1.67	1.04E-05	1.91E-04
17264102	Hs3st3b1	1.67	6.41E-06	1.31E-04
17355366	Smad4	1.67	5.18E-07	1.91E-05
17388599	Ttc17	1.67	4.32E-06	9.71E-05
17232731	Rnu3a	1.67	3.50E-04	3.00E-03
17331365	Arl13b	1.67	6.91E-06	1.37E-04
17435422	Nub1	1.67	7.41E-06	1.44E-04
17361090	BC021614	1.67	3.85E-06	8.75E-05
17329350	Tbccd1	1.66	2.79E-06	6.85E-05
17439922	Lrrc8d	1.66	1.47E-05	2.54E-04
17254249	Taf15	1.66	3.60E-07	1.46E-05
17442203	Setd1b	1.66	6.78E-07	2.34E-05
17484601	1190003J15Rik	1.66	1.22E-05	2.18E-04
17460918	Tmem43	1.66	1.66E-04	1.69E-03
17450727	Tmed5	1.66	1.37E-07	6.89E-06
17359608	Entpd7	1.66	1.66E-05	2.80E-04
17218209	Edem3	1.66	1.06E-06	3.22E-05
17235890	Hcfc2	1.66	2.35E-05	3.68E-04
17311652	Wdr67	1.66	1.38E-03	8.92E-03
17285859	Hist1h1c	1.66	4.81E-05	6.34E-04
17279230	Zfyve21	1.66	1.26E-06	3.72E-05
17295482	Mtap1b	1.66	5.95E-06	1.24E-04
17412552	Ube2j1	1.66	1.28E-07	6.60E-06
17290949	Zfp187	1.66	5.70E-04	4.40E-03
17329298	Etv5	1.65	7.84E-05	9.34E-04
17525379	Fam118b	1.65	1.32E-04	1.41E-03
17331044	Tfg	1.65	1.26E-07	6.50E-06
17280577	Hbp1	1.65	4.40E-06	9.81E-05
17366597	Usp6nl	1.65	5.22E-07	1.92E-05

17328978	Cdc45	1.65	7.62E-06	1.47E-04
17320583	Cpne8	1.65	5.19E-05	6.68E-04
17413369	Creb3	1.65	3.30E-06	7.77E-05
17487457	Bcl3	1.65	3.17E-07	1.33E-05
17264424	Ndel1	1.65	8.20E-06	1.56E-04
17342065	Mapk8ip3	1.65	5.47E-05	7.00E-04
17291937	Txndc5	1.65	5.77E-07	2.06E-05
17307425	LOC100862065	1.65	1.18E-03	7.91E-03
17294167	BC018507	1.64	6.64E-05	8.11E-04
17483842	Tacc2	1.64	9.13E-07	2.89E-05
17346679	Pja2	1.64	1.75E-06	4.82E-05
17313050	Apobec3	1.64	7.21E-06	1.40E-04
17445394	A330021E22Rik	1.64	6.94E-05	8.41E-04
17430487	Txlna	1.64	2.24E-05	3.53E-04
17511130	Ier2	1.64	3.70E-05	5.20E-04
17216087	Ing5	1.64	3.10E-06	7.43E-05
17510885	Elmod2	1.64	8.45E-07	2.72E-05
17518500	Parp16	1.64	4.99E-05	6.50E-04
17437302	Slit2	1.63	1.83E-04	1.83E-03
17371661	Gorasp2	1.63	5.80E-07	2.06E-05
17452405	Atp2a2	1.63	3.04E-07	1.29E-05
17419553	Map3k6	1.63	3.69E-05	5.18E-04
17274868	2010109K11Rik	1.63	6.82E-06	1.36E-04
17488470	Samd4b	1.63	1.85E-05	3.05E-04
17363470	Gda	1.63	4.63E-07	1.75E-05
17543396	Eda2r	1.63	3.02E-04	2.69E-03
17335540	Pim1	1.62	1.34E-04	1.43E-03
17330870	Gm4827	1.62	1.16E-05	2.09E-04
17388999	Prrg4	1.62	4.36E-06	9.77E-05
17246679	Slc35e4	1.62	3.68E-06	8.47E-05
17392333	Rrbp1	1.62	7.21E-07	2.44E-05
17443350	Ywhag	1.62	4.57E-06	1.01E-04
17451748	Ccdc64	1.62	3.52E-05	5.00E-04
17233736	Sar1a	1.62	4.52E-07	1.73E-05
17301849	Fndc3a	1.62	1.40E-06	4.05E-05
17297595	Myst4	1.61	2.45E-04	2.29E-03
17361833	Dpf2	1.61	9.82E-07	3.06E-05
17393501	Rbm39	1.61	7.84E-07	2.57E-05
17534909	Ddx26b	1.61	4.99E-05	6.50E-04
17438062	Nipal1	1.61	4.75E-07	1.79E-05
17479183	Fam174b	1.61	1.40E-06	4.05E-05
17276153	Daam1	1.61	1.93E-06	5.16E-05
17368456	Cacfd1	1.61	2.18E-06	5.66E-05
17419041	LOC100861795	1.61	3.27E-05	4.74E-04
17231801	Reps1	1.61	1.00E-06	3.11E-05

17480349	Kctd14	1.60	1.28E-04	1.38E-03
17376510	Smox	1.60	4.47E-05	6.02E-04
17492314	Mfge8	1.60	1.94E-05	3.16E-04
17317167	Has2	1.60	1.07E-03	7.32E-03
17409075	Csf1	1.60	3.91E-04	3.26E-03
17301558	Kctd9	1.60	1.05E-05	1.92E-04
17544939	Tsc22d3	1.60	3.74E-04	3.17E-03
17425160	Erp44	1.60	5.88E-07	2.08E-05
17473410	Zfp628	1.60	3.46E-04	2.98E-03
17531176	Tcta	1.60	2.37E-05	3.70E-04
17355298	2310002L13Rik	1.59	4.80E-06	1.06E-04
17342943	4930539E08Rik	1.59	2.75E-05	4.15E-04
17330080	Dirc2	1.59	3.02E-06	7.30E-05
17226771	Ikbke	1.59	8.96E-05	1.04E-03
17445525	Rundc3b	1.59	8.81E-05	1.03E-03
17472598	5730419I09Rik	1.59	1.97E-05	3.19E-04
17325410	Golgb1	1.59	7.45E-05	8.93E-04
17401514	4933421E11Rik	1.59	6.65E-06	1.34E-04
17479834	Mex3b	1.59	8.61E-05	1.01E-03
17355009	Nars	1.59	7.65E-07	2.53E-05
17322128	Spryd3	1.59	8.60E-06	1.62E-04
17222106	Uggt1	1.59	6.84E-06	1.36E-04
17543529	Slc7a3	1.59	6.13E-04	4.66E-03
17317015	Ext1	1.59	4.47E-07	1.71E-05
17431820	Pla2g5	1.59	2.22E-04	2.13E-03
17276328	Hif1a	1.59	6.02E-07	2.13E-05
17448863	Scfd2	1.58	1.12E-03	7.54E-03
17405591	Slc33a1	1.58	1.98E-05	3.20E-04
17359925	Gbf1	1.58	6.59E-06	1.34E-04
17497044	Ikzf5	1.58	1.24E-04	1.34E-03
17228349	Ier5	1.58	1.26E-05	2.23E-04
17406962	Trim46	1.58	9.75E-04	6.77E-03
17233457	Ranbp2	1.58	1.03E-05	1.89E-04
17244913	Zdhhc17	1.58	4.18E-05	5.73E-04
17371059	Mar-07	1.58	5.10E-05	6.58E-04
17449596	Rchy1	1.57	4.78E-06	1.05E-04
17342708	Lemd2	1.57	1.42E-04	1.49E-03
17472847	4933424B01Rik	1.57	6.80E-05	8.27E-04
17432426	Prdm2	1.57	6.67E-04	4.97E-03
17399432	Shc1	1.57	1.78E-06	4.85E-05
17532472	Cdcp1	1.57	7.69E-06	1.48E-04
17243525	Slc41a2	1.57	5.61E-05	7.14E-04
17343119	Btbd9	1.57	1.39E-04	1.47E-03
17485826	Ptprh	1.57	2.98E-05	4.42E-04
17431496	Pnrc2	1.57	1.52E-06	4.33E-05

17294974	Papd4	1.57	3.93E-06	8.94E-05
17505851	Nudt7	1.57	2.19E-05	3.47E-04
17251799	Gps2	1.56	1.87E-06	5.04E-05
17294991	Jmy	1.56	3.18E-04	2.79E-03
17474956	1500002O20Rik	1.56	5.85E-06	1.22E-04
17532137	Myd88	1.56	7.60E-05	9.09E-04
17399104	Ssr2	1.56	5.08E-07	1.88E-05
17260167	Ccdc117	1.56	1.07E-05	1.95E-04
17416336	Ttc22	1.56	2.74E-05	4.15E-04
17354857	Adrb2	1.56	1.35E-04	1.43E-03
17447289	Nop14	1.56	4.37E-07	1.69E-05
17342101	Hn1l	1.56	2.50E-06	6.25E-05
17409376	Scarna2	1.56	1.16E-03	7.80E-03
17407116	Cks1b	1.56	2.94E-04	2.64E-03
17334192	Prss27	1.56	4.10E-04	3.38E-03
17452713	Vps37b	1.55	3.04E-04	2.70E-03
17249593	Irf1	1.55	9.36E-06	1.75E-04
17409379	Tmem167b	1.55	1.70E-05	2.85E-04
17222001	Prim2	1.55	1.43E-05	2.49E-04
17295770	Slc30a5	1.55	1.38E-04	1.46E-03
17420546	Pla2g2e	1.55	4.15E-04	3.42E-03
17490924	Sphk2	1.55	8.56E-06	1.61E-04
17225472	Zfp330	1.55	3.51E-07	1.44E-05
17402746	Ints12	1.55	2.86E-05	4.28E-04
17521825	Qrich1	1.55	1.60E-06	4.52E-05
17359967	Nfkb2	1.55	1.14E-05	2.07E-04
17343768	Brd2	1.55	1.50E-06	4.28E-05
17258887	Pgs1	1.55	7.33E-05	8.81E-04
17304246	Appl1	1.55	1.86E-06	5.03E-05
17339549	Ypel5	1.55	1.10E-06	3.35E-05
17422907	2310042D19Rik	1.55	2.52E-04	2.34E-03
17543974	Magt1	1.55	4.65E-06	1.03E-04
17241582	Sirt1	1.54	6.74E-04	5.01E-03
17469541	Il5ra	1.54	1.99E-05	3.21E-04
17271259	Wipi1	1.54	3.77E-05	5.27E-04
17462998	Zfp384	1.54	1.31E-05	2.30E-04
17437129	Cpeb2	1.54	4.19E-05	5.73E-04
17502874	Tbc1d9	1.54	6.18E-06	1.28E-04
17461725	Creld1	1.54	2.58E-04	2.39E-03
17487500	2210010C17Rik	1.54	3.72E-05	5.22E-04
17224617	LOC100862164	1.54	2.85E-06	6.97E-05
17449578	Gm19619	1.54	1.12E-03	7.56E-03
17484627	Ric8	1.54	1.59E-06	4.49E-05
17464503	2810474O19Rik	1.54	1.14E-05	2.06E-04
17537756	Armcx3	1.54	2.09E-05	3.34E-04

17339142	Ppp4r1	1.53	1.92E-06	5.15E-05
17332228	Rcan1	1.53	4.96E-05	6.47E-04
17453832	Trim56	1.53	7.83E-06	1.50E-04
17364932	Got1	1.53	3.45E-06	8.04E-05
17358962	Btaf1	1.53	6.16E-05	7.67E-04
17357150	Wdr74	1.53	6.44E-05	7.94E-04
17407578	Tuft1	1.53	6.53E-05	8.02E-04
17497091	Oat	1.53	1.15E-05	2.08E-04
17443901	Fam20c	1.53	1.52E-03	9.68E-03
17390433	Zscan29	1.53	1.83E-05	3.02E-04
17327465	Ets2	1.53	5.49E-06	1.17E-04
17258220	Slc16a5	1.53	3.02E-04	2.69E-03
17344021	Stk19	1.53	3.48E-04	2.99E-03
17343387	Akap8l	1.53	6.45E-05	7.94E-04
17289794	Plk2	1.53	8.23E-06	1.57E-04
17238162	Rdh9	1.53	2.51E-04	2.33E-03
17273165	P4hb	1.53	1.00E-06	3.11E-05
17525524	Stt3a	1.53	7.28E-07	2.45E-05
17459634	Ggcx	1.52	1.03E-04	1.16E-03
17444364	2810453I06Rik	1.52	1.34E-03	8.72E-03
17266475	Tnfaip1	1.52	2.80E-06	6.85E-05
17479706	Whamm	1.52	1.44E-05	2.49E-04
17325651	Igsf11	1.52	2.56E-05	3.91E-04
17281280	Mbip	1.52	6.55E-05	8.03E-04
17483385	Phkg2	1.52	7.33E-06	1.43E-04
17327524	Igsf5	1.52	5.54E-06	1.18E-04
17271118	Pitpnc1	1.52	8.97E-06	1.68E-04
17330099	Parp14	1.52	2.88E-06	7.02E-05
17481982	Far1	1.52	8.40E-06	1.59E-04
17257660	Gm11714	1.52	1.13E-04	1.25E-03
17427345	Cyp2j12	1.52	8.10E-05	9.59E-04
17418916	Rnf19b	1.52	6.03E-05	7.56E-04
17527977	Glce	1.52	5.29E-05	6.80E-04
17479616	Zfp592	1.52	9.65E-06	1.80E-04
17469289	Eogt	1.52	4.31E-04	3.53E-03
17401846	Vav3	1.52	1.14E-04	1.26E-03
17216201	Gin1	1.51	3.51E-04	3.00E-03
17302472	Kctd12	1.51	3.67E-04	3.12E-03
17347042	Ndc80	1.51	2.18E-04	2.09E-03
17388406	Slc35c1	1.51	1.46E-06	4.19E-05
17355231	Ptpn2	1.51	1.71E-05	2.87E-04
17348840	Rnf125	1.51	4.24E-04	3.48E-03
17231387	Mthfd1l	1.51	1.02E-04	1.15E-03
17468018	Hk2	1.51	5.68E-06	1.20E-04
17233149	Ascc3	1.51	3.06E-05	4.52E-04

17350952	Rbm22	1.51	1.03E-06	3.19E-05
17285879	Hist1h1a	1.51	2.38E-04	2.24E-03
17310673	Ank	1.51	4.53E-05	6.08E-04
17541760	Mospd1	1.51	2.75E-06	6.78E-05
17430521	Tmem39b	1.51	1.87E-05	3.06E-04
17344114	1110038B12Rik	1.51	4.23E-06	9.53E-05
17398785	Arhgef11	1.51	3.62E-05	5.10E-04
17446881	Eif2b4	1.51	3.69E-04	3.13E-03
17484295	Ppp2r2d	1.50	3.68E-06	8.47E-05
17338258	AI661453	1.50	3.88E-05	5.40E-04
17450059	Sec31a	1.50	2.27E-06	5.82E-05
17258178	Cdr2l	1.50	8.87E-04	6.27E-03
17299750	Tox4	1.50	2.39E-04	2.25E-03
17214421	Zfand2b	1.50	1.01E-04	1.14E-03
17447446	Tada2b	1.50	2.71E-04	2.49E-03
17332236	Runx1	1.50	2.71E-04	2.48E-03
17255558	Hoxb9	1.50	3.00E-05	4.44E-04
17440409	Golga3	1.50	1.73E-04	1.75E-03
17224587	Dnpep	1.50	2.20E-06	5.71E-05
17265625	Med31	1.50	1.83E-04	1.83E-03
17529383	Pgm3	1.50	2.51E-05	3.84E-04
17488167	Itpkc	1.50	1.14E-03	7.69E-03
17513835	Ankrd11	1.50	6.26E-06	1.29E-04
17345465	Srf	1.50	4.10E-05	5.64E-04
17368834	Med27	1.50	2.64E-04	2.43E-03
17424674	Tln1	1.50	6.59E-05	8.06E-04
17364251	Ide	1.50	5.98E-06	1.24E-04
17371436	Bbs5	1.50	3.68E-04	3.13E-03
17429234	Tmem125	1.50	1.07E-04	1.19E-03
17340584	Tmem181c-ps	1.50	9.40E-04	6.57E-03
17361971	Sac3d1	1.50	3.40E-05	4.90E-04
17380892	Arfgap1	1.50	7.64E-06	1.47E-04
17490909	A030001D20Rik	1.50	4.88E-04	3.89E-03
17270999	Ddx5	1.49	6.06E-04	4.61E-03
17419608	Fam46b	1.49	1.60E-04	1.65E-03
17230595	Degs1	1.49	6.79E-06	1.36E-04
17307117	Gjb2	1.49	4.38E-05	5.94E-04
17352203	Zfp516	1.49	2.30E-04	2.18E-03
17405662	A730090N16Rik	1.49	1.13E-03	7.60E-03
17245831	Mbd6	1.49	6.97E-05	8.43E-04
17421033	Epha2	1.49	4.78E-05	6.32E-04
17521371	Tmem115	1.49	4.27E-04	3.50E-03
17437054	Stx18	1.49	4.10E-05	5.64E-04
17222825	Obfc2a	1.49	3.82E-06	8.70E-05
17300565	Rnf31	1.49	1.21E-03	8.04E-03

17361656	Ovol1	1.49	5.42E-04	4.23E-03
17316831	Nudcd1	1.49	1.04E-04	1.18E-03
17407684	Pip5k1a	1.49	1.01E-04	1.15E-03
17475538	LOC100862260	1.49	2.18E-04	2.10E-03
17462351	Il17ra	1.49	2.38E-05	3.71E-04
17326208	Cblb	1.48	2.76E-05	4.16E-04
17311179	Fzd6	1.48	4.77E-05	6.30E-04
17495692	Dcun1d3	1.48	1.47E-04	1.54E-03
17281843	Dhrs7	1.48	4.17E-04	3.43E-03
17518378	Dennd4a	1.48	5.20E-04	4.10E-03
17485528	Tfpt	1.48	2.80E-04	2.54E-03
17519967	Tpbg	1.48	4.88E-05	6.39E-04
17326166	Cd47	1.48	2.58E-06	6.41E-05
17291904	Ssr1	1.48	6.37E-05	7.88E-04
17348648	Taf4b	1.48	1.68E-04	1.71E-03
17390561	Catsper2	1.48	4.84E-05	6.37E-04
17501976	Isyna1	1.48	4.40E-05	5.95E-04
17239702	Tbpl1	1.48	5.93E-06	1.24E-04
17380850	Slc17a9	1.48	2.42E-05	3.76E-04
17485937	Ube2s	1.48	3.58E-05	5.07E-04
17379229	Tox2	1.48	1.56E-03	9.90E-03
17346317	Lonp1	1.48	8.50E-06	1.61E-04
17295697	Rad17	1.47	9.28E-04	6.51E-03
17538161	Prps1	1.47	3.15E-04	2.77E-03
17210912	Rb1cc1	1.47	2.83E-05	4.24E-04
17316175	Basp1	1.47	4.20E-05	5.74E-04
17274496	Taf1b	1.47	1.15E-03	7.73E-03
17532953	Tfe3	1.47	1.55E-04	1.60E-03
17356401	Eif1ad	1.47	1.74E-04	1.75E-03
17509435	BC088983	1.47	5.59E-04	4.34E-03
17488975	Tbcb	1.47	1.08E-05	1.97E-04
17253688	Ift20	1.47	1.44E-05	2.49E-04
17454877	Usp42	1.47	5.48E-04	4.27E-03
17329776	Senp5	1.47	7.17E-04	5.28E-03
17268180	Myst2	1.47	1.35E-04	1.43E-03
17408211	Pde4dip	1.47	1.32E-04	1.41E-03
17503937	Mt2	1.47	1.69E-05	2.84E-04
17272555	Tmc6	1.47	6.50E-05	7.99E-04
17548094	Gnpnat1	1.47	1.27E-04	1.37E-03
17329195	Thpo	1.47	8.22E-04	5.88E-03
17470580	Apobec1	1.47	1.66E-05	2.80E-04
17439909	Lrrc8c	1.47	5.64E-04	4.37E-03
17225728	Hdlbp	1.47	5.61E-06	1.19E-04
17429026	Kdm4a	1.47	4.30E-05	5.85E-04
17306835	Rabggta	1.47	4.13E-04	3.40E-03

17371427	Dhrs9	1.47	1.22E-05	2.18E-04
17327069	Ifnar1	1.46	1.32E-04	1.41E-03
17407240	Rps27	1.46	1.40E-03	9.03E-03
17245248	Mdm2	1.46	6.54E-05	8.02E-04
17213295	Nop58	1.46	5.79E-05	7.31E-04
17485706	Cdc42ep5	1.46	4.72E-04	3.79E-03
17459918	Pcgf1	1.46	1.02E-04	1.16E-03
17255918	Cdk12	1.46	5.60E-05	7.13E-04
17413817	Foxe1	1.46	5.12E-04	4.04E-03
17454704	Tnrc18	1.46	2.17E-05	3.45E-04
17232672	Traf3ip2	1.46	1.93E-04	1.91E-03
17261289	Eml6	1.46	7.34E-04	5.36E-03
17399044	Smg5	1.46	3.98E-05	5.52E-04
17275405	Heatr5a	1.46	1.38E-04	1.46E-03
17452178	Trafd1	1.46	7.01E-05	8.46E-04
17453496	Cldn4	1.46	2.23E-06	5.75E-05
17531265	Atrip	1.46	3.48E-04	2.99E-03
17398082	Tiparp	1.46	6.21E-04	4.71E-03
17433405	Phf13	1.46	8.18E-05	9.67E-04
17356848	Sf1	1.46	6.51E-06	1.32E-04
17311643	Zhx2	1.46	7.21E-04	5.30E-03
17402305	Bcar3	1.46	1.77E-04	1.78E-03
17379911	Rnf114	1.46	6.58E-04	4.92E-03
17273511	Wdr45l	1.46	6.52E-04	4.89E-03
17221347	Tram1	1.46	1.97E-06	5.22E-05
17314487	Ano6	1.46	1.72E-05	2.88E-04
17456584	Calu	1.46	2.20E-06	5.70E-05
17258584	Sphk1	1.45	1.27E-03	8.37E-03
17357971	Gna14	1.45	9.72E-04	6.76E-03
17443208	Tbl2	1.45	3.47E-05	4.96E-04
17375275	Pdia3	1.45	2.45E-05	3.79E-04
17218060	Ptgs2	1.45	5.08E-05	6.57E-04
17221960	Ptp4a1	1.45	1.08E-04	1.21E-03
17438113	Cwh43	1.45	9.78E-06	1.82E-04
17323120	Nde1	1.45	1.34E-03	8.72E-03
17305167	Ghitm	1.45	4.34E-06	9.75E-05
17346185	Dpp9	1.45	2.66E-05	4.03E-04
17327681	Crebbp	1.45	2.14E-05	3.40E-04
17359878	Pprc1	1.45	7.93E-05	9.42E-04
17496253	Atxn2l	1.45	4.36E-05	5.92E-04
17235584	Dapk3	1.45	3.79E-04	3.19E-03
17488544	Nfkbib	1.45	3.24E-05	4.70E-04
17546234	Vamp7	1.45	1.24E-04	1.34E-03
17420561	Tmco4	1.45	1.31E-04	1.41E-03
17270761	Ccdc47	1.45	2.93E-05	4.37E-04

17221186	Snhg6	1.45	6.87E-06	1.36E-04
17395955	Hnf4g	1.44	8.81E-06	1.65E-04
17347953	Fbxo11	1.44	6.87E-06	1.36E-04
17217580	Arl8a	1.44	1.72E-04	1.74E-03
17337557	H2-M3	1.44	9.85E-04	6.83E-03
17213192	Cflar	1.44	2.07E-04	2.02E-03
17405365	Wwtr1	1.44	2.43E-05	3.76E-04
17357502	Cpsf7	1.44	3.44E-05	4.93E-04
17453703	Prkrip1	1.44	1.40E-04	1.48E-03
17454836	E130309D02Rik	1.44	6.51E-06	1.32E-04
17439481	Bmp2k	1.44	2.95E-05	4.39E-04
17290629	Lgals8	1.44	1.87E-05	3.07E-04
17399164	Rit1	1.44	9.66E-05	1.11E-03
17500270	Zfp703	1.44	9.43E-05	1.08E-03
17527505	1700017B05Rik	1.44	1.33E-04	1.42E-03
17285000	Klf6	1.44	2.57E-05	3.92E-04
17314222	Acr	1.44	5.78E-04	4.45E-03
17290770	Mrpl32	1.44	1.09E-03	7.42E-03
17445325	Fzd1	1.44	1.53E-05	2.62E-04
17492729	Sec11a	1.44	4.27E-05	5.82E-04
17223616	Raph1	1.44	2.42E-05	3.75E-04
17311945	Phf20l1	1.44	1.41E-04	1.49E-03
17324446	Rtp4	1.43	7.55E-05	9.04E-04
17227266	Elf3	1.43	2.62E-05	3.98E-04
17270583	Nsf	1.43	3.17E-06	7.55E-05
17432264	Plekhm2	1.43	2.94E-04	2.63E-03
17460572	Copg	1.43	1.31E-05	2.31E-04
17351282	Nedd4l	1.43	3.02E-04	2.69E-03
17343278	Hsf2bp	1.43	1.49E-03	9.53E-03
17318249	Fam83h	1.43	3.57E-05	5.07E-04
17349474	Kdm3b	1.43	3.45E-05	4.94E-04
17523161	Slc25a38	1.43	1.44E-04	1.52E-03
17279805	Ncoa1	1.43	3.57E-04	3.05E-03
17288732	Ttc37	1.43	6.95E-05	8.41E-04
17352146	Pqlc1	1.43	4.86E-05	6.38E-04
17438196	Fip1l1	1.43	1.83E-05	3.03E-04
17360348	Dusp5	1.43	1.16E-04	1.28E-03
17361779	Scyl1	1.42	2.48E-05	3.81E-04
17434027	Plekhn1	1.42	8.55E-04	6.07E-03
17335441	Kctd20	1.42	7.23E-04	5.30E-03
17235211	Midn	1.42	5.02E-05	6.54E-04
17344706	Trim39	1.42	9.70E-04	6.74E-03
17393357	Eif6	1.42	5.06E-05	6.55E-04
17298426	Capn7	1.42	2.09E-05	3.34E-04
17313811	Fbln1	1.42	3.91E-04	3.26E-03

17515786	Arhgap32	1.42	5.58E-04	4.34E-03
17396702	Zfp639	1.42	6.07E-05	7.60E-04
17252038	Zmynd15	1.42	1.13E-04	1.25E-03
17492523	Unc45a	1.42	2.69E-04	2.47E-03
17358051	Trpm6	1.42	3.85E-04	3.23E-03
17496806	Prss8	1.42	1.33E-04	1.42E-03
17327995	Carhsp1	1.42	3.22E-05	4.68E-04
17265710	Cyb5d2	1.42	2.42E-04	2.27E-03
17445568	Dmtf1	1.42	8.36E-06	1.59E-04
17524731	Yipf2	1.42	1.31E-04	1.40E-03
17358422	Vldlr	1.42	2.72E-04	2.49E-03
17431065	Pigv	1.42	8.46E-04	6.02E-03
17344497	Mrps18b	1.42	2.89E-04	2.60E-03
17480102	Sytl2	1.41	3.39E-04	2.93E-03
17434381	Krit1	1.41	1.19E-03	7.92E-03
17488406	Supt5h	1.41	1.62E-04	1.66E-03
17236688	Tmcc3	1.41	3.02E-05	4.46E-04
17447170	Whsc2	1.41	5.04E-05	6.55E-04
17538912	Kdm5c	1.41	8.44E-04	6.01E-03
17326438	Cldn25	1.41	4.53E-05	6.08E-04
17367071	Stam	1.41	1.55E-05	2.66E-04
17484469	Zfp511	1.41	5.68E-04	4.39E-03
17515819	Ets1	1.41	3.23E-04	2.82E-03
17219264	B4galt3	1.41	8.28E-04	5.92E-03
17422269	A430005L14Rik	1.41	2.11E-04	2.05E-03
17346175	D17Wsu104e	1.41	2.25E-05	3.54E-04
17400813	Notch2	1.41	3.23E-04	2.82E-03
17441759	Oas1b	1.41	3.92E-04	3.27E-03
17265268	Cxcl16	1.41	1.22E-05	2.17E-04
17305748	Gch1	1.41	4.67E-04	3.77E-03
17382013	Dnajc1	1.41	5.06E-05	6.55E-04
17513176	Tmem170	1.41	7.19E-05	8.67E-04
17274532	Klf11	1.41	5.26E-04	4.13E-03
17536292	Zfx	1.41	1.18E-03	7.90E-03
17279381	AW555464	1.41	1.24E-05	2.20E-04
17350936	Dctn4	1.41	1.16E-05	2.09E-04
17476297	Polr2i	1.41	3.26E-05	4.73E-04
17378721	Src	1.41	3.26E-05	4.72E-04
17361741	Ehbp1l1	1.41	5.18E-05	6.67E-04
17343724	Ring1	1.41	4.70E-04	3.78E-03
17219448	Copa	1.41	1.56E-05	2.68E-04
17424042	Aptx	1.40	6.45E-04	4.86E-03
17337228	Ier3	1.40	2.64E-05	4.00E-04
17409594	Rnpc3	1.40	1.18E-03	7.88E-03
17443972	Get4	1.40	2.66E-04	2.44E-03

17384252	Gapvd1	1.40	4.02E-05	5.56E-04
17343263	Sik1	1.40	9.51E-04	6.63E-03
17466003	Zc3hav1	1.40	1.35E-04	1.43E-03
17270919	Tex2	1.40	2.73E-04	2.50E-03
17229502	Uap1	1.40	8.02E-06	1.53E-04
17469035	LOC100861999	1.40	2.60E-05	3.96E-04
17368526	Wdr5	1.40	1.27E-03	8.33E-03
17456131	Tes	1.40	1.68E-05	2.83E-04
17400467	Otud7b	1.40	1.40E-04	1.47E-03
17287338	Secisbp2	1.40	3.16E-04	2.77E-03
17321761	Smagp	1.40	1.28E-05	2.26E-04
17282868	Sptlc2	1.40	2.96E-05	4.40E-04
17420646	Ubr4	1.40	3.86E-04	3.23E-03
17281582	Sos2	1.40	1.17E-04	1.29E-03
17442977	Crcp	1.40	1.07E-04	1.19E-03
17369860	Dpm2	1.40	1.54E-03	9.79E-03
17483532	Kat8	1.40	1.81E-04	1.81E-03
17437598	0610040J01Rik	1.40	7.42E-04	5.41E-03
17274540	Rrm2	1.40	5.89E-04	4.51E-03
17249282	Clk4	1.39	1.22E-04	1.33E-03
17485702	Leng9	1.39	6.53E-04	4.90E-03
17413932	Sec61b	1.39	1.40E-03	9.03E-03
17224462	1810031K17Rik	1.39	1.75E-04	1.76E-03
17445257	C030048B08Rik	1.39	1.17E-03	7.84E-03
17314011	Pim3	1.39	4.09E-04	3.38E-03
17507082	Pex11c	1.39	9.20E-04	6.46E-03
17358891	Pcgf5	1.39	3.81E-04	3.20E-03
17526206	C2cd2l	1.39	3.09E-05	4.54E-04
17487796	Rabac1	1.39	1.88E-04	1.87E-03
17482590	Polr3e	1.39	3.32E-04	2.88E-03
17210869	Tcea1	1.39	2.21E-05	3.49E-04
17465788	Cnot4	1.39	5.80E-05	7.32E-04
17468298	Exoc6b	1.39	3.22E-05	4.68E-04
17273751	Itsn2	1.39	2.58E-04	2.38E-03
17449562	Rassf6	1.39	5.70E-04	4.40E-03
17263708	Usp22	1.39	5.71E-05	7.23E-04
17503559	Tmem188	1.39	1.21E-03	8.04E-03
17355213	Cep76	1.39	6.98E-04	5.16E-03
17499540	Mcph1	1.39	1.08E-04	1.21E-03
17220166	Acbd3	1.39	1.89E-05	3.10E-04
17494041	Nup98	1.39	2.93E-04	2.63E-03
17409406	Stxbp3a	1.39	1.14E-04	1.26E-03
17356447	Efemp2	1.39	7.19E-04	5.28E-03
17393036	Plagl2	1.39	8.58E-05	1.01E-03
17491440	Nipa2	1.39	7.14E-04	5.26E-03

17495475	Smg1	1.38	1.34E-03	8.74E-03
17498041	Dusp8	1.38	2.22E-04	2.13E-03
17216373	Phlpp1	1.38	1.93E-04	1.91E-03
17258664	Mfsd11	1.38	9.43E-04	6.59E-03
17281946	Ppp2r5e	1.38	4.64E-05	6.19E-04
17490960	Cyth2	1.38	9.39E-05	1.08E-03
17259600	Foxk2	1.38	8.30E-04	5.93E-03
17386770	Fkbp7	1.38	1.36E-03	8.81E-03
17256806	Rdm1	1.38	1.58E-03	9.98E-03
17495271	Copb1	1.38	3.29E-05	4.76E-04
17397774	Tsc22d2	1.38	4.63E-04	3.75E-03
17228563	Tor3a	1.38	2.90E-04	2.61E-03
17365030	Chuk	1.38	2.05E-04	2.00E-03
17456318	Ing3	1.38	4.91E-04	3.90E-03
17473786	Zscan22	1.38	6.87E-04	5.09E-03
17349311	Wdr36	1.37	1.13E-03	7.59E-03
17349745	Ik	1.37	1.63E-05	2.77E-04
17320163	Brd1	1.37	9.70E-05	1.11E-03
17451769	Prkab1	1.37	1.41E-04	1.48E-03
17532923	Wdr45	1.37	5.04E-04	3.99E-03
17290797	Cdk13	1.37	3.77E-04	3.18E-03
17365692	Smndc1	1.37	2.16E-04	2.08E-03
17485747	Ppp1r12c	1.37	3.71E-04	3.14E-03
17518142	Tle3	1.37	1.40E-03	9.00E-03
17494005	Rnf121	1.37	1.89E-04	1.88E-03
17216870	Ubxn4	1.37	4.49E-05	6.04E-04
17506949	Pard3	1.37	1.82E-04	1.82E-03
17222837	Myo1b	1.37	5.29E-04	4.15E-03
17495005	Tmem41b	1.37	3.05E-04	2.71E-03
17404947	Ankrd50	1.37	7.65E-04	5.55E-03
17273086	Nploc4	1.37	2.80E-05	4.21E-04
17435771	Ept1	1.37	3.59E-05	5.07E-04
17521593	Ip6k1	1.37	1.04E-03	7.12E-03
17369218	Dolpp1	1.37	9.29E-05	1.08E-03
17398020	D630022N01Rik	1.37	9.57E-04	6.67E-03
17239090	Tab2	1.37	1.79E-04	1.80E-03
17297305	Ngly1	1.37	3.43E-05	4.93E-04
17372705	Tnks1bp1	1.37	3.68E-05	5.17E-04
17342642	Dusp1	1.37	1.00E-04	1.14E-03
17237142	Ppp1r12a	1.37	3.12E-04	2.75E-03
17493614	Uvrag	1.37	3.39E-04	2.94E-03
17491979	Mef2a	1.37	1.78E-04	1.79E-03
17407969	Plekho1	1.37	6.36E-04	4.80E-03
17216638	Tmem185b	1.37	5.27E-04	4.13E-03
17497769	Rnh1	1.37	5.85E-04	4.49E-03

17506081	Osgin1	1.36	8.50E-04	6.05E-03
17212373	Tpp2	1.36	7.86E-05	9.36E-04
17483772	Sec23ip	1.36	6.09E-05	7.61E-04
17336838	Vars	1.36	1.33E-03	8.68E-03
17385030	Mmadhc	1.36	1.36E-04	1.44E-03
17436237	Fosl2	1.36	4.25E-05	5.80E-04
17420486	Ddost	1.36	3.95E-04	3.29E-03
17430989	Tmem222	1.36	1.86E-04	1.85E-03
17233294	Nus1	1.36	5.66E-05	7.17E-04
17382104	Arhgap21	1.36	6.41E-05	7.91E-04
17361285	Syt12	1.36	2.02E-04	1.98E-03
17392025	Trmt6	1.36	2.48E-04	2.31E-03
17546834	Ddx3y	1.36	7.37E-04	5.38E-03
17355955	1810055G02Rik	1.36	2.04E-04	1.99E-03
17348410	Rbbp8	1.36	8.19E-04	5.87E-03
17372392	Dnajc10	1.36	9.83E-05	1.12E-03
17345293	Vegfa	1.36	1.71E-04	1.73E-03
17507308	Slc10a2	1.36	1.25E-04	1.35E-03
17460059	Mob1a	1.36	4.89E-04	3.90E-03
17348449	Riok3	1.36	4.80E-05	6.34E-04
17503333	Tnpo2	1.36	4.00E-05	5.54E-04
17256734	Ifi35	1.36	1.58E-03	9.98E-03
17222913	Nab1	1.35	5.69E-04	4.40E-03
17407905	Prpf3	1.35	3.29E-04	2.87E-03
17516558	H2afx	1.35	3.20E-04	2.81E-03
17362320	AI846148	1.35	1.03E-03	7.05E-03
17465373	Impdh1	1.35	3.80E-04	3.20E-03
17548715	LOC100861831	1.35	1.20E-03	7.98E-03
17233534	Sowahc	1.35	2.84E-04	2.57E-03
17220364	Srp9	1.35	2.36E-04	2.22E-03
17437674	Klf3	1.35	1.63E-04	1.67E-03
17349671	Ankhd1	1.35	7.11E-04	5.24E-03
17231859	Ifngr1	1.35	3.36E-05	4.84E-04
17348983	2700062C07Rik	1.35	1.58E-03	9.97E-03
17357486	Syt7	1.35	1.00E-03	6.90E-03
17336322	Rxrb	1.35	3.16E-04	2.77E-03
17243252	Tjp3	1.35	1.99E-05	3.21E-04
17288699	Ell2	1.35	1.24E-04	1.34E-03
17458020	Arhgef5	1.35	1.67E-04	1.70E-03
17501891	Cope	1.35	1.04E-04	1.18E-03
17245709	Os9	1.34	4.22E-04	3.46E-03
17231545	Lats1	1.34	2.14E-04	2.07E-03
17499376	Upf3a	1.34	3.44E-04	2.97E-03
17428192	Rnf11	1.34	5.20E-05	6.69E-04
17297913	Arf4	1.34	2.57E-05	3.92E-04

17248592	Slu7	1.34	1.07E-04	1.20E-03
17506846	Gm505	1.34	1.92E-04	1.90E-03
17426126	Fkbp15	1.34	8.12E-04	5.83E-03
17245013	Tbc1d15	1.34	9.01E-05	1.05E-03
17431530	Tceb3	1.34	4.44E-04	3.62E-03
17252817	Mnt	1.34	4.71E-04	3.79E-03
17348295	Thoc1	1.34	7.82E-04	5.65E-03
17521485	Mst1r	1.34	1.45E-04	1.52E-03
17341671	Prss22	1.34	6.94E-04	5.13E-03
17269717	Stat3	1.34	3.58E-05	5.07E-04
17378300	Itch	1.34	8.06E-04	5.80E-03
17360877	Fam45a	1.34	3.42E-04	2.96E-03
17444236	Wipi2	1.34	1.58E-04	1.63E-03
17436574	Rnf4	1.34	2.58E-04	2.39E-03
17505995	Plcg2	1.34	3.80E-04	3.20E-03
17265111	Rai12	1.34	6.55E-05	8.03E-04
17438438	Srp72	1.34	3.93E-05	5.47E-04
17454516	Psmg3	1.34	7.93E-05	9.42E-04
17245488	Tbk1	1.33	6.85E-05	8.32E-04
17503681	Cyld	1.33	1.10E-03	7.47E-03
17379864	1500012F01Rik	1.33	3.42E-05	4.91E-04
17344309	Tnf	1.33	6.71E-04	4.99E-03
17351634	Ccdc68	1.33	1.20E-04	1.31E-03
17456506	Snd1	1.33	4.05E-04	3.35E-03
17311286	Oxr1	1.33	9.27E-05	1.07E-03
17372984	Nup160	1.33	6.24E-04	4.72E-03
17332122	Tmem50b	1.33	1.19E-03	7.94E-03
17506306	Map1lc3b	1.33	5.23E-04	4.11E-03
17521762	Usp19	1.33	5.43E-04	4.24E-03
17297503	2310021P13Rik	1.33	4.01E-04	3.32E-03
17515715	Zbtb44	1.33	6.56E-04	4.91E-03
17341540	Tnfrsf12a	1.33	7.85E-04	5.67E-03
17232951	Sec63	1.33	3.36E-04	2.92E-03
17381630	Pfkfb3	1.33	1.39E-03	8.97E-03
17266146	Gosr1	1.33	7.34E-04	5.36E-03
17263893	Ulk2	1.33	1.13E-04	1.25E-03
17436583	Fam193a	1.33	7.26E-04	5.32E-03
17257591	Snord104	1.33	3.66E-04	3.11E-03
17322793	1810013L24Rik	1.32	6.59E-05	8.06E-04
17337314	Ppp1r10	1.32	1.46E-03	9.38E-03
17295960	Cwc27	1.32	3.02E-04	2.69E-03
17230668	Mia3	1.32	2.84E-04	2.57E-03
17535697	Ssr4	1.32	4.67E-05	6.22E-04
17467128	Nt5c3	1.32	1.30E-03	8.51E-03
17512275	Nae1	1.32	3.26E-04	2.84E-03

17296355	Itga2	1.32	3.36E-04	2.92E-03
17504727	Elmo3	1.32	9.07E-04	6.38E-03
17520815	Msl2	1.32	6.48E-04	4.87E-03
17475597	Sertad1	1.32	2.26E-04	2.16E-03
17316207	Trio	1.32	9.51E-04	6.63E-03
17522651	Clasp2	1.32	1.02E-03	7.03E-03
17219362	F11r	1.32	1.73E-04	1.75E-03
17407721	Gabpb2	1.32	1.39E-03	8.95E-03
17481960	Arntl	1.32	1.87E-04	1.86E-03
17410043	Mettl14	1.32	1.72E-04	1.74E-03
17277370	Eif2b2	1.32	4.01E-04	3.33E-03
17348250	Wac	1.32	1.08E-04	1.21E-03
17283131	Ptpn21	1.32	1.58E-03	9.98E-03
17244102	Scyl2	1.32	8.77E-05	1.03E-03
17413211	Vcp	1.31	2.76E-04	2.52E-03
17511450	Siah1a	1.31	2.92E-04	2.62E-03
17431562	Kdm1a	1.31	1.22E-03	8.09E-03
17317900	Gm10362	1.31	7.24E-04	5.31E-03
17248288	Sh3pxd2b	1.31	3.06E-04	2.71E-03
17234972	9130017N09Rik	1.31	1.04E-04	1.17E-03
17448324	1110003E01Rik	1.31	2.26E-04	2.15E-03
17424077	B4galt1	1.31	2.18E-04	2.10E-03
17474558	Ercc1	1.31	6.59E-04	4.93E-03
17265853	Pafah1b1	1.31	4.73E-04	3.80E-03
17512009	Csnk2a2	1.31	5.50E-04	4.28E-03
17525661	Tbrg1	1.31	1.20E-04	1.31E-03
17305856	Exoc5	1.31	5.04E-04	3.99E-03
17543336	Las1l	1.31	4.85E-04	3.88E-03
17520315	Plscr1	1.31	8.37E-04	5.97E-03
17543829	Rlim	1.31	7.38E-05	8.86E-04
17326859	Usp16	1.31	1.61E-04	1.65E-03
17267745	Tom1l1	1.31	3.74E-05	5.24E-04
17403681	Zzz3	1.31	3.11E-04	2.74E-03
17214998	Itm2c	1.31	7.03E-04	5.19E-03
17391332	Mal	1.31	6.70E-04	4.99E-03
17261107	Rel	1.31	8.40E-04	5.99E-03
17495414	Pik3c2a	1.31	9.67E-05	1.11E-03
17281529	Nemf	1.31	5.74E-04	4.43E-03
17245587	Mon2	1.31	1.83E-04	1.83E-03
17354969	Txnl1	1.31	2.55E-04	2.36E-03
17354263	Fem1c	1.31	1.21E-03	8.04E-03
17511453	N4bp1	1.31	8.95E-04	6.31E-03
17530128	Nck1	1.30	7.71E-04	5.58E-03
17410542	Nfkb1	1.30	1.60E-04	1.65E-03
17400317	Lass2	1.30	9.86E-04	6.83E-03

17228136	Lamc2	1.30	1.49E-03	9.52E-03
17259344	Hgs	1.30	8.38E-04	5.97E-03
17292817	Rab24	1.30	2.32E-04	2.20E-03
17466659	Tra2a	1.30	2.92E-04	2.62E-03
17483220	Cdipt	1.30	8.21E-04	5.87E-03
17459196	Tnip3	1.30	2.84E-04	2.57E-03
17267076	Ggnbp2	1.30	7.71E-05	9.20E-04
17428142	Osbpl9	1.30	1.98E-04	1.95E-03
17320887	Scaf11	1.30	6.23E-05	7.75E-04
17227384	Pkp1	1.30	1.75E-04	1.76E-03
17403340	Odf2l	1.30	2.95E-04	2.64E-03
17297080	Pxk	1.30	1.51E-04	1.58E-03
17515196	Qtrt1	1.30	1.05E-03	7.19E-03
17293089	Ubqln1	1.30	2.32E-04	2.20E-03
17426855	Ttc39b	1.30	7.47E-04	5.44E-03
17437390	Pi4k2b	1.30	1.35E-04	1.43E-03
17281148	Baz1a	1.30	9.73E-04	6.76E-03
17429933	Zc3h12a	1.30	8.12E-04	5.83E-03
17366093	Eif3a	1.30	8.30E-05	9.80E-04
17381330	Optn	1.30	2.78E-04	2.53E-03
17240945	Serinc1	1.30	8.09E-05	9.59E-04
17496283	Eif3c	1.30	5.05E-05	6.55E-04
17323945	Abcf3	1.30	1.42E-03	9.10E-03
17541926	Fgf13	1.30	4.41E-04	3.60E-03
17463973	Plekha5	1.30	4.88E-04	3.89E-03
17239227	Rab32	1.30	4.93E-04	3.92E-03
17423291	Esrp1	1.30	2.10E-04	2.04E-03
17496049	Nsmce1	1.30	5.91E-04	4.52E-03
17350447	Dmxl1	1.30	1.91E-04	1.89E-03
17443148	Cldn3	1.30	7.76E-05	9.25E-04
17468469	Dusp11	1.30	1.05E-03	7.18E-03
17374743	Gm14137	1.30	3.31E-04	2.88E-03
17381043	Tpd52l2	1.30	2.68E-04	2.46E-03
17239787	Enpp1	1.30	1.02E-03	7.02E-03
17283293	Gpr68	1.29	1.32E-03	8.60E-03
17323759	Ufd1l	1.29	3.10E-04	2.74E-03
17400599	Txnip	1.29	4.99E-05	6.50E-04
17327428	Dyrk1a	1.29	9.99E-04	6.90E-03
17380185	Rae1	1.29	1.35E-04	1.43E-03
17461177	Ppp4r2	1.29	4.08E-04	3.37E-03
17318907	Eif3d	1.29	4.85E-04	3.88E-03
17297462	Sec24c	1.29	2.72E-04	2.49E-03
17243661	Prdm4	1.29	1.72E-04	1.74E-03
17503226	Farsa	1.29	1.55E-03	9.81E-03
17467823	Kcmf1	1.29	2.30E-04	2.19E-03

17287185	Nol8	1.29	5.89E-04	4.51E-03
17277387	Fos	1.29	3.24E-04	2.83E-03
17227729	Cdc73	1.29	1.11E-03	7.53E-03
17385967	Galnt3	1.29	1.52E-04	1.58E-03
17229839	Gm20101	1.29	1.35E-03	8.77E-03
17448960	Clock	1.29	7.58E-04	5.50E-03
17490859	Nucb1	1.29	5.74E-04	4.43E-03
17338882	Dus3l	1.29	1.04E-03	7.15E-03
17326846	N6amt1	1.29	3.88E-04	3.25E-03
17290056	Mocs2	1.29	1.26E-03	8.27E-03
17402953	Ppp3ca	1.29	1.18E-03	7.92E-03
17315438	Prr13	1.29	9.37E-04	6.56E-03
17379128	Srsf6	1.28	7.10E-04	5.23E-03
17257331	Tlk2	1.28	3.01E-04	2.69E-03
17222642	Fhl2	1.28	8.74E-04	6.19E-03
17430140	Ncdn	1.28	5.60E-04	4.35E-03
17504160	Polr2c	1.28	1.39E-03	8.95E-03
17237170	Pawr	1.28	2.48E-04	2.31E-03
17212518	Wdr75	1.28	2.62E-04	2.42E-03
17237186	E2f7	1.28	1.25E-03	8.23E-03
17303400	Pdhb	1.28	9.09E-04	6.39E-03
17221490	Ube2w	1.28	8.93E-04	6.30E-03
17365421	Nt5c2	1.28	6.73E-04	5.01E-03
17353585	Sil1	1.28	1.78E-04	1.79E-03
17337441	Trim26	1.28	1.34E-03	8.72E-03
17390186	Tmem87a	1.28	1.47E-03	9.43E-03
17315810	Nipbl	1.28	1.39E-03	8.95E-03
17247993	Smek2	1.28	4.67E-04	3.77E-03
17353541	Etf1	1.28	5.53E-04	4.30E-03
17362869	AW112010	1.28	2.23E-04	2.14E-03
17540267	Gm14635	1.28	1.13E-03	7.60E-03
17326816	Gabpa	1.28	4.00E-04	3.32E-03
17510572	Med26	1.27	4.05E-04	3.35E-03
17315946	Dnajc21	1.27	1.02E-03	7.02E-03
17250812	Fam18b	1.27	2.44E-04	2.29E-03
17239113	Sash1	1.27	1.57E-03	9.91E-03
17396593	Sec62	1.27	6.65E-04	4.96E-03
17359055	Exoc6	1.27	8.57E-04	6.08E-03
17460622	Rpn1	1.27	3.40E-04	2.94E-03
17495223	Btbd10	1.27	9.88E-04	6.83E-03
17398571	Tmem154	1.27	4.77E-04	3.82E-03
17355962	Suv420h1	1.27	1.19E-03	7.94E-03
17534051	Il13ra1	1.27	8.94E-04	6.30E-03
17467730	Rnf181	1.27	7.38E-04	5.38E-03
17276884	4933426M11Rik	1.27	3.10E-04	2.74E-03

17357338	Mta2	1.27	3.63E-04	3.09E-03
17286231	Sox4	1.27	5.86E-04	4.50E-03
17436507	Whsc1	1.27	4.06E-04	3.36E-03
17312280	Gsdmd	1.27	2.75E-04	2.51E-03
17408813	Cttnbp2nl	1.27	7.32E-04	5.36E-03
17262326	Maml1	1.27	1.32E-03	8.61E-03
17466204	E330009J07Rik	1.27	5.99E-04	4.57E-03
17412860	Ubap1	1.27	1.33E-03	8.67E-03
17522761	Dync1li1	1.26	4.90E-04	3.90E-03
17227570	Nek7	1.26	5.92E-04	4.52E-03
17294805	Zcchc9	1.26	4.99E-04	3.96E-03
17397320	Larp1b	1.26	1.21E-03	8.05E-03
17349139	Sap130	1.26	2.80E-04	2.54E-03
17224661	Epha4	1.26	9.71E-04	6.75E-03
17229715	Ufc1	1.26	4.24E-04	3.48E-03
17234116	Ccdc6	1.26	8.91E-04	6.29E-03
17518664	Ppib	1.26	1.05E-03	7.21E-03
17504964	Edc4	1.26	4.64E-04	3.75E-03
17236415	Uhrf1bp1l	1.26	6.80E-04	5.05E-03
17364565	Blnk	1.26	7.18E-04	5.28E-03
17353554	Hspa9	1.26	2.68E-04	2.47E-03
17508300	Adam9	1.26	2.36E-04	2.23E-03
17231301	Cr1l	1.26	2.36E-04	2.23E-03
17231118	Rcor3	1.26	1.48E-03	9.45E-03
17410410	Sgms2	1.26	4.52E-04	3.68E-03
17248728	Clint1	1.25	6.47E-04	4.87E-03
17448415	Apbb2	1.25	8.80E-04	6.22E-03
17511259	Junb	1.25	1.11E-03	7.49E-03
17378692	Rpn2	1.25	4.95E-04	3.93E-03
17448335	Pds5a	1.25	9.93E-04	6.87E-03
17510563	Slc35e1	1.25	3.79E-04	3.19E-03
17334625	0610007P22Rik	1.25	6.58E-04	4.92E-03
17335616	Zfand3	1.25	1.20E-03	7.98E-03
17483501	Zfp646	1.25	9.41E-04	6.57E-03
17431362	Tmem57	1.25	3.64E-04	3.10E-03
17275015	Agr2	1.25	7.30E-04	5.34E-03
17441365	Taok3	1.25	8.53E-04	6.06E-03
17519509	Leo1	1.25	4.12E-04	3.40E-03
17382156	Abi1	1.25	2.45E-04	2.29E-03
17526349	Arcn1	1.25	7.51E-04	5.46E-03
17548446	Ormdl2	1.24	3.03E-04	2.69E-03
17392056	Fermt1	1.24	1.58E-03	9.98E-03
17398023	Gmps	1.24	1.46E-03	9.36E-03
17469802	Sec13	1.24	1.15E-03	7.71E-03
17407272	Ints3	1.24	4.75E-04	3.82E-03

17468511	Mxd1	1.24	6.66E-04	4.97E-03
17398287	Nmd3	1.24	1.50E-03	9.55E-03
17419995	Gale	1.24	6.06E-04	4.61E-03
17495262	Rras2	1.24	6.89E-04	5.10E-03
17230651	Brox	1.24	1.12E-03	7.56E-03
17361558	Sart1	1.24	1.03E-03	7.06E-03
17312946	Gm10863	1.23	1.33E-03	8.67E-03
17257051	Nmt1	1.23	1.10E-03	7.44E-03
17480078	Picalm	1.23	8.99E-04	6.33E-03
17305685	Ddhd1	1.22	1.05E-03	7.18E-03
17338796	Safb	1.22	9.92E-04	6.86E-03
17444338	Kdelr2	1.22	4.54E-04	3.69E-03
17225760	Stk25	1.22	1.01E-03	6.99E-03
17402181	F3	1.21	1.58E-03	9.98E-03
17385684	Rbms1	1.21	1.49E-03	9.52E-03
17231784	Cited2	1.21	1.49E-03	9.52E-03
17506603	Tcf25	1.20	8.92E-04	6.29E-03
17436077	Nrbp1	1.20	1.49E-03	9.52E-03
17365134	Ndufb8	0.84	1.23E-03	8.11E-03
17384632	Strbp	0.84	1.28E-03	8.37E-03
17319619	Ndufa6	0.83	1.11E-03	7.51E-03
17374098	Lgr4	0.83	9.86E-04	6.83E-03
17212080	Pdcl3	0.83	1.55E-03	9.81E-03
17505774	Gabarapl2	0.83	1.59E-03	9.98E-03
17255157	Nme1	0.83	1.02E-03	7.04E-03
17234926	Bsg	0.83	8.76E-04	6.20E-03
17229665	Ndufs2	0.82	7.91E-04	5.70E-03
17334932	Ergic1	0.82	1.54E-03	9.79E-03
17332860	Tulp4	0.82	1.14E-03	7.69E-03
17374089	Lin7c	0.82	7.70E-04	5.57E-03
17480813	Stard10	0.82	1.47E-03	9.40E-03
17335842	Pde9a	0.82	9.99E-04	6.90E-03
17233629	Psap	0.82	1.47E-03	9.39E-03
17517116	Timm8b	0.82	7.82E-04	5.65E-03
17489620	Gpi1	0.82	1.21E-03	8.04E-03
17493526	Capn5	0.82	6.54E-04	4.91E-03
17282732	0610007P14Rik	0.81	5.37E-04	4.20E-03
17263004	Atox1	0.81	1.38E-03	8.92E-03
17367686	Il1rn	0.81	6.48E-04	4.87E-03
17449804	Ccni	0.81	6.59E-04	4.92E-03
17310912	Laptm4b	0.81	1.57E-03	9.92E-03
17482730	Dctn5	0.81	1.10E-03	7.47E-03
17374218	Lpcat4	0.81	7.41E-04	5.41E-03
17361056	Ndufv1	0.81	1.28E-03	8.39E-03
17311315	Ttc35	0.81	1.56E-03	9.90E-03

17386396	Slc25a12	0.81	6.88E-04	5.10E-03
17503942	Mt1	0.81	1.30E-03	8.51E-03
17243014	Fam108a	0.81	1.56E-03	9.90E-03
17470879	Tpi1	0.81	1.55E-03	9.81E-03
17312396	Cyc1	0.81	4.00E-04	3.32E-03
17396750	Ndufb5	0.81	1.36E-03	8.84E-03
17328288	Pdxdc1	0.81	1.36E-03	8.85E-03
17334262	Pgp	0.81	8.52E-04	6.05E-03
17261098	Pex13	0.81	7.60E-04	5.52E-03
17223283	Satb2	0.81	6.69E-04	4.98E-03
17534112	Slc25a5	0.81	7.82E-04	5.65E-03
17538096	Rnf128	0.81	4.91E-04	3.90E-03
17238571	Dnajc14	0.81	1.12E-03	7.57E-03
17463056	Chd4	0.81	1.05E-03	7.18E-03
17366039	Pdzd8	0.81	1.09E-03	7.44E-03
17214665	Sgpp2	0.80	1.53E-03	9.74E-03
17216836	R3hdm1	0.80	1.58E-03	9.98E-03
17501050	Irf2	0.80	1.08E-03	7.37E-03
17449313	Ugt2b34	0.80	1.08E-03	7.35E-03
17379808	Cse1l	0.80	1.18E-03	7.91E-03
17465671	Chchd3	0.80	1.22E-03	8.09E-03
17523555	Exosc7	0.80	5.26E-04	4.13E-03
17401500	Dram2	0.80	5.42E-04	4.23E-03
17530742	Tex264	0.80	1.49E-03	9.50E-03
17498461	Fadd	0.80	1.26E-03	8.30E-03
17379439	Pigt	0.80	5.84E-04	4.49E-03
17296013	Ipo11	0.80	5.59E-04	4.34E-03
17471166	Ndufa9	0.80	8.41E-04	5.99E-03
17454069	Mospd3	0.80	8.45E-04	6.01E-03
17542382	Bcap31	0.80	3.48E-04	2.99E-03
17542853	Pls3	0.80	1.01E-03	6.94E-03
17256959	Grn	0.80	8.12E-04	5.83E-03
17300429	Dhrs4	0.80	9.74E-04	6.76E-03
17355169	Afg3l2	0.80	1.17E-03	7.84E-03
17247695	Xpo1	0.80	1.03E-03	7.09E-03
17235268	Ndufs7	0.80	9.60E-04	6.69E-03
17216436	Serpinb11	0.80	1.21E-03	8.04E-03
17350140	Tcerg1	0.80	6.18E-04	4.69E-03
17326266	Senp7	0.80	1.44E-03	9.26E-03
17425533	Epb4.1l4b	0.80	7.01E-04	5.18E-03
17484881	Taldo1	0.80	1.52E-03	9.68E-03
17527934	Kif23	0.80	6.36E-04	4.80E-03
17535607	Slc6a8	0.80	3.36E-04	2.91E-03
17525288	Tmem45b	0.79	2.51E-04	2.33E-03
17362156	Plcb3	0.79	6.77E-04	5.03E-03

17502944	Ndufb7	0.79	2.75E-04	2.51E-03
17320907	Slc38a1	0.79	1.12E-03	7.58E-03
17331669	App	0.79	2.89E-04	2.60E-03
17406867	Dap3	0.79	1.22E-03	8.09E-03
17501427	Cbr4	0.79	1.09E-03	7.42E-03
17510057	Klhl26	0.79	8.31E-04	5.93E-03
17512666	Smpd3	0.79	6.85E-04	5.08E-03
17233273	Dcbld1	0.79	9.04E-04	6.37E-03
17526813	Pts	0.79	9.37E-04	6.56E-03
17377418	Cst3	0.79	4.23E-04	3.47E-03
17439037	Parm1	0.79	3.77E-04	3.18E-03
17362197	Vegfb	0.79	3.76E-04	3.18E-03
17417738	Hyi	0.79	6.70E-04	4.99E-03
17525955	Sc5d	0.79	2.77E-04	2.52E-03
17431377	Tmem50a	0.79	1.23E-03	8.13E-03
17491115	Ush1c	0.79	1.21E-03	8.05E-03
17391971	Pcna	0.79	1.54E-04	1.60E-03
17548068	LOC100504825	0.79	5.45E-04	4.25E-03
17462926	Phb2	0.79	6.13E-04	4.66E-03
17390997	Secisbp2l	0.79	5.58E-04	4.33E-03
17377126	Dtd1	0.79	5.22E-04	4.11E-03
17527532	Mpi	0.79	7.86E-04	5.67E-03
17539415	Rbbp7	0.79	4.64E-04	3.75E-03
17289717	Elovl7	0.79	2.75E-04	2.51E-03
17342414	Rhot2	0.79	8.84E-04	6.25E-03
17497741	Ano9	0.79	1.56E-03	9.90E-03
17502527	Isx	0.79	2.50E-04	2.32E-03
17400759	Acp6	0.79	5.43E-04	4.23E-03
17214234	Vil1	0.78	2.24E-04	2.14E-03
17418904	Ak2	0.78	1.31E-03	8.57E-03
17317086	Taf2	0.78	7.12E-04	5.24E-03
17416427	Tmem59	0.78	5.20E-04	4.10E-03
17255828	Mllt6	0.78	6.76E-04	5.02E-03
17306785	Nedd8	0.78	1.63E-04	1.67E-03
17470445	Atp6v1e1	0.78	2.70E-04	2.48E-03
17358690	Papss2	0.78	1.42E-03	9.12E-03
17401463	1810022K09Rik	0.78	2.40E-04	2.25E-03
17360501	Tcf7l2	0.78	2.52E-04	2.34E-03
17483486	Stx4a	0.78	3.89E-04	3.25E-03
17246131	Obfc2b	0.78	3.61E-04	3.08E-03
17533282	Mid1ip1	0.78	2.75E-04	2.51E-03
17327660	Trap1	0.78	1.25E-03	8.23E-03
17238482	Rnf41	0.78	1.07E-03	7.29E-03
17443231	Baz1b	0.78	2.40E-04	2.25E-03
17314679	Tuba1b	0.78	3.62E-04	3.08E-03

17492847	Btbd1	0.78	8.63E-04	6.12E-03
17440923	Ube3b	0.78	1.24E-03	8.21E-03
17442149	P2rx4	0.78	1.35E-03	8.74E-03
17258547	2310004N24Rik	0.78	1.19E-03	7.97E-03
17234436	Chchd10	0.78	1.37E-03	8.86E-03
17400403	Golph3l	0.78	6.02E-04	4.59E-03
17258131	Slc9a3r1	0.78	2.60E-04	2.40E-03
17309293	Rnf219	0.78	7.31E-04	5.35E-03
17266038	Fam101b	0.78	2.46E-04	2.30E-03
17388353	Gyltl1b	0.78	1.41E-03	9.07E-03
17456285	Naa38	0.78	3.97E-04	3.31E-03
17484382	Inpp5a	0.78	6.41E-04	4.83E-03
17252113	Mink1	0.78	4.50E-04	3.66E-03
17435189	Mll5	0.78	4.17E-04	3.43E-03
17381779	Rsu1	0.78	1.80E-04	1.80E-03
17470988	Ptms	0.78	4.72E-04	3.80E-03
17361921	Tm7sf2	0.78	1.33E-03	8.70E-03
17541383	Zdhhc9	0.78	5.91E-04	4.52E-03
17424023	Ndufb6	0.78	1.34E-04	1.43E-03
17311796	Ndufb9	0.78	3.25E-04	2.84E-03
17224500	Atg9a	0.77	5.91E-04	4.52E-03
17363704	Rfx3	0.77	9.55E-04	6.65E-03
17244514	Nudt4	0.77	3.42E-04	2.95E-03
17503756	Rbl2	0.77	3.30E-04	2.87E-03
17310432	Mtmr12	0.77	1.44E-03	9.23E-03
17406091	Etfdh	0.77	4.27E-04	3.50E-03
17497877	B230206H07Rik	0.77	8.18E-04	5.86E-03
17422587	Nadk	0.77	6.44E-04	4.85E-03
17464626	Pon3	0.77	9.37E-04	6.56E-03
17219554	Igsf9	0.77	2.20E-04	2.12E-03
17430835	Phactr4	0.77	5.65E-04	4.37E-03
17503287	Prdx2	0.77	1.23E-04	1.33E-03
17228751	Rabgap1l	0.77	1.16E-03	7.78E-03
17411174	Gipc2	0.77	3.54E-04	3.03E-03
17405133	Ndufc1	0.77	4.21E-04	3.46E-03
17456053	Tmem106b	0.77	2.79E-04	2.53E-03
17347680	Zfp36l2	0.77	1.10E-03	7.44E-03
17281908	Tmem30b	0.77	2.50E-04	2.32E-03
17235198	Atp5d	0.77	9.14E-05	1.06E-03
17220255	Cnih4	0.77	1.80E-04	1.80E-03
17382802	Ubac1	0.77	1.93E-04	1.91E-03
17534408	Xiap	0.77	6.46E-04	4.86E-03
17249440	Vdac1	0.77	1.87E-04	1.86E-03
17430878	Atpif1	0.77	8.14E-05	9.62E-04
17426206	Alad	0.77	1.51E-04	1.57E-03

17286595	Rreb1	0.77	9.07E-04	6.38E-03
17384587	Rc3h2	0.77	3.22E-04	2.82E-03
17508885	Mtmr7	0.77	1.64E-04	1.68E-03
17420347	Ece1	0.77	1.94E-04	1.91E-03
17495466	Arl6ip1	0.77	2.04E-04	1.99E-03
17407201	Ubap2l	0.77	1.24E-04	1.34E-03
17526583	Sidt2	0.77	1.20E-03	7.98E-03
17404585	Zmat3	0.77	5.27E-04	4.13E-03
17295907	Nln	0.77	5.78E-04	4.45E-03
17245044	Tmem19	0.77	1.14E-03	7.69E-03
17222326	6330578E17Rik	0.77	1.37E-03	8.89E-03
17399474	Pmvk	0.77	8.58E-04	6.08E-03
17414456	Hsdl2	0.77	2.31E-04	2.19E-03
17319465	St13	0.77	6.52E-05	8.01E-04
17529713	2610101N10Rik	0.77	7.55E-05	9.04E-04
17325581	Cox17	0.77	2.16E-04	2.08E-03
17295038	Scamp1	0.77	6.15E-04	4.67E-03
17495586	Gde1	0.77	1.59E-03	9.98E-03
17368550	Rxra	0.77	5.46E-04	4.25E-03
17313394	Aco2	0.77	3.53E-04	3.02E-03
17471342	Fkbp4	0.77	5.81E-04	4.47E-03
17495553	Coq7	0.77	6.01E-05	7.55E-04
17503031	Prkaca	0.77	7.75E-04	5.61E-03
17533474	Maoa	0.77	4.69E-04	3.78E-03
17299703	G630016D24Rik	0.77	5.33E-04	4.17E-03
17413528	Melk	0.77	2.97E-04	2.66E-03
17238722	H60c	0.77	1.21E-03	8.04E-03
17307354	Atp8a2	0.77	5.15E-04	4.06E-03
17412730	Aco1	0.77	1.04E-04	1.17E-03
17427389	Cyp2j6	0.76	1.34E-03	8.72E-03
17266157	Cpd	0.76	7.29E-04	5.34E-03
17330323	Nr1i2	0.76	1.30E-03	8.49E-03
17503507	Lonp2	0.76	9.10E-05	1.06E-03
17420604	Akr7a5	0.76	1.21E-03	8.04E-03
17360695	Atrnl1	0.76	6.57E-04	4.92E-03
17532418	Ano10	0.76	1.27E-03	8.35E-03
17225586	Myeov2	0.76	2.16E-04	2.08E-03
17315635	Ghr	0.76	1.41E-03	9.05E-03
17515413	Pigyl	0.76	1.44E-03	9.22E-03
17246381	Mettl7b	0.76	1.14E-03	7.66E-03
17498502	Ccnd1	0.76	7.60E-05	9.09E-04
17407764	Prune	0.76	1.11E-03	7.53E-03
17288908	Cetn3	0.76	6.27E-04	4.74E-03
17510906	Tecr	0.76	3.18E-05	4.64E-04
17344456	2310061I04Rik	0.76	2.39E-04	2.24E-03

17261035	Ahsa2	0.76	5.55E-04	4.31E-03
17304518	2010107H07Rik	0.76	5.78E-04	4.45E-03
17383985	Ttc16	0.76	9.46E-04	6.60E-03
17219242	Apoa2	0.76	6.82E-04	5.06E-03
17210984	Pcmtd1	0.76	2.32E-04	2.20E-03
17513681	Mvd	0.76	8.89E-04	6.28E-03
17543664	Cited1	0.76	1.12E-03	7.54E-03
17229417	Pbx1	0.76	1.18E-03	7.91E-03
17291374	Mrs2	0.76	1.21E-03	8.02E-03
17379560	Ctsa	0.76	1.45E-04	1.52E-03
17408863	Atp5f1	0.76	3.81E-04	3.20E-03
17512342	Tradd	0.76	5.11E-04	4.04E-03
17499310	Tfdp1	0.76	3.46E-04	2.98E-03
17246310	Dgka	0.76	6.49E-04	4.87E-03
17501148	Aga	0.76	2.14E-04	2.07E-03
17372045	Hoxd11	0.76	1.05E-03	7.20E-03
17308603	Itm2b	0.76	2.19E-05	3.47E-04
17502626	Nr3c2	0.76	1.34E-03	8.71E-03
17322637	Gm5480	0.76	8.05E-05	9.54E-04
17265129	Acadvl	0.76	1.20E-04	1.31E-03
17389901	Exd1	0.76	5.98E-04	4.57E-03
17513311	2310061C15Rik	0.76	2.56E-04	2.37E-03
17420897	Sdhb	0.75	1.38E-04	1.46E-03
17451536	Mmab	0.75	1.47E-03	9.41E-03
17427686	Slc35d1	0.75	1.27E-04	1.37E-03
17526674	Rexo2	0.75	1.69E-04	1.72E-03
17286055	Gpld1	0.75	2.49E-04	2.32E-03
17409893	Slc44a3	0.75	1.29E-03	8.44E-03
17249811	Slc36a1	0.75	1.35E-03	8.76E-03
17251634	A030009H04Rik	0.75	1.95E-04	1.92E-03
17249801	Gm2a	0.75	1.54E-03	9.76E-03
17313619	Serhl	0.75	2.82E-04	2.56E-03
17232977	Scml4	0.75	5.01E-04	3.97E-03
17265186	Rnasek	0.75	2.49E-04	2.32E-03
17234552	Lss	0.75	4.47E-04	3.64E-03
17216727	Slc35f5	0.75	5.44E-05	6.96E-04
17253616	Pigs	0.75	1.93E-04	1.91E-03
17371739	Hat1	0.75	7.02E-04	5.18E-03
17393095	Sun5	0.75	1.58E-04	1.63E-03
17418101	Ppt1	0.75	9.87E-05	1.12E-03
17301899	Sucla2	0.75	1.02E-04	1.16E-03
17402283	Gclm	0.75	1.95E-04	1.92E-03
17336636	Agpat1	0.75	8.01E-05	9.51E-04
17342581	Decr2	0.75	5.81E-04	4.47E-03
17318877	Txn2	0.75	9.37E-04	6.56E-03

17402321	Pde5a	0.75	1.03E-03	7.09E-03
17269911	Vat1	0.75	3.26E-04	2.84E-03
17415219	Acer2	0.75	9.65E-04	6.71E-03
17280897	Stxbp6	0.75	1.02E-03	7.02E-03
17261840	Hmmr	0.75	7.63E-04	5.54E-03
17428735	Urod	0.75	3.03E-04	2.69E-03
17456152	Cav2	0.75	7.65E-04	5.55E-03
17263535	Srebf1	0.75	2.21E-04	2.12E-03
17532455	1110059G10Rik	0.75	1.39E-03	8.97E-03
17230735	Mosc2	0.75	1.11E-04	1.23E-03
17538852	Hsd17b10	0.75	6.00E-04	4.58E-03
17215647	Prlh	0.75	4.86E-04	3.88E-03
17323440	Tmem191c	0.75	9.38E-04	6.56E-03
17506030	Hsd17b2	0.75	8.51E-05	9.99E-04
17313739	Prr5	0.75	6.21E-04	4.71E-03
17370273	Ndufa8	0.75	1.66E-04	1.70E-03
17214904	C130026I21Rik	0.75	2.60E-04	2.40E-03
17489768	Tdrd12	0.75	8.19E-04	5.87E-03
17406656	Apoa1bp	0.75	1.45E-04	1.52E-03
17255727	Osbpl7	0.75	5.17E-04	4.08E-03
17465201	Ndufa5	0.75	9.45E-05	1.09E-03
17242924	Uqcr11	0.75	1.10E-03	7.48E-03
17312511	Adck5	0.75	7.26E-04	5.32E-03
17253996	Tmem98	0.75	3.82E-04	3.20E-03
17450142	Hpse	0.75	2.12E-04	2.05E-03
17277294	Isca2	0.75	8.42E-05	9.91E-04
17435455	2900005J15Rik	0.75	4.71E-04	3.79E-03
17336077	Zfp955b	0.75	5.38E-04	4.21E-03
17398272	Arl14	0.75	4.34E-04	3.55E-03
17477116	Etfb	0.75	5.48E-05	7.00E-04
17456604	Ccdc136	0.75	3.16E-04	2.78E-03
17267430	Gdpd1	0.75	2.05E-04	2.00E-03
17374569	Bub1b	0.75	1.58E-03	9.98E-03
17342770	Spdef	0.74	4.90E-04	3.90E-03
17542419	L1cam	0.74	1.33E-03	8.70E-03
17502816	Inpp4b	0.74	4.20E-04	3.45E-03
17521996	Uqcrc1	0.74	5.09E-05	6.57E-04
17411547	Chchd7	0.74	9.24E-04	6.49E-03
17447310	Lrpap1	0.74	3.01E-04	2.69E-03
17323589	Klhl22	0.74	9.40E-04	6.57E-03
17247519	Spred2	0.74	3.71E-04	3.14E-03
17238605	Itga7	0.74	4.64E-04	3.75E-03
17276743	Plekhh1	0.74	2.32E-04	2.20E-03
17373984	Immp1l	0.74	5.46E-04	4.25E-03
17347114	Capn13	0.74	1.11E-03	7.51E-03

17303757	Dnajc9	0.74	2.45E-04	2.29E-03
17249425	Skp1a	0.74	3.91E-04	3.26E-03
17447128	Slbp	0.74	1.41E-03	9.06E-03
17286927	Jarid2	0.74	8.21E-04	5.87E-03
17305967	Ttc5	0.74	6.32E-04	4.78E-03
17217127	Nucks1	0.74	1.06E-04	1.19E-03
17464040	Pde3a	0.74	7.51E-04	5.46E-03
17262516	0610009B22Rik	0.74	3.31E-04	2.88E-03
17347751	Lrpprc	0.74	1.42E-04	1.50E-03
17493298	Tmem126b	0.74	7.03E-04	5.19E-03
17399519	Ube2q1	0.74	6.22E-04	4.71E-03
17424163	Ubap2	0.74	8.92E-05	1.04E-03
17448832	Lrrc66	0.74	1.99E-04	1.95E-03
17439563	Enoph1	0.74	2.86E-04	2.58E-03
17413852	Galnt12	0.74	8.06E-04	5.80E-03
17468157	Tet3	0.74	3.09E-04	2.74E-03
17213338	Fam117b	0.74	2.21E-04	2.12E-03
17468798	Mcm2	0.74	2.45E-04	2.29E-03
17440826	Acacb	0.74	7.34E-04	5.36E-03
17235379	Csnk1g2	0.74	3.21E-05	4.68E-04
17389836	Ppp1r14d	0.74	6.95E-05	8.41E-04
17305163	2610528A11Rik	0.74	4.63E-05	6.18E-04
17328829	Slc25a1	0.74	5.67E-05	7.17E-04
17403126	Adh5	0.74	2.77E-04	2.52E-03
17413352	Car9	0.74	4.63E-05	6.18E-04
17265422	C1qbp	0.74	7.92E-04	5.70E-03
17394829	Atp9a	0.74	1.53E-04	1.59E-03
17243034	Mknk2	0.74	1.20E-04	1.31E-03
17518172	Anp32a	0.74	2.40E-05	3.73E-04
17504293	Mmp15	0.74	5.83E-05	7.34E-04
17343035	Mtch1	0.74	2.12E-05	3.38E-04
17213990	Atic	0.74	3.41E-04	2.95E-03
17214883	Fbxo36	0.74	4.37E-04	3.57E-03
17548428	Uqcrb	0.74	3.45E-04	2.97E-03
17402025	Dbt	0.74	2.84E-04	2.57E-03
17527735	Neo1	0.74	3.72E-04	3.15E-03
17455882	Ppp1r9a	0.74	1.59E-03	9.98E-03
17495985	Gm20299	0.74	8.06E-04	5.80E-03
17339108	Man2a1	0.74	2.28E-04	2.17E-03
17342595	Nme4	0.74	1.39E-03	8.99E-03
17530159	Ppp2r3a	0.74	1.07E-03	7.31E-03
17462905	Lpcat3	0.74	3.72E-05	5.22E-04
17312551	Ppp1r16a	0.74	5.36E-05	6.87E-04
17241623	Reep3	0.74	7.23E-04	5.30E-03
17241228	Eif4ebp2	0.74	3.84E-05	5.37E-04

17280086	Greb1	0.74	4.64E-04	3.75E-03
17526363	Tmem25	0.74	4.64E-04	3.75E-03
17289455	Serf1	0.74	2.84E-04	2.57E-03
17506479	Spg7	0.74	6.35E-04	4.80E-03
17282104	Atp6v1d	0.73	1.17E-04	1.29E-03
17397129	Fgf2	0.73	1.63E-04	1.67E-03
17261865	Ccng1	0.73	2.15E-05	3.43E-04
17354764	Arhgef37	0.73	8.09E-05	9.58E-04
17512716	Terf2	0.73	6.64E-04	4.96E-03
17321645	Lima1	0.73	4.21E-05	5.74E-04
17225564	Ndufa10	0.73	4.73E-05	6.27E-04
17362216	Nudt22	0.73	5.99E-04	4.57E-03
17386135	Stk39	0.73	1.39E-03	8.99E-03
17350740	Lmnb1	0.73	1.20E-04	1.31E-03
17367036	Pter	0.73	2.91E-04	2.62E-03
17511389	Itfg1	0.73	3.09E-05	4.54E-04
17536600	Gdpd2	0.73	1.22E-03	8.10E-03
17338445	Unc5cl	0.73	7.50E-04	5.45E-03
17291767	Tubb2a	0.73	1.50E-04	1.57E-03
17249362	Sar1b	0.73	1.06E-04	1.18E-03
17502011	Gm3336	0.73	2.40E-04	2.25E-03
17225775	Thap4	0.73	2.90E-05	4.32E-04
17230174	Chml	0.73	1.41E-03	9.05E-03
17531168	Dag1	0.73	1.82E-04	1.82E-03
17281063	6530401N04Rik	0.73	8.07E-04	5.80E-03
17537190	Sh3bgrl	0.73	5.70E-04	4.41E-03
17253630	Unc119	0.73	1.54E-03	9.79E-03
17428217	Cdkn2c	0.73	9.87E-04	6.83E-03
17451972	Plbd2	0.73	2.13E-04	2.06E-03
17391608	Idh3b	0.73	7.31E-05	8.79E-04
17398180	Mfsd1	0.73	3.26E-04	2.85E-03
17526861	Dlat	0.73	6.97E-05	8.43E-04
17421120	Casp9	0.73	2.59E-04	2.39E-03
17541618	Mbnl3	0.73	2.97E-04	2.66E-03
17264562	Cntrob	0.73	3.69E-04	3.13E-03
17480216	Prcp	0.73	1.21E-04	1.32E-03
17527353	Etfa	0.73	2.01E-05	3.24E-04
17469879	Timp4	0.73	1.27E-03	8.33E-03
17447904	Qdpr	0.73	4.44E-04	3.62E-03
17468551	Anxa4	0.73	8.06E-06	1.54E-04
17260357	H2afv	0.73	1.06E-03	7.27E-03
17222332	Mgat4a	0.73	2.13E-05	3.39E-04
17251994	Slc16a11	0.73	2.33E-04	2.20E-03
17475251	Megf8	0.73	4.50E-05	6.04E-04
17497595	1810014F10Rik	0.73	1.42E-03	9.12E-03

17304203	Slmap	0.73	1.85E-05	3.04E-04
17409463	Fam102b	0.73	1.69E-04	1.72E-03
17291570	Uqcrfs1	0.73	4.14E-05	5.68E-04
17439557	Hnrnpd	0.73	1.30E-03	8.49E-03
17485339	Slc22a18	0.73	1.05E-05	1.92E-04
17502729	Zfp827	0.73	3.05E-04	2.71E-03
17538297	Tmem164	0.72	8.76E-04	6.20E-03
17515340	Rab3d	0.72	1.22E-04	1.32E-03
17467154	Ppm1k	0.72	3.14E-04	2.76E-03
17527678	Pml	0.72	2.77E-04	2.52E-03
17468961	Nup210	0.72	1.53E-04	1.59E-03
17360312	Add3	0.72	5.23E-04	4.11E-03
17288463	Mrpl36	0.72	1.01E-03	6.96E-03
17433631	Prdm16	0.72	3.27E-04	2.85E-03
17325637	B4galt4	0.72	5.93E-04	4.53E-03
17523281	Trak1	0.72	1.21E-04	1.32E-03
17409920	Abcd3	0.72	4.91E-05	6.43E-04
17300984	Cab39l	0.72	2.74E-04	2.50E-03
17329937	Umps	0.72	3.44E-04	2.97E-03
17409753	Agl	0.72	1.06E-04	1.18E-03
17518636	2810417H13Rik	0.72	1.21E-03	8.04E-03
17520932	Topbp1	0.72	3.50E-05	4.98E-04
17307588	Fdft1	0.72	1.51E-05	2.58E-04
17423701	Fut9	0.72	5.85E-05	7.36E-04
17402595	Casp6	0.72	4.93E-05	6.44E-04
17375767	Blvra	0.72	3.24E-04	2.83E-03
17342625	Itfg3	0.72	1.26E-03	8.28E-03
17359531	Marveld1	0.72	1.26E-03	8.28E-03
17247471	C1d	0.72	5.64E-05	7.15E-04
17455507	Hsph1	0.72	1.08E-03	7.37E-03
17343735	H2-Ke6	0.72	3.77E-04	3.18E-03
17535930	Dkc1	0.72	4.03E-04	3.34E-03
17493185	Tmem135	0.72	3.99E-04	3.31E-03
17256033	Grb7	0.72	7.93E-05	9.42E-04
17373086	Mtch2	0.72	3.21E-05	4.68E-04
17424515	Fancg	0.72	2.98E-04	2.66E-03
17240901	D630037F22Rik	0.72	4.59E-04	3.73E-03
17453383	Clip2	0.72	1.57E-04	1.62E-03
17284222	Ahnak2	0.72	5.08E-04	4.02E-03
17509537	Palld	0.72	2.23E-04	2.14E-03
17300672	Nfatc4	0.72	8.57E-04	6.08E-03
17537895	Ngfrap1	0.72	2.23E-04	2.13E-03
17491259	Uevld	0.72	5.08E-05	6.57E-04
17327544	Bace2	0.72	2.16E-04	2.08E-03
17431867	Minos1	0.72	3.66E-04	3.11E-03

17258287	2310067B10Rik	0.72	2.34E-04	2.21E-03
17229629	Sdhc	0.72	5.62E-05	7.14E-04
17337142	H2-Q10	0.72	7.02E-04	5.19E-03
17402756	Ppa2	0.72	2.25E-04	2.15E-03
17531834	Fbxl2	0.72	4.65E-04	3.76E-03
17282117	Plek2	0.72	1.31E-05	2.31E-04
17399783	S100a16	0.72	1.17E-03	7.81E-03
17241912	Ddt	0.72	7.52E-04	5.46E-03
17331059	Gpr128	0.72	1.62E-05	2.76E-04
17376917	2310003L22Rik	0.72	9.43E-04	6.59E-03
17510129	Mpv17l2	0.72	3.12E-05	4.57E-04
17319554	Pmm1	0.72	1.25E-03	8.25E-03
17287414	Sfxn1	0.72	1.20E-04	1.31E-03
17546222	Tmlhe	0.72	3.80E-04	3.20E-03
17369555	Abl1	0.72	8.25E-04	5.89E-03
17468933	Iqsec1	0.72	2.09E-04	2.03E-03
17493632	Dgat2	0.71	1.29E-04	1.39E-03
17294377	Sdha	0.71	1.60E-05	2.72E-04
17404329	Gyg	0.71	2.94E-04	2.64E-03
17216158	Fam174a	0.71	5.42E-04	4.23E-03
17242879	Adamtsl5	0.71	1.04E-04	1.18E-03
17514732	Sesn3	0.71	3.37E-04	2.92E-03
17229403	Mgst3	0.71	1.31E-05	2.30E-04
17272262	Evpl	0.71	4.41E-04	3.60E-03
17221633	Mcm3	0.71	6.56E-04	4.91E-03
17272241	Acox1	0.71	2.50E-05	3.84E-04
17321263	Adcy6	0.71	1.18E-04	1.29E-03
17409981	4930447N08Rik	0.71	9.81E-04	6.80E-03
17477979	Dbp	0.71	9.18E-04	6.45E-03
17459038	Herc3	0.71	5.36E-04	4.20E-03
17348356	Snrpd1	0.71	8.18E-04	5.86E-03
17482739	Plk1	0.71	2.06E-05	3.31E-04
17510120	Pgpep1	0.71	8.51E-04	6.05E-03
17516030	Tmem218	0.71	6.19E-04	4.69E-03
17412067	Fam82b	0.71	1.39E-03	8.97E-03
17501609	Ints10	0.71	2.08E-04	2.02E-03
17287772	Pcbd2	0.71	3.52E-05	5.00E-04
17417269	4732418C07Rik	0.71	1.30E-04	1.40E-03
17480568	Arrb1	0.71	9.66E-05	1.11E-03
17377666	Srxn1	0.71	1.54E-04	1.60E-03
17545910	Grpr	0.71	1.57E-03	9.95E-03
17532030	Cmc1	0.71	4.94E-05	6.45E-04
17498370	Nadsyn1	0.71	4.87E-04	3.89E-03
17241692	Cdk1	0.71	2.76E-04	2.52E-03
17422117	Acot7	0.71	3.98E-04	3.31E-03

17537889	Wbp5	0.71	8.71E-04	6.17E-03
17292084	9530008L14Rik	0.71	9.35E-05	1.08E-03
17295796	Pik3r1	0.71	9.94E-05	1.13E-03
17403514	Ssx2ip	0.71	9.32E-05	1.08E-03
17400048	Rorc	0.71	1.06E-03	7.23E-03
17334205	Abca3	0.71	2.25E-04	2.15E-03
17539467	Ap1s2	0.71	1.27E-03	8.36E-03
17384334	Phf19	0.71	1.50E-03	9.55E-03
17458327	Repin1	0.71	2.80E-05	4.21E-04
17475026	Ethe1	0.71	2.04E-05	3.29E-04
17225627	9430060I03Rik	0.71	8.04E-04	5.78E-03
17417107	9130206I24Rik	0.71	1.57E-04	1.63E-03
17446587	Lmbr1	0.71	1.59E-04	1.64E-03
17508464	4930444A02Rik	0.71	7.50E-04	5.45E-03
17392730	Abhd12	0.71	5.03E-05	6.55E-04
17396315	Tbl1xr1	0.71	1.89E-04	1.87E-03
17347475	Hnrpll	0.71	7.62E-05	9.10E-04
17450741	Atp5k	0.71	8.28E-06	1.58E-04
17260944	Mdh1	0.71	2.39E-05	3.71E-04
17327236	Clic6	0.71	6.14E-04	4.67E-03
17404750	Mccc1	0.71	2.10E-04	2.03E-03
17484068	Lhpp	0.71	1.59E-03	9.98E-03
17282563	Npc2	0.71	1.10E-05	2.01E-04
17337719	Mut	0.71	4.87E-04	3.89E-03
17455307	Cdx2	0.71	4.15E-04	3.42E-03
17334922	Neurl1b	0.71	1.17E-03	7.81E-03
17409721	Hiat1	0.71	3.86E-04	3.23E-03
17268817	LOC100862101	0.71	2.35E-05	3.68E-04
17325159	Mylk	0.71	1.19E-04	1.30E-03
17423037	Impad1	0.71	2.24E-04	2.15E-03
17257444	Ace	0.71	7.49E-04	5.45E-03
17439766	LOC100861848	0.71	4.86E-05	6.38E-04
17519778	LOC100862063	0.71	7.01E-06	1.38E-04
17510145	Pik3r2	0.70	1.22E-03	8.09E-03
17515641	AI414108	0.70	1.25E-03	8.25E-03
17277195	Ptgr2	0.70	4.85E-04	3.88E-03
17467166	Pigy	0.70	3.40E-04	2.94E-03
17446643	Hadha	0.70	6.08E-05	7.60E-04
17396439	Tnik	0.70	3.44E-04	2.97E-03
17381573	Atp5c1	0.70	7.25E-05	8.73E-04
17213548	Nrp2	0.70	4.57E-05	6.12E-04
17310837	Sema5a	0.70	1.21E-04	1.32E-03
17243638	Mterfd3	0.70	7.04E-04	5.20E-03
17535627	Abcd1	0.70	1.38E-03	8.95E-03
17348933	Mapre2	0.70	3.59E-05	5.07E-04

17217136	Slc45a3	0.70	4.22E-04	3.46E-03
17483084	Mapk3	0.70	6.50E-06	1.32E-04
17451649	Acads	0.70	1.90E-05	3.10E-04
17281419	Fkbp3	0.70	1.96E-04	1.92E-03
17222606	Mfsd9	0.70	4.57E-04	3.71E-03
17315718	Ptger4	0.70	1.38E-03	8.92E-03
17366141	Prdx3	0.70	4.84E-05	6.37E-04
17460941	Slc6a6	0.70	4.38E-05	5.94E-04
17528673	Myzap	0.70	3.48E-04	2.99E-03
17467575	Thnsl2	0.70	6.01E-05	7.55E-04
17386700	Nfe2l2	0.70	5.58E-06	1.19E-04
17217155	Mfsd4	0.70	5.33E-05	6.84E-04
17327223	Fam165b	0.70	1.24E-04	1.34E-03
17240598	1700021F05Rik	0.70	4.00E-05	5.54E-04
17232332	Arhgap18	0.70	6.28E-06	1.30E-04
17339991	Eml4	0.70	6.87E-06	1.36E-04
17259810	Inpp5j	0.70	5.56E-05	7.08E-04
17443818	0910001L09Rik	0.70	3.92E-04	3.27E-03
17282743	Tgfb3	0.70	1.39E-03	8.97E-03
17280310	Id2	0.70	6.12E-05	7.64E-04
17233039	Rtn4ip1	0.70	2.19E-04	2.10E-03
17251746	1810027O10Rik	0.70	1.33E-04	1.42E-03
17396056	Fabp5	0.70	3.88E-04	3.24E-03
17518458	Igdcc4	0.70	1.35E-04	1.43E-03
17300998	Shisa2	0.70	4.56E-04	3.70E-03
17333347	Sod2	0.70	1.05E-04	1.18E-03
17336199	Cd320	0.70	1.58E-03	9.96E-03
17352212	Zadh2	0.70	3.32E-05	4.80E-04
17344200	Ly6g6d	0.70	3.80E-04	3.20E-03
17508445	Hgsnat	0.70	1.07E-03	7.28E-03
17278345	Glrx5	0.70	1.78E-05	2.95E-04
17451171	Hscb	0.70	2.57E-04	2.38E-03
17380222	Pck1	0.70	1.52E-03	9.66E-03
17404821	Ccna2	0.70	1.02E-05	1.88E-04
17382363	Noxa1	0.70	3.00E-04	2.68E-03
17252767	1300001I01Rik	0.70	6.01E-04	4.58E-03
17432227	Fblim1	0.70	3.33E-05	4.80E-04
17519910	Ttk	0.70	3.91E-04	3.26E-03
17469089	Mrps25	0.70	5.42E-04	4.23E-03
17375841	Stard7	0.70	6.08E-05	7.60E-04
17514135	Egln1	0.70	3.95E-05	5.49E-04
17453935	Zan	0.70	4.86E-04	3.88E-03
17282021	AI463170	0.70	2.86E-05	4.28E-04
17539560	Asb11	0.70	8.91E-04	6.29E-03
17372614	Timm10	0.70	7.25E-05	8.73E-04

17319150	Baiap2l2	0.70	2.00E-04	1.96E-03
17233811	Dna2	0.70	3.47E-04	2.98E-03
17425566	Ptpn3	0.69	4.18E-05	5.72E-04
17242046	Dip2a	0.69	1.30E-05	2.29E-04
17395897	Znf512b	0.69	4.93E-06	1.08E-04
17473367	Suv420h2	0.69	1.05E-03	7.19E-03
17381791	Cubn	0.69	3.69E-04	3.13E-03
17360462	Acsl5	0.69	1.43E-05	2.48E-04
17252887	Rpa1	0.69	1.70E-04	1.73E-03
17543587	Zmym3	0.69	8.04E-04	5.78E-03
17371847	Pdk1	0.69	5.64E-04	4.37E-03
17434475	Hspa8	0.69	1.68E-06	4.69E-05
17448376	9130230L23Rik	0.69	2.60E-05	3.96E-04
17509083	Fam149a	0.69	2.56E-04	2.37E-03
17503397	Man2b1	0.69	1.17E-04	1.29E-03
17265156	Slc16a13	0.69	3.10E-04	2.74E-03
17414861	C630043F03Rik	0.69	1.60E-04	1.65E-03
17450366	Hsd17b11	0.69	7.90E-06	1.51E-04
17375641	Dut	0.69	9.66E-05	1.11E-03
17339840	Ccdc75	0.69	7.46E-05	8.94E-04
17520905	Slco2a1	0.69	3.47E-04	2.99E-03
17259374	Mrpl12	0.69	2.48E-05	3.81E-04
17424319	Sigmar1	0.69	1.73E-04	1.75E-03
17432256	Slc25a34	0.69	2.81E-04	2.55E-03
17261944	Pttg1	0.69	3.46E-05	4.95E-04
17330523	Atp6v1a	0.69	2.54E-05	3.88E-04
17428659	Nasp	0.69	2.17E-05	3.45E-04
17397360	D3Ertd751e	0.69	5.87E-04	4.50E-03
17329105	Abcc5	0.69	1.36E-04	1.44E-03
17374648	Ivd	0.69	1.31E-03	8.59E-03
17255534	Hoxb13	0.69	7.39E-05	8.87E-04
17219848	Exo1	0.69	1.27E-04	1.37E-03
17360440	Adra2a	0.69	1.03E-04	1.17E-03
17313926	Gramd4	0.69	1.11E-03	7.51E-03
17385272	Arl5a	0.69	1.26E-05	2.23E-04
17247225	Upp1	0.69	6.45E-05	7.94E-04
17361352	Ccs	0.69	4.09E-05	5.64E-04
17273447	Sectm1b	0.69	6.14E-05	7.66E-04
17293607	Ptch1	0.69	1.75E-04	1.76E-03
17225785	Dtymk	0.69	9.00E-05	1.05E-03
17466397	Epha1	0.69	5.81E-05	7.32E-04
17494596	Tpp1	0.69	8.71E-06	1.64E-04
17525263	Aplp2	0.69	1.64E-05	2.78E-04
17410578	Gm16500	0.69	3.20E-04	2.80E-03
17515617	Igsf9b	0.69	2.63E-05	3.99E-04

17279724	Macc1	0.69	1.50E-04	1.57E-03
17436345	Depdc5	0.69	6.75E-04	5.02E-03
17418592	Clspn	0.69	4.03E-04	3.34E-03
17458734	Plekha8	0.69	1.64E-04	1.68E-03
17541036	Ndufa1	0.68	5.82E-06	1.22E-04
17457960	Tas2r143	0.68	7.86E-04	5.67E-03
17255543	Gm53	0.68	1.21E-04	1.32E-03
17317327	Mtss1	0.68	2.44E-06	6.14E-05
17318617	Tonsl	0.68	2.38E-04	2.24E-03
17301262	Rp1l1	0.68	4.65E-04	3.76E-03
17506398	9330133O14Rik	0.68	4.72E-05	6.26E-04
17527520	Scamp5	0.68	3.94E-05	5.48E-04
17411081	Prkacb	0.68	2.27E-05	3.57E-04
17242207	Pcbp3	0.68	1.20E-03	7.98E-03
17464146	Cmas	0.68	2.52E-06	6.29E-05
17496645	Dctpp1	0.68	1.84E-04	1.84E-03
17397475	Mgst2	0.68	9.82E-06	1.82E-04
17354810	Afap1l1	0.68	8.45E-05	9.93E-04
17255999	Erbb2	0.68	1.92E-05	3.13E-04
17300251	Abhd4	0.68	2.63E-04	2.42E-03
17507668	2410022L05Rik	0.68	2.27E-04	2.17E-03
17495373	Plekha7	0.68	2.07E-04	2.01E-03
17481087	Stim1	0.68	5.48E-05	7.00E-04
17517001	Usp28	0.68	1.09E-03	7.41E-03
17402072	Frrs1	0.68	3.23E-05	4.69E-04
17274099	Fam49a	0.68	7.09E-04	5.23E-03
17303568	Thoc7	0.68	4.62E-06	1.02E-04
17233001	Pdss2	0.68	1.10E-03	7.47E-03
17363790	Insl6	0.68	9.98E-04	6.90E-03
17501104	Dctd	0.68	1.95E-04	1.92E-03
17463051	Lpar5	0.68	6.37E-05	7.88E-04
17358682	Minpp1	0.68	8.87E-05	1.04E-03
17425043	Trim14	0.68	3.48E-04	2.99E-03
17431478	Grhl3	0.68	2.16E-04	2.08E-03
17386757	Prkra	0.68	7.10E-05	8.57E-04
17267584	Akap1	0.68	3.94E-05	5.48E-04
17273200	Pcyt2	0.68	2.98E-04	2.67E-03
17374245	Slc12a6	0.68	4.53E-04	3.68E-03
17217116	Rab7l1	0.68	9.30E-05	1.08E-03
17350364	LOC100861902	0.68	8.23E-05	9.72E-04
17521422	Hyal2	0.68	4.36E-05	5.92E-04
17529169	Cox7a2	0.68	2.10E-06	5.51E-05
17226550	Mcm6	0.68	1.61E-05	2.74E-04
17356041	Acy3	0.68	3.14E-05	4.60E-04
17230153	Fh1	0.68	6.39E-06	1.31E-04

17287466	Higd2a	0.68	5.65E-06	1.20E-04
17527433	C230081A13Rik	0.68	5.84E-04	4.49E-03
17425770	Ptgr1	0.68	5.10E-04	4.03E-03
17396143	Car13	0.68	1.65E-05	2.79E-04
17507955	Atp7b	0.68	4.88E-04	3.89E-03
17409343	5330417C22Rik	0.68	2.34E-04	2.21E-03
17453304	Gtf2i	0.68	1.20E-05	2.16E-04
17341660	Prss30	0.68	7.66E-04	5.55E-03
17336921	Ly6g6c	0.68	2.78E-04	2.53E-03
17225460	Rab17	0.68	1.71E-04	1.73E-03
17343164	Tff3	0.68	5.72E-06	1.20E-04
17468532	Gmcl1	0.68	2.52E-05	3.86E-04
17338472	Mocs1	0.68	2.91E-04	2.62E-03
17548057	AA667203	0.68	8.20E-06	1.56E-04
17265672	Spns2	0.68	4.66E-04	3.76E-03
17337852	Clic5	0.68	2.08E-05	3.34E-04
17231591	Zc3h12d	0.68	7.76E-05	9.25E-04
17223685	Ndufs1	0.68	1.12E-05	2.03E-04
17444876	Pomp	0.68	1.43E-04	1.50E-03
17475975	Ech1	0.68	5.55E-06	1.18E-04
17267846	Abcc3	0.68	1.97E-05	3.19E-04
17435934	Khk	0.68	1.52E-03	9.68E-03
17220432	Tlr5	0.68	2.02E-04	1.97E-03
17282689	Acyp1	0.67	1.68E-05	2.83E-04
17335966	Cyp4f16	0.67	1.58E-03	9.98E-03
17284170	2010107E04Rik	0.67	3.78E-04	3.19E-03
17291361	Aldh5a1	0.67	5.88E-04	4.51E-03
17469259	Suclg2	0.67	3.39E-06	7.95E-05
17386621	Atp5g3	0.67	5.71E-06	1.20E-04
17509682	BC030870	0.67	2.09E-04	2.03E-03
17461978	Cand2	0.67	1.26E-03	8.27E-03
17356794	Men1	0.67	2.19E-05	3.47E-04
17290301	Akr1e1	0.67	9.46E-04	6.60E-03
17342029	Ndufb10	0.67	5.21E-06	1.12E-04
17459014	Abcg2	0.67	9.27E-05	1.07E-03
17346802	Ndufv2	0.67	2.25E-05	3.54E-04
17400375	Ctss	0.67	1.37E-05	2.39E-04
17252200	Kif1c	0.67	5.82E-06	1.22E-04
17509629	Sc4mol	0.67	7.67E-06	1.48E-04
17243022	Btbd2	0.67	6.15E-05	7.66E-04
17472023	Mansc1	0.67	1.47E-05	2.54E-04
17524930	Acp5	0.67	1.74E-05	2.90E-04
17264382	Wdr16	0.67	1.64E-04	1.68E-03
17336602	Pbx2	0.67	1.68E-04	1.71E-03
17224433	Ihh	0.67	3.68E-05	5.18E-04

17233771	Neurog3	0.67	1.11E-03	7.52E-03
17423544	Wwp1	0.67	2.46E-05	3.80E-04
17416460	Yipf1	0.67	1.02E-04	1.16E-03
17525329	St3gal4	0.67	5.30E-06	1.14E-04
17446793	Slc5a6	0.67	1.37E-03	8.89E-03
17430080	Eif2c1	0.67	1.30E-04	1.40E-03
17404180	Car1	0.67	1.82E-06	4.94E-05
17253133	Fam57a	0.67	3.30E-06	7.77E-05
17443535	Ufsp1	0.67	1.07E-03	7.29E-03
17531591	Nbeal2	0.67	6.03E-05	7.56E-04
17508633	Wrn	0.67	2.01E-04	1.96E-03
17426198	Hdhd3	0.67	5.25E-06	1.13E-04
17423854	Slc35a1	0.67	9.80E-05	1.12E-03
17237681	9230105E05Rik	0.67	6.89E-05	8.35E-04
17334846	Tmem8	0.67	3.59E-06	8.29E-05
17340831	Slc22a1	0.67	1.86E-04	1.85E-03
17518672	Fam96a	0.66	4.16E-05	5.70E-04
17475734	Fcgbp	0.66	3.19E-05	4.65E-04
17219156	3110045C21Rik	0.66	3.10E-04	2.74E-03
17360084	As3mt	0.66	5.81E-06	1.22E-04
17295588	Naip5	0.66	3.81E-04	3.20E-03
17315677	BC037032	0.66	1.47E-04	1.54E-03
17404109	Impa1	0.66	6.45E-06	1.32E-04
17282074	Rab15	0.66	5.15E-04	4.06E-03
17346311	2410015M20Rik	0.66	1.11E-04	1.24E-03
17309688	A2ld1	0.66	9.14E-05	1.06E-03
17288876	Arrdc3	0.66	2.37E-05	3.70E-04
17230918	Cenpf	0.66	8.39E-06	1.59E-04
17282836	Vipar	0.66	2.93E-05	4.36E-04
17277043	Dcaf4	0.66	4.43E-05	5.98E-04
17216620	Tfcp2l1	0.66	1.88E-05	3.08E-04
17235354	Gm19876	0.66	3.58E-06	8.29E-05
17287486	Cdhr2	0.66	2.24E-06	5.76E-05
17438423	Paics	0.66	1.14E-04	1.26E-03
17458911	Fkbp9	0.66	9.83E-06	1.82E-04
17407926	Mrps21	0.66	4.12E-05	5.67E-04
17265657	Smtnl2	0.66	3.95E-04	3.29E-03
17411103	Lphn2	0.66	1.23E-04	1.33E-03
17318587	Slc39a4	0.66	6.85E-07	2.35E-05
17433944	Ttll10	0.66	3.04E-04	2.70E-03
17359156	Tmem20	0.66	3.08E-05	4.53E-04
17542654	Lage3	0.66	3.12E-05	4.57E-04
17351446	Impa2	0.66	3.54E-04	3.02E-03
17460073	Bola3	0.66	7.15E-04	5.26E-03
17414434	Ugcg	0.66	3.00E-05	4.44E-04

17259907	Tcn2	0.66	5.37E-06	1.15E-04
17415973	Leprot	0.66	2.02E-06	5.32E-05
17520560	Tfdp2	0.66	1.66E-04	1.70E-03
17518780	Car12	0.66	6.73E-05	8.20E-04
17500996	Acsl1	0.66	1.26E-05	2.23E-04
17377498	Pygb	0.66	6.34E-06	1.30E-04
17407378	Lor	0.66	4.98E-04	3.95E-03
17444068	Nudt1	0.66	1.68E-04	1.71E-03
17461684	Il17re	0.66	7.46E-04	5.43E-03
17324643	Bdh1	0.66	2.34E-05	3.67E-04
17426631	3110001D03Rik	0.66	2.60E-06	6.45E-05
17392549	9030622O22Rik	0.66	4.67E-04	3.77E-03
17350684	Gm4221	0.66	7.42E-04	5.41E-03
17415606	Nfia	0.66	4.65E-05	6.19E-04
17324733	1500031L02Rik	0.66	1.65E-04	1.68E-03
17466908	Hibadh	0.66	5.74E-05	7.26E-04
17248666	Rnf145	0.66	7.31E-06	1.42E-04
17251728	Zbtb4	0.66	5.18E-04	4.09E-03
17527054	Atm	0.66	2.04E-04	1.99E-03
17518238	Calml4	0.66	1.11E-06	3.35E-05
17361073	Gstp1	0.66	1.09E-06	3.32E-05
17258451	1110017F19Rik	0.66	1.09E-05	1.99E-04
17513010	Clec18a	0.65	5.85E-04	4.49E-03
17344336	Tcf19	0.65	4.96E-05	6.48E-04
17423915	Mob3b	0.65	1.86E-05	3.06E-04
17381864	Trdmt1	0.65	1.50E-04	1.57E-03
17430630	Zcchc17	0.65	2.85E-04	2.57E-03
17414277	Zfp462	0.65	4.77E-04	3.82E-03
17453506	2010001M06Rik	0.65	1.77E-06	4.84E-05
17286791	Tmem170b	0.65	1.25E-04	1.35E-03
17528418	Lactb	0.65	5.63E-04	4.36E-03
17346461	Slc25a23	0.65	3.15E-04	2.77E-03
17236878	Galnt4	0.65	1.91E-06	5.12E-05
17516960	Cadm1	0.65	9.40E-05	1.08E-03
17265026	Kctd11	0.65	4.37E-05	5.93E-04
17354730	Slc26a2	0.65	1.86E-05	3.06E-04
17527123	Acat1	0.65	7.14E-06	1.40E-04
17325770	Gm608	0.65	2.89E-05	4.32E-04
17241789	Cisd1	0.65	3.46E-05	4.95E-04
17273292	Dcxr	0.65	7.62E-07	2.53E-05
17514424	Casp1	0.65	4.54E-06	1.01E-04
17437765	Klb	0.65	4.18E-04	3.44E-03
17356314	Mrpl11	0.65	2.52E-06	6.29E-05
17286487	Bphl	0.65	6.75E-05	8.21E-04
17338701	Chaf1a	0.65	6.40E-05	7.90E-04

17376480	Mavs	0.65	2.83E-04	2.56E-03
17477774	Hrc	0.65	7.86E-04	5.67E-03
17350869	Isoc1	0.65	2.71E-04	2.49E-03
17290275	Akr1c12	0.65	1.00E-05	1.84E-04
17473856	Lig1	0.65	4.74E-05	6.28E-04
17497612	Echs1	0.65	1.41E-04	1.48E-03
17472868	Tm7sf3	0.65	5.09E-06	1.10E-04
17338747	Uhrf1	0.65	1.70E-05	2.85E-04
17524535	Dnmt1	0.65	1.71E-06	4.74E-05
17498695	2900053A13Rik	0.65	9.64E-06	1.80E-04
17306501	Ppp1r3e	0.65	1.94E-04	1.91E-03
17435170	6030443J06Rik	0.65	4.33E-04	3.54E-03
17236882	Dusp6	0.64	1.55E-06	4.39E-05
17481102	Rrm1	0.64	5.98E-05	7.52E-04
17450354	Hsd17b13	0.64	7.26E-04	5.32E-03
17265306	Slc25a11	0.64	6.99E-07	2.39E-05
17463909	Mgst1	0.64	6.74E-06	1.35E-04
17380199	Rbm38	0.64	1.84E-04	1.84E-03
17505718	Pdpr	0.64	3.48E-05	4.97E-04
17240303	G630090E17Rik	0.64	1.51E-03	9.63E-03
17304141	Ppifos	0.64	1.04E-04	1.18E-03
17230550	Lbr	0.64	1.04E-06	3.19E-05
17434614	4930420K17Rik	0.64	6.32E-05	7.84E-04
17308132	Slc25a37	0.64	8.36E-05	9.85E-04
17369896	St6galnac6	0.64	2.06E-05	3.31E-04
17226232	Ptpn4	0.64	1.05E-04	1.18E-03
17220794	D730003I15Rik	0.64	1.03E-03	7.06E-03
17417146	Pdzk1ip1	0.64	3.52E-06	8.18E-05
17228375	Xpr1	0.64	2.86E-06	6.98E-05
17498897	Tnfsf13b	0.64	9.24E-04	6.49E-03
17248242	Snrnp25	0.64	5.27E-04	4.13E-03
17304794	Hacl1	0.64	1.05E-04	1.18E-03
17253512	2610507B11Rik	0.64	1.04E-06	3.19E-05
17323828	B3gnt5	0.64	1.15E-05	2.08E-04
17440954	Mvk	0.64	4.68E-05	6.22E-04
17462180	Zfp239	0.64	2.61E-04	2.40E-03
17507637	Rasa3	0.64	1.10E-03	7.48E-03
17499396	Fbxo25	0.64	2.59E-05	3.94E-04
17429441	Guca2b	0.64	4.47E-05	6.02E-04
17219356	Tstd1	0.64	6.40E-07	2.25E-05
17328432	Mcm4	0.64	9.95E-06	1.84E-04
17420064	Tcea3	0.64	4.06E-04	3.36E-03
17512221	Cmtm4	0.64	1.61E-05	2.74E-04
17267685	Pctp	0.64	5.56E-06	1.18E-04
17530141	Pccb	0.64	4.68E-05	6.22E-04

17530669	Acy1	0.64	1.16E-04	1.28E-03
17343505	Cyp4f13	0.63	2.35E-04	2.22E-03
17365098	Scd1	0.63	9.33E-05	1.08E-03
17216753	Mgat5	0.63	4.12E-05	5.67E-04
17516462	Thy1	0.63	1.19E-03	7.93E-03
17359769	Sema4g	0.63	5.05E-06	1.09E-04
17355915	Cpt1a	0.63	7.47E-06	1.44E-04
17449033	Ppat	0.63	1.30E-04	1.40E-03
17214368	Cyp27a1	0.63	1.34E-04	1.43E-03
17375557	Sema6d	0.63	5.49E-05	7.01E-04
17509101	Tlr3	0.63	5.62E-05	7.14E-04
17303442	Oit1	0.63	7.14E-06	1.40E-04
17542239	Cetn2	0.63	2.72E-05	4.11E-04
17283303	Ccdc88c	0.63	4.47E-05	6.02E-04
17273304	Cbr2	0.63	6.58E-06	1.34E-04
17387464	2700094K13Rik	0.63	5.00E-06	1.09E-04
17463169	Scnn1a	0.63	5.66E-07	2.03E-05
17495673	Gm19963	0.63	6.47E-04	4.87E-03
17324794	Tctex1d2	0.63	1.56E-04	1.62E-03
17391233	Itpripl1	0.63	3.97E-05	5.51E-04
17351168	Htr4	0.63	1.22E-03	8.09E-03
17253599	Aldoc	0.63	6.62E-06	1.34E-04
17297576	Adk	0.63	3.04E-06	7.33E-05
17509462	Galnt7	0.63	1.77E-06	4.84E-05
17243576	Appl2	0.63	1.94E-06	5.16E-05
17215029	2810459M11Rik	0.63	2.40E-04	2.25E-03
17362595	Fads2	0.63	4.69E-05	6.23E-04
17509976	Slc25a42	0.63	6.93E-06	1.37E-04
17211396	Tmem14a	0.63	1.16E-03	7.81E-03
17517828	Cyp11a1	0.63	3.11E-04	2.74E-03
17541417	Aifm1	0.63	8.37E-06	1.59E-04
17509146	Snx25	0.63	2.30E-06	5.88E-05
17293547	Fbp2	0.63	3.32E-06	7.79E-05
17229466	Hsd17b7	0.63	3.57E-06	8.27E-05
17440257	Pcgf3	0.63	6.53E-07	2.28E-05
17469864	1500001M20Rik	0.63	6.62E-07	2.30E-05
17350517	Hsd17b4	0.63	9.85E-06	1.82E-04
17321871	Krt84	0.63	6.38E-04	4.82E-03
17441949	Aldh2	0.63	7.69E-07	2.53E-05
17470394	B4galnt3	0.63	4.41E-05	5.96E-04
17489223	Cox6b1	0.63	3.71E-06	8.52E-05
17215395	Dgkd	0.62	3.13E-05	4.58E-04
17293362	Ctla2a	0.62	1.31E-03	8.55E-03
17524014	Endod1	0.62	2.54E-06	6.33E-05
17487681	Zfp109	0.62	2.81E-05	4.21E-04

17366511	Phyh	0.62	1.20E-06	3.58E-05
17258569	Exoc7	0.62	4.75E-04	3.81E-03
17341280	Gm19747	0.62	2.82E-05	4.23E-04
17245466	Tbc1d30	0.62	3.05E-04	2.71E-03
17282158	Rdh11	0.62	2.09E-05	3.34E-04
17521875	Slc25a20	0.62	4.94E-06	1.08E-04
17229007	Fmo4	0.62	1.35E-04	1.43E-03
17216960	Pigr	0.62	4.17E-06	9.42E-05
17361546	Cst6	0.62	1.19E-04	1.31E-03
17502348	Zfp961	0.62	1.32E-04	1.41E-03
17523309	Vipr1	0.62	1.34E-05	2.35E-04
17219489	Pex19	0.62	9.78E-05	1.12E-03
17494822	Nlrp10	0.62	1.20E-05	2.16E-04
17286046	4932702P03Rik	0.62	2.40E-05	3.73E-04
17338934	Alkbh7	0.62	1.07E-04	1.19E-03
17277939	9030617O03Rik	0.62	2.47E-05	3.81E-04
17213179	Ndufb3	0.62	2.00E-04	1.96E-03
17452378	Rad9b	0.62	5.65E-04	4.37E-03
17505455	Phlpp2	0.62	1.21E-05	2.16E-04
17543264	Maged1	0.62	1.11E-06	3.35E-05
17350090	Sh3rf2	0.62	4.37E-06	9.77E-05
17246700	Gal3st1	0.62	6.73E-05	8.20E-04
17302161	1300010F03Rik	0.62	5.72E-06	1.20E-04
17279701	Cdca7l	0.62	4.35E-04	3.55E-03
17511263	Best2	0.62	3.66E-04	3.11E-03
17402485	4930422G04Rik	0.62	1.97E-04	1.94E-03
17490889	0610005C13Rik	0.62	9.48E-05	1.09E-03
17525548	Ei24	0.62	1.96E-06	5.20E-05
17518331	Tipin	0.62	6.07E-05	7.60E-04
17510707	Lsm6	0.62	7.04E-06	1.38E-04
17345124	Enpp4	0.62	1.87E-04	1.86E-03
17511878	Pllp	0.62	3.41E-06	7.98E-05
17407561	Snx27	0.62	2.01E-04	1.96E-03
17405908	Bche	0.62	3.84E-05	5.37E-04
17266960	Ccl6	0.62	8.10E-04	5.82E-03
17533234	Sytl5	0.62	5.66E-05	7.17E-04
17223490	Tmem237	0.62	4.02E-05	5.55E-04
17237208	Csrp2	0.62	2.81E-07	1.21E-05
17340285	Msh2	0.62	4.67E-04	3.77E-03
17427961	Cpt2	0.62	5.54E-07	2.01E-05
17339313	Epb4.1l3	0.62	1.82E-05	3.02E-04
17460393	Fam136a	0.61	2.46E-04	2.30E-03
17379037	Plcg1	0.61	1.63E-05	2.77E-04
17247948	Efemp1	0.61	6.56E-04	4.91E-03
17459676	Retsat	0.61	3.53E-06	8.20E-05

17527565	Csk	0.61	7.12E-06	1.39E-04
17474130	Ceacam9	0.61	1.34E-03	8.73E-03
17241318	Hk1	0.61	4.71E-05	6.25E-04
17235748	Aes	0.61	1.82E-07	8.58E-06
17484710	B4galnt4	0.61	6.50E-05	7.99E-04
17542149	Ids	0.61	5.71E-06	1.20E-04
17393173	Pxmp4	0.61	9.38E-05	1.08E-03
17421828	Clstn1	0.61	3.23E-07	1.35E-05
17218349	Glul	0.61	4.32E-07	1.69E-05
17310882	Pgcp	0.61	1.12E-04	1.24E-03
17236339	Gnptab	0.61	6.16E-04	4.68E-03
17374238	Nop10	0.61	3.65E-06	8.43E-05
17255540	Gm11538	0.61	3.67E-04	3.12E-03
17520851	Anapc13	0.61	3.64E-07	1.47E-05
17369841	Fam102a	0.61	1.63E-06	4.55E-05
17233347	Gja1	0.61	1.93E-06	5.16E-05
17217247	Pik3c2b	0.61	2.17E-05	3.45E-04
17407354	S100a1	0.61	1.04E-06	3.19E-05
17216339	Tnfrsf11a	0.61	1.41E-05	2.46E-04
17307041	Gm10873	0.61	6.50E-07	2.28E-05
17242140	4930483K19Rik	0.61	9.36E-04	6.56E-03
17409668	Cdc14a	0.61	1.54E-06	4.38E-05
17289497	Ccdc125	0.60	3.14E-04	2.76E-03
17408509	Atp1a1	0.60	8.51E-08	4.67E-06
17222777	Slc40a1	0.60	1.18E-05	2.13E-04
17502191	Mrpl34	0.60	1.20E-06	3.59E-05
17435855	Mapre3	0.60	6.24E-05	7.76E-04
17340845	Igf2r	0.60	4.60E-05	6.16E-04
17411323	Acadm	0.60	4.64E-07	1.75E-05
17498847	Lass4	0.60	5.01E-06	1.09E-04
17489214	Upk1a	0.60	6.40E-04	4.83E-03
17221786	1110058L19Rik	0.60	4.96E-06	1.08E-04
17246113	Slc39a5	0.60	5.10E-04	4.03E-03
17410939	Clca3	0.60	1.77E-06	4.84E-05
17535434	Nsdhl	0.60	8.92E-07	2.85E-05
17275223	Immp2l	0.60	4.80E-04	3.84E-03
17226302	Dbi	0.60	8.31E-07	2.68E-05
17292262	Tpmt	0.60	2.26E-04	2.16E-03
17383588	Ccbl1	0.60	1.26E-03	8.30E-03
17383139	Abo	0.60	1.36E-03	8.80E-03
17443985	Cyp2w1	0.60	1.05E-04	1.18E-03
17286462	Nqo2	0.60	1.97E-05	3.19E-04
17329167	Clcn2	0.60	1.86E-06	5.04E-05
17507368	3930402G23Rik	0.60	1.61E-04	1.66E-03
17526982	Gm684	0.60	4.80E-04	3.84E-03

17486818	Gltscr1	0.60	1.65E-05	2.79E-04
17464210	Lrmp	0.60	3.66E-04	3.11E-03
17234795	Gm19722	0.60	2.16E-06	5.63E-05
17434490	Steap4	0.60	1.71E-04	1.73E-03
17428545	Uqcrh	0.60	3.10E-06	7.43E-05
17319823	1700001L05Rik	0.60	2.51E-05	3.84E-04
17451987	Tpcn1	0.60	2.38E-06	6.01E-05
17468486	Pcyox1	0.59	3.23E-06	7.65E-05
17224738	Ap1s3	0.59	1.70E-06	4.73E-05
17233669	Pcbd1	0.59	1.03E-06	3.18E-05
17544453	Trmt2b	0.59	2.25E-06	5.77E-05
17481066	Pgap2	0.59	7.18E-06	1.40E-04
17448308	Ugdh	0.59	2.78E-07	1.20E-05
17460933	Lsm3	0.59	3.82E-06	8.70E-05
17533269	Tspan7	0.59	2.65E-06	6.52E-05
17464672	Slc25a13	0.59	6.31E-05	7.84E-04
17388016	Olfr1269	0.59	3.53E-05	5.02E-04
17243183	Creb3l3	0.59	3.05E-04	2.71E-03
17429954	1700029G01Rik	0.59	6.50E-06	1.32E-04
17313877	Ttc38	0.59	4.63E-05	6.18E-04
17382384	A830007P12Rik	0.59	4.76E-07	1.79E-05
17393592	Ndrg3	0.59	4.05E-07	1.61E-05
17524156	4931406C07Rik	0.59	1.76E-07	8.35E-06
17256632	Wnk4	0.59	5.66E-07	2.03E-05
17340943	Mrpl18	0.59	4.07E-07	1.62E-05
17244506	Mrpl42	0.58	5.05E-05	6.55E-04
17310637	Fam134b	0.58	7.45E-06	1.44E-04
17300735	Atp12a	0.58	2.13E-07	9.78E-06
17497713	Ifitm2	0.58	1.83E-05	3.03E-04
17517112	Gm19447	0.58	1.25E-07	6.50E-06
17526149	Pdzd3	0.58	1.34E-03	8.74E-03
17520326	Plscr2	0.58	8.01E-04	5.77E-03
17307905	Dpysl2	0.58	3.32E-05	4.80E-04
17524944	Zfp599	0.58	1.15E-03	7.73E-03
17361299	2010003K11Rik	0.58	4.85E-06	1.06E-04
17419773	Stmn1	0.58	3.22E-05	4.68E-04
17298364	Nt5dc2	0.58	2.89E-05	4.32E-04
17472085	Pbp2	0.58	1.14E-04	1.26E-03
17527963	Paqr5	0.58	1.02E-07	5.44E-06
17546119	Prps2	0.58	1.77E-05	2.94E-04
17215097	B3gnt7	0.57	2.15E-04	2.07E-03
17243330	Nfic	0.57	6.03E-06	1.25E-04
17429008	St3gal3	0.57	2.03E-06	5.35E-05
17498467	Ano1	0.57	1.99E-06	5.25E-05
17457504	Rab19	0.57	4.78E-06	1.05E-04

17340197	Epas1	0.57	2.07E-06	5.44E-05
17363559	Fam189a2	0.57	2.86E-04	2.58E-03
17231717	Fuca2	0.57	1.94E-07	9.05E-06
17345004	LOC100862363	0.57	1.46E-06	4.17E-05
17472530	Kcnj8	0.57	1.79E-04	1.80E-03
17276520	Mthfd1	0.57	1.76E-05	2.93E-04
17360331	Mxi1	0.57	2.72E-07	1.19E-05
17339554	Lbh	0.57	7.69E-08	4.41E-06
17388667	B230118H07Rik	0.57	1.68E-04	1.71E-03
17443539	Ephb4	0.57	3.27E-06	7.71E-05
17480740	Ucp2	0.57	4.26E-08	2.81E-06
17387430	D030029J20Rik	0.57	1.75E-04	1.76E-03
17454121	Agfg2	0.57	6.70E-06	1.35E-04
17442780	Glt1d1	0.57	5.06E-05	6.55E-04
17443185	Mlxipl	0.56	2.15E-06	5.62E-05
17417784	2610528J11Rik	0.56	1.88E-05	3.08E-04
17520976	Tmem108	0.56	1.51E-03	9.60E-03
17423622	2610029I01Rik	0.56	2.78E-05	4.19E-04
17288903	Mblac2	0.56	3.86E-05	5.38E-04
17252497	Atp2a3	0.56	1.01E-06	3.11E-05
17321683	Slc11a2	0.56	1.22E-05	2.18E-04
17268227	Abi3	0.56	6.64E-07	2.31E-05
17220529	Mir194-1	0.56	2.36E-04	2.22E-03
17517390	Idh3a	0.56	3.00E-06	7.26E-05
17458718	Prr15	0.56	4.31E-07	1.69E-05
17526843	Sdhd	0.56	2.36E-07	1.06E-05
17253648	Slc46a1	0.56	1.99E-06	5.25E-05
17409736	Slc35a3	0.56	9.26E-07	2.92E-05
17276297	2210039B01Rik	0.56	4.11E-04	3.39E-03
17241921	Gstt3	0.56	2.33E-04	2.21E-03
17516098	Siae	0.56	6.33E-05	7.85E-04
17537048	Cox7b	0.56	1.69E-04	1.71E-03
17324420	St6gal1	0.56	3.55E-04	3.03E-03
17314577	Pfkm	0.56	4.80E-05	6.33E-04
17269521	Acly	0.56	9.13E-07	2.89E-05
17422659	B930041F14Rik	0.56	6.30E-05	7.83E-04
17532692	LOC100861738	0.56	2.48E-05	3.81E-04
17337844	Enpp5	0.56	1.30E-07	6.61E-06
17268120	Pdk2	0.56	4.66E-08	3.02E-06
17405827	1110032A04Rik	0.56	2.54E-07	1.12E-05
17268010	Acsf2	0.56	1.38E-06	4.01E-05
17217321	Golt1a	0.56	1.62E-06	4.54E-05
17343856	Btnl5	0.56	1.21E-05	2.16E-04
17547658	LOC100503141	0.55	1.43E-04	1.50E-03
17471222	Ccnd2	0.55	1.41E-07	6.94E-06

17457887	Tmem139	0.55	1.46E-05	2.52E-04
17450989	Pxmp2	0.55	3.90E-04	3.26E-03
17252359	Ggt6	0.55	2.24E-05	3.53E-04
17362717	Dak	0.55	1.41E-05	2.46E-04
17378359	Acss2	0.55	7.59E-07	2.53E-05
17315152	Acvr1b	0.55	7.68E-07	2.53E-05
17448821	Ociad2	0.55	9.28E-06	1.74E-04
17273864	Apob	0.55	6.35E-05	7.87E-04
17467806	Tcf7l1	0.55	4.03E-06	9.13E-05
17374738	Gchfr	0.55	8.20E-07	2.65E-05
17458338	AI854703	0.55	1.94E-05	3.16E-04
17217651	Tnnt2	0.55	1.73E-05	2.89E-04
17374455	Spred1	0.55	3.36E-06	7.88E-05
17222356	2010300C02Rik	0.55	9.62E-07	3.01E-05
17510136	Ifi30	0.55	6.67E-07	2.31E-05
17226655	C4bp	0.55	8.31E-05	9.80E-04
17338959	Tnfsf9	0.55	9.36E-05	1.08E-03
17529871	Acpl2	0.55	2.98E-05	4.42E-04
17374827	1700020I14Rik	0.55	6.31E-06	1.30E-04
17491420	Svip	0.55	7.05E-06	1.38E-04
17546259	Ube1y1	0.55	9.49E-04	6.62E-03
17468286	Cyp26b1	0.55	2.30E-04	2.18E-03
17517289	Exph5	0.54	5.04E-04	3.99E-03
17212286	Slc9a2	0.54	2.34E-07	1.05E-05
17375396	Sord	0.54	6.76E-06	1.36E-04
17466441	Fam115c	0.54	1.56E-04	1.62E-03
17231358	Iyd	0.54	1.30E-04	1.40E-03
17294135	Srd5a1	0.54	3.15E-06	7.51E-05
17458355	Gimap9	0.54	9.26E-05	1.07E-03
17425248	Tmem246	0.54	3.45E-04	2.97E-03
17404047	Zfp704	0.54	1.99E-06	5.25E-05
17449710	Cxcl9	0.54	5.79E-04	4.46E-03
17410390	Hadh	0.54	6.80E-08	4.01E-06
17360977	Lrp5	0.54	2.36E-06	5.98E-05
17353962	Fgf1	0.54	9.97E-06	1.84E-04
17440575	Chek2	0.54	5.34E-05	6.85E-04
17511714	Ces1e	0.54	4.52E-04	3.67E-03
17405142	Setd7	0.54	4.13E-07	1.63E-05
17401673	Sort1	0.54	1.26E-06	3.72E-05
17471107	Plekhg6	0.54	8.39E-06	1.59E-04
17330799	Gm5485	0.54	1.79E-07	8.48E-06
17302889	Pcca	0.54	1.74E-06	4.80E-05
17519649	Gsta4	0.54	8.93E-08	4.84E-06
17282498	Entpd5	0.54	8.29E-08	4.59E-06
17478883	Mcee	0.54	2.35E-06	5.97E-05

17231366	Plekhg1	0.54	4.48E-05	6.03E-04
17230484	Ephx1	0.53	9.57E-07	3.00E-05
17489046	Hcst	0.53	1.58E-04	1.63E-03
17322327	Cbx5	0.53	2.14E-06	5.61E-05
17211405	Gsta3	0.53	2.41E-04	2.26E-03
17327264	Cbr3	0.53	2.10E-07	9.69E-06
17467269	Hpgds	0.53	5.11E-04	4.04E-03
17500957	Lrp2bp	0.53	1.25E-03	8.25E-03
17363799	5033414D02Rik	0.53	7.79E-07	2.56E-05
17397957	P2ry1	0.53	5.04E-08	3.17E-06
17294664	Polr3g	0.53	6.82E-06	1.36E-04
17477391	Klk1	0.53	3.26E-07	1.36E-05
17520364	Plod2	0.53	2.85E-04	2.57E-03
17281084	Egln3	0.53	2.30E-07	1.04E-05
17274813	Slc26a3	0.53	9.11E-07	2.89E-05
17525578	Slc37a2	0.53	1.19E-04	1.31E-03
17238054	Ndufa4l2	0.53	1.67E-04	1.70E-03
17434524	Abcb1a	0.53	5.32E-07	1.95E-05
17489989	AI987944	0.53	8.53E-05	1.00E-03
17328277	Pla2g10	0.53	2.19E-08	1.70E-06
17284963	Marcksl1-ps4	0.52	1.05E-04	1.18E-03
17389795	Ccdc32	0.52	7.89E-07	2.58E-05
17340307	Msh6	0.52	2.29E-06	5.85E-05
17266107	Abr	0.52	7.65E-07	2.53E-05
17449394	Sult1b1	0.52	4.37E-07	1.69E-05
17225261	Ngef	0.52	1.70E-05	2.85E-04
17367916	Lrrc26	0.52	6.92E-08	4.07E-06
17243888	Chpt1	0.52	6.70E-05	8.17E-04
17252058	Tm4sf5	0.52	8.13E-07	2.64E-05
17487361	Apoc2	0.52	2.32E-05	3.65E-04
17493869	P2ry6	0.52	1.44E-05	2.50E-04
17420557	Rnf186	0.52	6.65E-06	1.34E-04
17246860	Nipsnap1	0.52	5.04E-06	1.09E-04
17283871	Bcl11b	0.52	1.13E-04	1.25E-03
17359636	Abcc2	0.52	2.56E-06	6.36E-05
17501041	Casp3	0.52	3.12E-05	4.57E-04
17388332	Creb3l1	0.52	4.11E-08	2.77E-06
17548296	LOC100503320	0.52	5.88E-08	3.58E-06
17312186	Psca	0.52	4.89E-05	6.41E-04
17251607	Trappc1	0.52	2.75E-07	1.19E-05
17388974	Depdc7	0.51	2.08E-04	2.02E-03
17501787	Tm6sf2	0.51	3.81E-06	8.70E-05
17219231	Nr1i3	0.51	4.16E-08	2.78E-06
17329220	Ehhadh	0.51	1.21E-05	2.16E-04
17388803	Cat	0.51	3.40E-08	2.41E-06

17433328	Per3	0.51	5.58E-07	2.01E-05
17241954	Gstt2	0.51	2.09E-05	3.34E-04
17309802	Oxct1	0.51	2.34E-08	1.77E-06
17376191	Tgm3	0.51	4.75E-07	1.79E-05
17495958	Ern2	0.51	2.19E-07	1.00E-05
17520353	Plscr4	0.51	8.84E-05	1.03E-03
17475725	Zfp607	0.51	2.53E-04	2.35E-03
17445860	Napepld	0.51	4.41E-07	1.70E-05
17539682	BC022960	0.51	6.65E-04	4.96E-03
17340967	Acat2	0.51	8.08E-07	2.63E-05
17289103	Serinc5	0.51	3.05E-07	1.29E-05
17496839	Pycard	0.51	1.83E-08	1.50E-06
17375997	Mertk	0.51	1.68E-04	1.71E-03
17265340	Camta2	0.50	6.23E-08	3.74E-06
17317284	Anxa13	0.50	1.50E-06	4.29E-05
17307463	Ebpl	0.50	9.76E-06	1.81E-04
17508036	Slc25a15	0.50	5.61E-06	1.19E-04
17284919	Akr1c14	0.50	4.41E-04	3.60E-03
17514799	2200002K05Rik	0.50	1.15E-07	6.06E-06
17535951	Fundc2	0.50	6.35E-05	7.87E-04
17285244	Nid1	0.50	6.25E-08	3.74E-06
17255853	Cisd3	0.50	3.71E-08	2.58E-06
17485357	Dhcr7	0.50	7.09E-07	2.42E-05
17506707	Rab4a	0.49	1.20E-05	2.16E-04
17396162	Car2	0.49	1.19E-08	1.11E-06
17496354	Sult1a1	0.49	8.37E-05	9.86E-04
17311807	Sqle	0.49	6.23E-08	3.74E-06
17527421	Tspan3	0.49	8.64E-08	4.71E-06
17476728	Cebpa	0.49	1.55E-07	7.59E-06
17327255	Cbr1	0.49	1.80E-08	1.49E-06
17251441	Slc25a35	0.49	5.12E-06	1.10E-04
17326964	Hunk	0.49	4.94E-06	1.08E-04
17484477	Prap1	0.49	1.22E-04	1.33E-03
17449406	Sult1d1	0.49	7.75E-08	4.43E-06
17539494	Ace2	0.49	7.48E-06	1.44E-04
17515315	Ldlr	0.49	1.07E-08	1.02E-06
17309557	Slc15a1	0.49	5.40E-05	6.91E-04
17227086	Gm19497	0.49	3.06E-06	7.37E-05
17295386	Tmem171	0.49	5.98E-06	1.24E-04
17220591	5033404E19Rik	0.49	5.11E-05	6.59E-04
17399769	S100a13	0.49	7.51E-08	4.37E-06
17480788	Fchsd2	0.49	5.88E-07	2.08E-05
17289324	Gcnt4	0.49	1.41E-06	4.06E-05
17433672	2810405K02Rik	0.48	2.70E-08	1.97E-06
17526307	Gm10023	0.48	1.17E-05	2.10E-04

17366670	Itih5	0.48	2.08E-08	1.63E-06
17540378	Maob	0.48	3.20E-04	2.81E-03
17272468	St6galnac2	0.48	4.07E-08	2.77E-06
17283556	Ifi27l2b	0.48	1.80E-06	4.91E-05
17313087	Mgat3	0.48	4.85E-09	5.92E-07
17357815	Fam111a	0.48	6.38E-05	7.89E-04
17440054	A830010M20Rik	0.48	2.27E-07	1.03E-05
17456736	Ahcyl2	0.48	1.53E-07	7.54E-06
17466581	Zfp467	0.48	4.04E-05	5.57E-04
17231999	Myb	0.48	4.81E-06	1.06E-04
17487001	Hif3a	0.48	7.56E-08	4.38E-06
17232135	1110021L09Rik	0.47	4.02E-08	2.76E-06
17493436	1810020D17Rik	0.47	1.24E-06	3.69E-05
17530990	Gnat1	0.47	1.61E-06	4.54E-05
17353358	Stard4	0.47	1.66E-07	8.01E-06
17291355	Acot13	0.47	1.38E-07	6.92E-06
17295233	Hmgcr	0.47	1.08E-09	2.15E-07
17318983	Tst	0.47	1.78E-08	1.48E-06
17491636	D7Ertd715e	0.47	3.50E-04	3.00E-03
17404534	Lrrc31	0.47	1.97E-05	3.19E-04
17442719	Aacs	0.47	2.56E-07	1.13E-05
17337089	H2-Q1	0.46	8.54E-06	1.61E-04
17284948	Akr1c19	0.46	1.48E-05	2.55E-04
17340135	Slc3a1	0.46	7.18E-08	4.19E-06
17534960	Fhl1	0.46	7.93E-08	4.44E-06
17536961	Chic1	0.46	2.48E-05	3.81E-04
17473580	Zfp667	0.46	1.05E-04	1.18E-03
17217625	Tnni1	0.46	7.36E-06	1.43E-04
17339910	Galm	0.46	4.11E-07	1.63E-05
17443486	Cldn15	0.46	5.53E-07	2.01E-05
17239384	Phactr2	0.46	2.18E-06	5.66E-05
17447831	Fgfbp1	0.45	1.88E-09	3.12E-07
17457853	Sval1	0.45	1.11E-06	3.35E-05
17243392	Gna11	0.45	5.28E-09	6.26E-07
17534528	Xpnpep2	0.45	8.70E-08	4.73E-06
17288467	Lpcat1	0.45	9.46E-05	1.09E-03
17321780	Cela1	0.45	1.96E-07	9.15E-06
17399936	Crnn	0.45	1.58E-04	1.63E-03
17257906	Map2k6	0.45	1.23E-05	2.19E-04
17448001	Ppargc1a	0.45	3.79E-07	1.52E-05
17277760	5430427M07Rik	0.45	1.42E-05	2.47E-04
17340427	LOC100044193	0.45	4.02E-04	3.33E-03
17267011	Gm11437	0.45	3.12E-04	2.75E-03
17367095	Tmem236	0.44	1.35E-09	2.56E-07
17235990	1500009L16Rik	0.44	2.99E-06	7.26E-05

17464539	Hepacam2	0.44	2.63E-06	6.51E-05
17466322	Trpv6	0.44	1.40E-08	1.24E-06
17248476	Pank3	0.44	2.90E-09	4.09E-07
17409130	Gstm3	0.44	6.75E-07	2.34E-05
17489801	Nudt19	0.44	1.34E-07	6.75E-06
17255466	Gngt2	0.44	1.62E-06	4.54E-05
17299559	Pnp2	0.43	2.22E-06	5.74E-05
17409154	Gstm1	0.43	1.08E-04	1.21E-03
17490589	Fcgrt	0.43	2.69E-07	1.18E-05
17284913	Tubal3	0.43	1.34E-05	2.34E-04
17354739	Ppargc1b	0.43	6.55E-08	3.87E-06
17316392	Hrsp12	0.43	2.80E-06	6.85E-05
17288716	Glrx	0.43	3.43E-07	1.41E-05
17280817	Scin	0.43	1.52E-08	1.31E-06
17351811	Acaa2	0.43	2.48E-09	3.64E-07
17448245	Tlr1	0.42	1.30E-07	6.61E-06
17307134	Cryl1	0.42	1.91E-08	1.54E-06
17525894	Sorl1	0.42	1.80E-08	1.49E-06
17363429	Aldh1a7	0.42	1.12E-04	1.24E-03
17345989	Sult1c2	0.42	2.44E-06	6.14E-05
17373283	Lrp4	0.42	1.68E-07	8.08E-06
17512732	Nqo1	0.42	3.43E-07	1.41E-05
17429057	Ptprf	0.42	1.62E-09	2.86E-07
17394694	B4galt5	0.42	4.78E-09	5.92E-07
17290163	Ccl28	0.42	7.60E-08	4.39E-06
17261963	Fabp6	0.42	1.90E-05	3.10E-04
17403070	Adh1	0.42	1.06E-08	1.02E-06
17404091	Fabp4	0.41	5.71E-04	4.41E-03
17500543	Ppp1r3b	0.41	2.42E-08	1.80E-06
17417946	Guca2a	0.41	2.41E-08	1.80E-06
17488057	Cyp2s1	0.41	4.16E-07	1.63E-05
17511731	Ces1f	0.41	8.43E-05	9.91E-04
17236182	Timp3	0.41	6.81E-07	2.35E-05
17409142	Gstm2	0.41	6.10E-06	1.26E-04
17250055	1810065E05Rik	0.41	4.13E-08	2.77E-06
17297750	Ppif	0.41	3.71E-08	2.58E-06
17346401	Acer1	0.41	4.80E-07	1.80E-05
17437459	Slc34a2	0.41	1.09E-06	3.32E-05
17496584	Zg16	0.40	8.82E-07	2.82E-05
17435584	Insig1	0.40	2.36E-09	3.59E-07
17416325	Dhcr24	0.40	3.28E-10	1.07E-07
17364150	Pank1	0.40	6.86E-07	2.35E-05
17236393	Slc5a8	0.40	7.83E-08	4.44E-06
17315002	Mettl7a1	0.40	3.05E-05	4.50E-04
17484670	Nlrp6	0.40	3.54E-07	1.45E-05

17504467	Pdp2	0.40	7.67E-07	2.53E-05
17457876	Gstk1	0.40	2.90E-06	7.04E-05
17359251	Cyp2c65	0.39	3.43E-07	1.41E-05
17324835	Tfrc	0.39	1.31E-07	6.61E-06
17294934	Thbs4	0.39	4.11E-09	5.35E-07
17364440	Cyp2c69	0.39	5.31E-05	6.82E-04
17345066	Mep1a	0.39	5.32E-09	6.26E-07
17260644	Ddc	0.38	2.32E-08	1.77E-06
17504829	Hsd11b2	0.38	7.88E-08	4.44E-06
17358664	A1cf	0.38	7.35E-07	2.47E-05
17319091	Lgals2	0.37	6.92E-06	1.37E-04
17321078	Vdr	0.37	1.49E-10	6.06E-08
17310816	Cmbl	0.37	1.01E-08	9.82E-07
17408336	Hsd3b3	0.37	8.27E-07	2.67E-05
17413674	Aldh1b1	0.37	3.77E-09	4.98E-07
17464400	Rep15	0.37	5.37E-08	3.30E-06
17273348	Fasn	0.37	1.34E-09	2.56E-07
17402558	Elovl6	0.37	5.54E-08	3.40E-06
17280062	Lpin1	0.36	4.46E-06	9.92E-05
17309785	Sepp1	0.36	3.61E-09	4.85E-07
17312567	Gpt	0.36	6.63E-06	1.34E-04
17450838	Rnf212	0.36	7.37E-07	2.47E-05
17306147	Ndrg2	0.36	4.82E-08	3.08E-06
17425609	D630039A03Rik	0.36	1.43E-06	4.10E-05
17504572	Ces2g	0.35	2.88E-06	7.02E-05
17331828	Cldn8	0.35	1.23E-06	3.65E-05
17513092	Fa2h	0.35	3.62E-10	1.11E-07
17342038	Fahd1	0.35	5.41E-09	6.31E-07
17476288	Cox7a1	0.35	1.74E-09	2.91E-07
17295278	Hexb	0.35	6.59E-09	7.15E-07
17235730	2210404O07Rik	0.35	6.33E-09	6.96E-07
17362839	Ms4a10	0.35	1.61E-09	2.86E-07
17450937	4930522L14Rik	0.35	1.99E-04	1.96E-03
17459696	Gm15401	0.34	1.27E-08	1.16E-06
17290173	Hmgcs1	0.34	5.12E-11	2.81E-08
17504512	Ces2c	0.34	3.57E-08	2.50E-06
17472114	Plbd1	0.34	1.01E-08	9.82E-07
17507605	Gas6	0.34	6.67E-10	1.57E-07
17400773	Fmo5	0.34	4.23E-08	2.81E-06
17362861	Ms4a18	0.34	4.40E-06	9.81E-05
17456161	Cav1	0.33	1.34E-07	6.75E-06
17319707	Cyp2d26	0.33	3.40E-08	2.41E-06
17348860	Mep1b	0.33	1.43E-09	2.68E-07
17325884	Cd200r2	0.32	5.26E-04	4.13E-03
17404122	Slc10a5	0.32	1.97E-07	9.15E-06

17493461	Aqp11	0.31	2.13E-10	7.84E-08
17506532	Dpep1	0.31	6.20E-09	6.90E-07
17482752	2010110P09Rik	0.31	3.85E-10	1.16E-07
17480044	Prss23	0.31	2.39E-09	3.59E-07
17539979	Glod5	0.31	2.40E-09	3.59E-07
17458407	Abp1	0.31	2.55E-10	8.99E-08
17482897	Aqp8	0.30	1.93E-08	1.54E-06
17394063	Ada	0.30	2.50E-10	8.99E-08
17253181	Tmigd1	0.30	1.95E-09	3.14E-07
17311893	Gsdmcl-ps	0.30	2.75E-07	1.19E-05
17279509	Crip1	0.30	2.78E-11	2.14E-08
17470095	Fxyd4	0.29	5.27E-07	1.94E-05
17343488	Cyp4f14	0.28	1.48E-09	2.74E-07
17526663	Fam55b	0.27	3.44E-06	8.03E-05
17520631	Rbp2	0.27	5.17E-10	1.42E-07
17355443	Lipg	0.27	1.41E-07	6.94E-06
17504541	Ces2e	0.27	4.54E-11	2.74E-08
17475870	Sycn	0.26	4.90E-09	5.92E-07
17322075	Krt4	0.26	2.23E-06	5.75E-05
17252183	Eno3	0.25	7.40E-11	3.48E-08
17322750	Abat	0.25	6.29E-09	6.95E-07
17516978	Fam55d	0.23	5.98E-09	6.74E-07
17504477	Ces2a	0.23	1.33E-06	3.89E-05
17225210	Alpi	0.23	1.73E-09	2.91E-07
17329847	Osta	0.23	2.61E-10	9.00E-08
17408360	Hao2	0.22	8.00E-10	1.78E-07
17348833	Ttr	0.22	3.18E-10	1.07E-07
17545899	S100g	0.20	5.61E-10	1.48E-07
17400862	Hmgcs2	0.20	2.46E-09	3.64E-07
17397750	Tm4sf4	0.20	3.23E-10	1.07E-07
17507829	Defb37	0.18	4.45E-06	9.92E-05
17503926	Mt4	0.18	4.14E-07	1.63E-05
17359239	Cyp2c55	0.17	3.58E-11	2.40E-08
17317442	Gsdmc2	0.17	5.57E-07	2.01E-05
17425233	Aldob	0.14	1.09E-09	2.15E-07
17360785	Pnliprp2	0.14	9.82E-10	1.98E-07
17402350	Fabp2	0.08	2.17E-13	7.08E-10

Supplementary Table 3: IL22 and tunicamycin responsive genes in mouse colonoids. 2689 genes are differentially expressed (q-value <= 0.01) after treatment with IL22 and tunicamycinin combination. The data are sorted by decreasing values of the estimated expression fold change relative to control samples.
